# Myopia-26, the female-limited form of early-onset high myopia, occurring in a European family

**DOI:** 10.1186/s13023-021-01673-z

**Published:** 2021-01-22

**Authors:** Noémi Széll, Tamás Fehér, Zoltán Maróti, Tibor Kalmár, Dóra Latinovics, István Nagy, Zsuzsanna Z. Orosz, Márta Janáky, Andrea Facskó, Zoltán Sohajda

**Affiliations:** 1 Kenézy Gyula University Hospital, Debrecen Medical University, Debrecen, Hungary; 2 Doctoral School of Clinical Medicine, University of Szeged, Szeged, Hungary; 3 Institute of Biochemistry, Biological Research Centre, Szeged, Hungary; 4 Genetic Diagnostic Laboratory, University of Szeged, Szeged, Hungary; 5 Seqomics Biotechnology Ltd, Mórahalom, Hungary; 6 Department of Ophthalmology, Faculty of Medicine, University of Szeged, Szeged, Hungary

**Keywords:** Early onset high myopia, X-linked female-limited high myopia, Intrinsically photosensitive retinal ganglion cell, Monogenic disorder, Mendelian inheritance, X-arrestin, ARR3, G-protein coupled receptor

## Abstract

**Background:**

Female-limited early-onset high myopia, also called Myopia-26 is a rare monogenic disorder characterized by severe short sightedness starting in early childhood and progressing to blindness potentially by the middle ages. Despite the X-linked locus of the mutated ARR3 gene, the disease paradoxically affects females only, with males being asymptomatic carriers. Previously, this disease has only been observed in Asian families and has not gone through detailed investigation concerning collateral symptoms or pathogenesis.

**Results:**

We found a large Hungarian family displaying female-limited early-onset high myopia. Whole exome sequencing of two individuals identified a novel nonsense mutation (c.214C>T, p.Arg72*) in the ARR3 gene. We carried out basic ophthalmological testing for 18 family members, as well as detailed ophthalmological examination (intraocular pressure, axial length, fundus appearance, optical coherence tomography, visual field- testing) as well as colour vision- and electrophysiology tests (standard and multifocal electroretinography, pattern electroretinography and visual evoked potentials) for eight individuals. Ophthalmological examinations did not reveal any signs of cone dystrophy as opposed to animal models. Electrophysiology and colour vision tests similarly did not evidence a general cone system alteration, rather a central macular dysfunction affecting both the inner and outer (postreceptoral and receptoral) retinal structures in all patients with ARR3 mutation.

**Conclusions:**

This is the first description of a Caucasian family displaying Myopia-26. We present two hypotheses that could potentially explain the pathomechanism of this disease.

## Background

Myopia or short-sightedness has become a serious world health issue recently [1]. This can be attributed to its extreme phenotypes on the „upper end of the scale”, namely high and pathologic myopia. Cases of high myopia with a rapid progression carry the risk of advancing into pathologic myopia, a condition that is associated with potentially blinding complications. There is an explicit increase in the prevalence of these conditions lately, therefore an urgent need for targeted treatments is recognized [1, 2]. To devise such treatment options however, we need to thoroughly understand the exact molecular mechanisms of refractive errors and myopia development. Albeit nearly 270 genes associated with myopia have been identified so far, the underlying pathways through which these genes influence refractive error development remain obscure in most of the cases [3].

Inheritance of late onset or common myopia and early onset high myopia (eoHM) was evidenced to differ basically yet earlier [4]. As opposed to common forms, eoHM is predominantly inherited in a Mendelian manner with one single causative, highly penetrant gene mutation, practically with minimal influence of environment or behaviour. The specific mode of inheritance of such diseases covers a wide range of forms including autosomal dominant, autosomal recessive or X-linked recessive [5]. One of the most curious and exceptional modes of transmission is that seen for Myopia-26, displaying X-linked dominant inheritance. This rare disease, described earlier only in three Asian families paradoxically affects females only, with male hemizygotes being asymptomatic (emmetropic) carriers [6]. The ARR3 gene, residing on the X-chromosome and encoding the cone-arrestin was found to be mutated in all affected patients. Associated symptoms were not reported for those cases, neither was a potential mechanism of pathogenesis provided.

Today, the general pathomechanism of refractive error development is assumed to be based on a retina-to-sclera signalling cascade guided locally by light stimuli in the retina [7]. All retinal cell types seem to participate in this retina-specific signal transduction and derailment of retinal cell physiology and light processing are the key mechanisms [3]. However, only recent advances allowed for deeper insight into the genetic background of these processes. There is still much to be discovered in this field, especially concerning the specific role of the mutated genes in pathogenesis to imply further treatment potentials. Promising is the fact that despite their different manners of inheritance, there is an overlap between eoHM and common myopia in both causative genes and pathways of pathogenesis [3].

In our study we investigated a large family of five generations displaying female-limited eoHM. Whole exome sequencing identified an early stop codon within the ARR3 gene, verifying the diagnosis of Myopia-26. In order to explore the clinical phenotype of this disease further, we accomplished thorough ophthalmological and electrophysiological testing. Electrophysiology test results altogether suggested a central macular retinal ganglion cell deficit besides the photoreceptoral disturbance, and permitted the formulation of the ganglion-cell hypothesis to explain the development of myopia, in addition to the hypothesis based on the cone-arrestin defect.

## Results

**Fig. 1:**
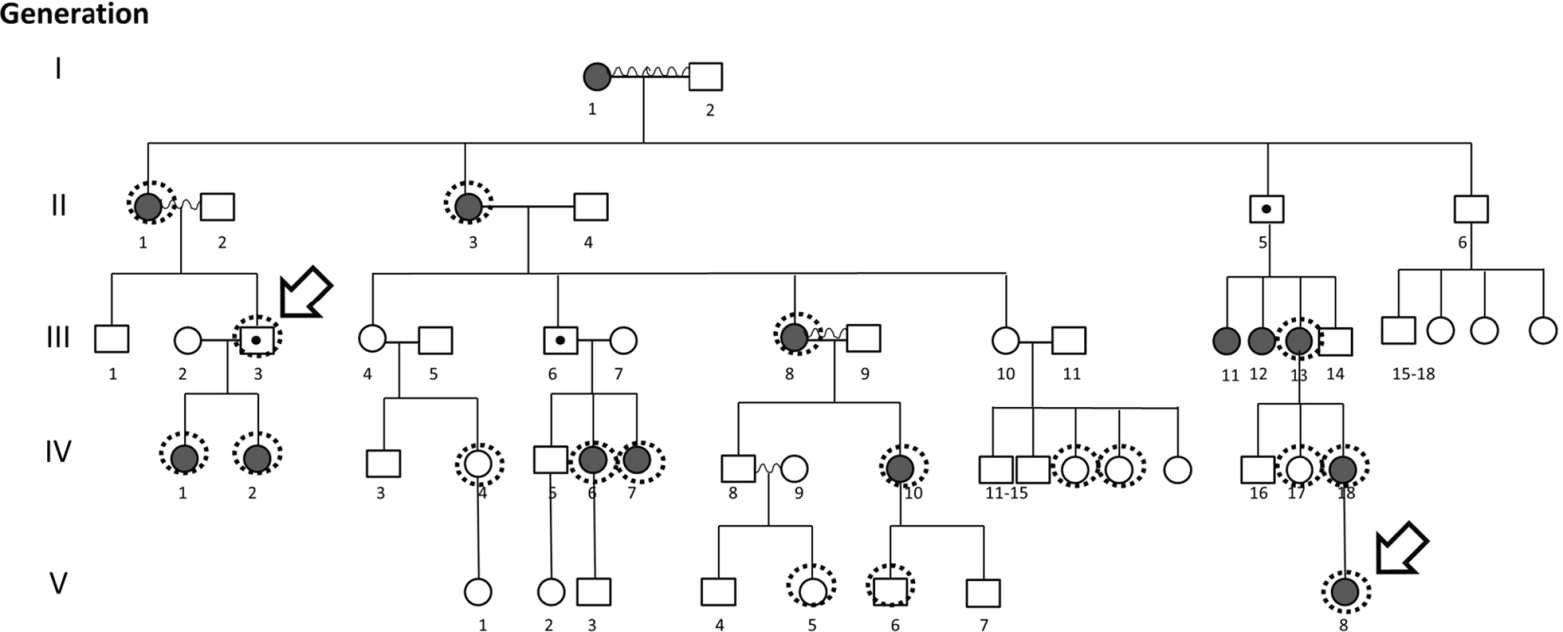
The pedigree of the investigated family. Dark shading indicates an eoHM phenotype. Dashed circles mark patients whose blood samples were obtained, the two arrows mark the two samples that went through whole exome sequencing

In the course of our routine ophthalmological work, we found multiple interrelated patients displaying eoHM. Precisely recording the personal and familial medical histories of the patients allowed the compilation of their pedigree (Fig. 1). This revealed a family of five generations comprising numerous affected patients, all of whom are females. Assuming a monogenic trait, this pattern seemed to be indicative of X-linked heredity where the mutant allele is dominant in females, but has no penetrance in males, i.e. it is female limited. We found only a single paper describing such transmission of eoHM, referred to as Myopia-26. All three reported families belonged to the East Asian ethnicity [6].

To identify the causative mutation, DNA prepared from the blood samples of patients III/3 and V/8 (a male carrier and a symptomatic female, respectively) were submitted to whole exome sequencing. We identified the same variant (NM_004312.2:c.214C>T NP_004303.2:p.Arg72Ter) in the X chromosome-based ARR3 gene in both individuals in hemizygous and heterozygous form, respectively. The presence of this candidate pathogenic variant was confirmed by conventional PCR amplification and Sanger sequencing as well. Segregation of this change with the disease was assessed for all available family members. We confirmed the presence of this nonsense variant in heterozygous state in all available symptomatic female members of the family (II/1, II/3, III/8, III/13, IV/1, IV/2, IV/6, IV/7, IV/10 and IV/18). We have also confirmed the absence of this ARR3 variant from all studied asymptomatic females (IV/4, IV/13, IV/14, IV/17 and V/5). Patient V/6, a healthy male was found to carry the wild type allele. To date, this variant has not been described in the Human Gene Mutation Database, the Exome Aggregation Consortium, the Exome Sequencing Project, ClinVar or the 1000 Genome Browser. Prediction programs Polyphen2, SIFT, and MutationTaster predicted pathogenicity of the nonsense variant. Overall, these results confirmed the diagnosis of Myopia-26.

Next, eight of our patients were exposed to a more thorough examination. Medical history revealed no other notable systemic or ophthalmological disorders relevant for this matter. The gender, age, best corrected visual acuities (BCVA), spherical equivalents (SE), intraocular pressures (IOP), axial lengths (available for patients who went through scleral reinforcement surgery), fundus appearance (classified according to the META-PM study [8]), OCT-, visual field and colour vision test results of these patients are shown in Table 1. Examples of our findings are shown in Fig. 2 and Additional file 1: Figures S2–S21.

**Table 1:** Clinical findings of the investigated family members

Genetic ID, status	Age	Refractive error: SE (dioptres)	BCVA o.d. o.s	AL (mm)	fundus appearance	OCT	Visual field (VF) (both eyes)	IOP (Hgmm)	Colour vision (both eyes)
III/3-carrier	32	E/E	20/20 20/32		META-PM0: normal retina	Normal retina	Nasal loss to 30°	21/20	Lanthony D-15: diffuse colour discrimination error
IV/1-affected	14	− 8/ − 8	20/32 20/32	26.34 / 26.24	META-PM1: tesselated retina	Mildly thinner sensory retina	Normal	12/15	Lanthony D-15: diffuse colour discrimination error
IV/2-affected	10	− 6/ − 4	20/25 20/20		META-PM0: normal retina	Normal retina	Normal	15/13	Lanthony D-15: diffuse colour discrimination error
IV/6-affected	21	− 23/ − 19	20/50 20/50	30.12 / 29.81	META-PM2: Diffuse chorioretinal atrophy Peripapillary atrophy	Incipient atrophic sensory retina	Nasal 10° loss (+ superior artefact)	20/19	ISIHARA: neg
IV/7-affected	20	− 13/ − 9.5	20/100 20/40	27.45 / 26.1	META-PM2: diffuse chorioretinal atrophy peripapillary atrophy	Incipient atrophic sensory retina	Nasal 10° loss	17/19	ISIHARA: neg
III/8-affected	48	− 14/ − 7	20/500 20/100		META-PM1-2: tesselated retina, incipient diffuse chorioretinal atrophy pale, ONH with peripapillary atrophy	Incipient atrophic sensory retina	Generalized constriction	23/21	Lanthony D-15: diffuse colour discrimination error
IV/10-affected	28	− 12.5/ − 14.5	20/63 20/125	27.02 / 26.97	META-PM1-2: tesselated retina, incipient diffuse chorioretinal atrophy peripapillary atrophy	Incipient atrophic sensory retina	Nasal 10° loss (+ superior artefact)	19/20	Lanthony D-15: diffuse colour discrimination error
V/6-healthy control	10	E/E	20/20 20/20		Normal	Normal	Normal	17/15	ISIHARA: errors made (Father has similar CVD)

*AL* axial length, *BCVA* best corrected visual acuities, *CVD* color vision defect, *E* emmetropic (with no refractive error), *IOP* intraocular pressure, *OCT* optical coherence tomography, *o.d.* right eye, *o.s.* left eye, *o.u.* both eyes, *ONH* optic nerve head, *SE* spherical equivalent, *VEP* visual evoked potentials, *META-PM* meta analyses of pathologic myopia

**Fig. 2:**
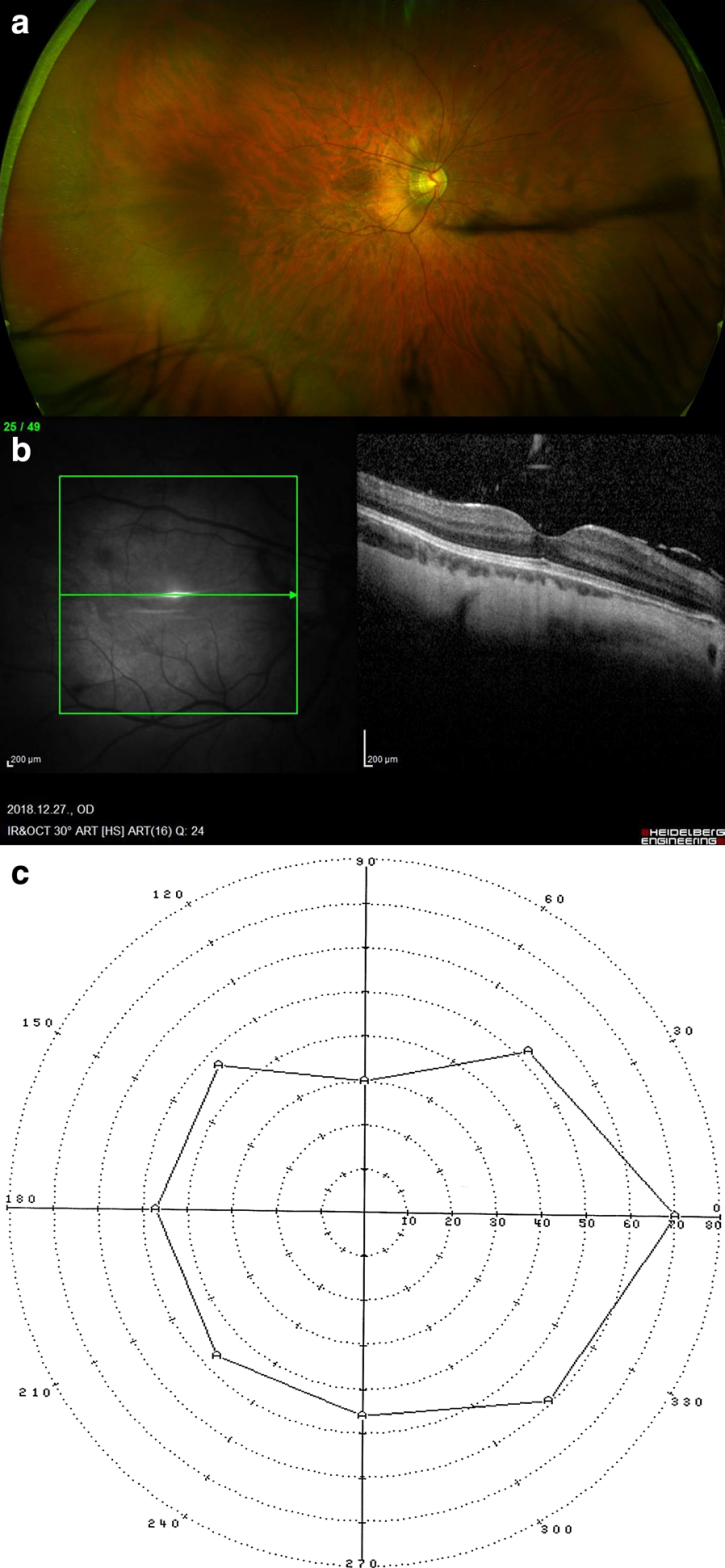
**a** Ultra widefield (Optos® California) fundus image of the right eye of affected female patient IV/6 displaying a META-PM2 stage myopic fundus. The tesselated appearence of the retina along with peripapillary and diffuse chorioretinal atrophy is observable. **b** Macular OCT image of the right eye affected female IV/6 displaying thinner (incipient atrophic) sensory retina and posterior vitreous detachment characteristic of higher degrees of myopia. **c** Visual field of the right eye of affected female IV/6 (nasal loss + superior artefact)

Numerical values extracted from the electrophysiological test results are shown in Additional file 3: Tables S1, S2 and S3 of the Supplementary text. Examples of standard full-field electroretinography (ERG) recordings are shown in Fig. 3, pattern electroretinography (PERG) in Fig. 4, pattern visual evoked potentials (pVEP) in Fig. 5, and multifocal electroretinography (mfERG) in Fig. 6. All remaining recordings are available in Additional file 2: Figures S22–S55.

**Fig. 3:**
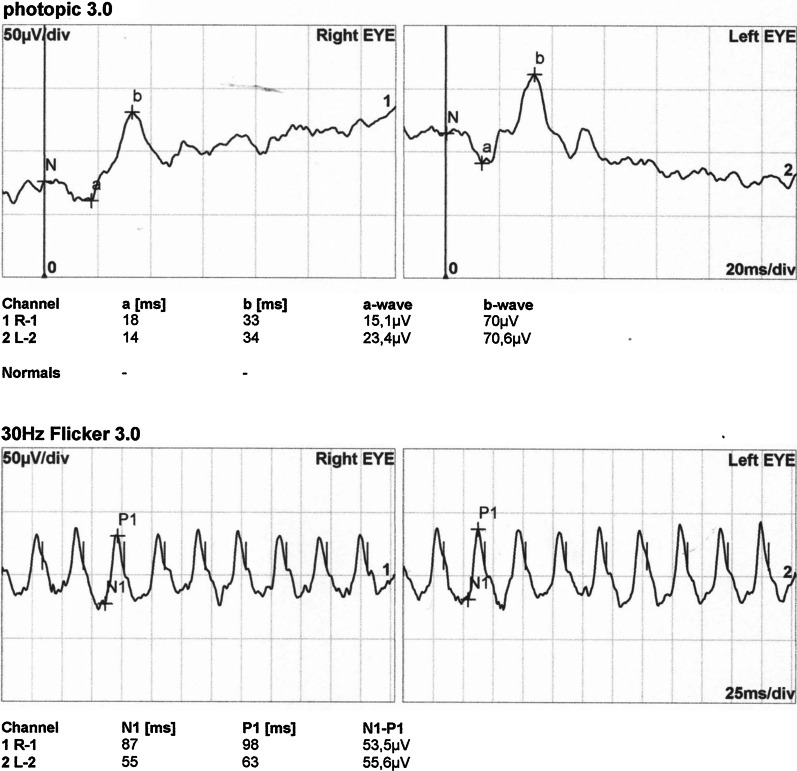
Normal photopic 3.0 ERGs in affected female IV/7. Despite prominent phenotypic signs of eoHM (SE: − 13.0/ − 9.0D, impaired BCVA, high myopic fundus alterations) in IV/7 individual, photopic 3.0 ERGs show no alterations, reflecting an overall normally functioning cone system

**Fig. 4:**
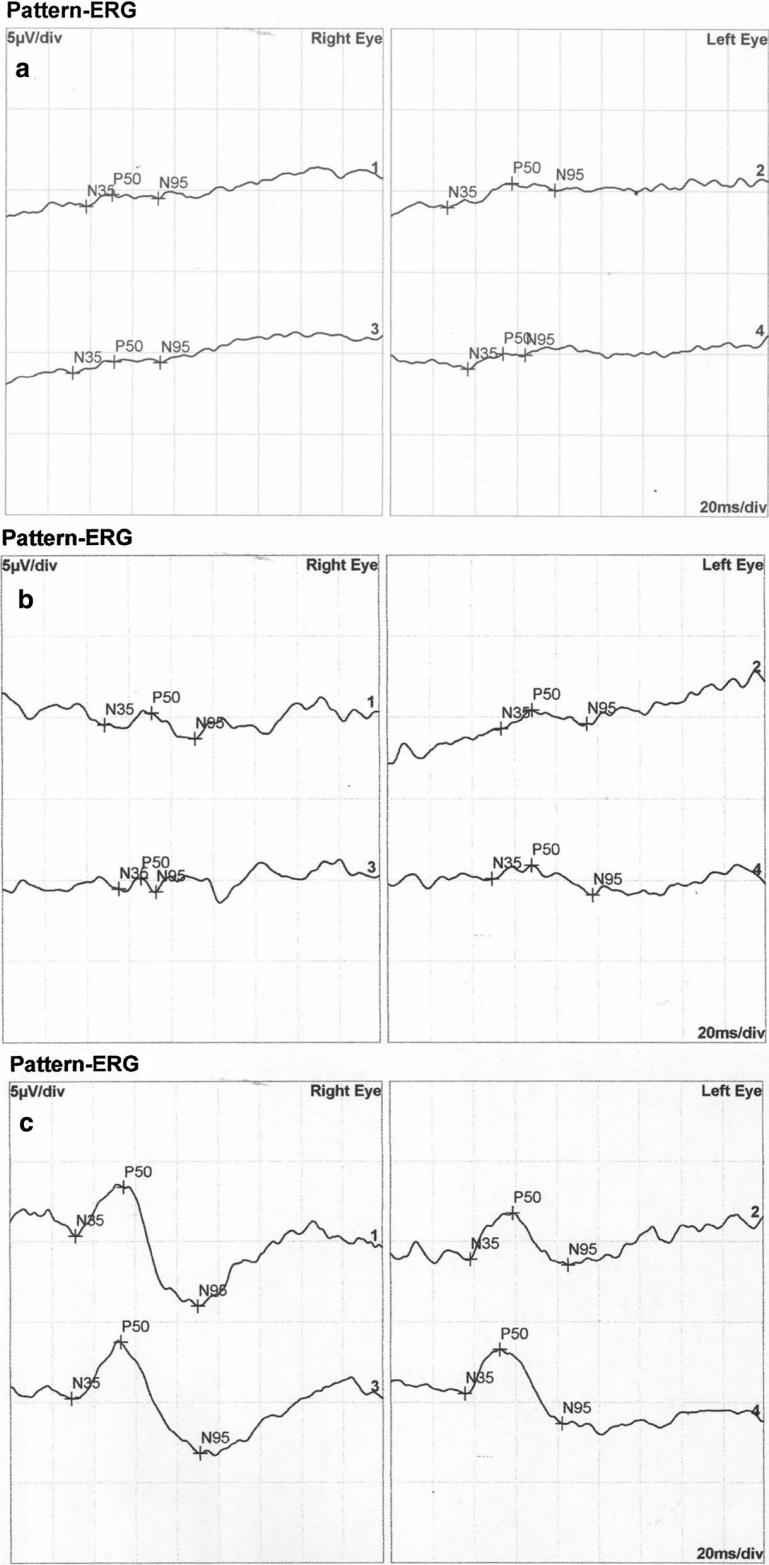
**a** Pattern ERG of carrier male III/3 is heavily affected. Despite no phenotypic sign of eoHM and visual impairment, pattern ERG of the carrier male patient is similarly subnormal as those of affected female patients. **b** Heavily affected PERG recordings of affected female IV/7. **c** Pattern ERG of unaffected male V/6. Physiological wave patterns are detected. In all sections, lines 1 and 3 and lines 2 and 4 represent pairs of replicate measurements

**Fig. 5:**
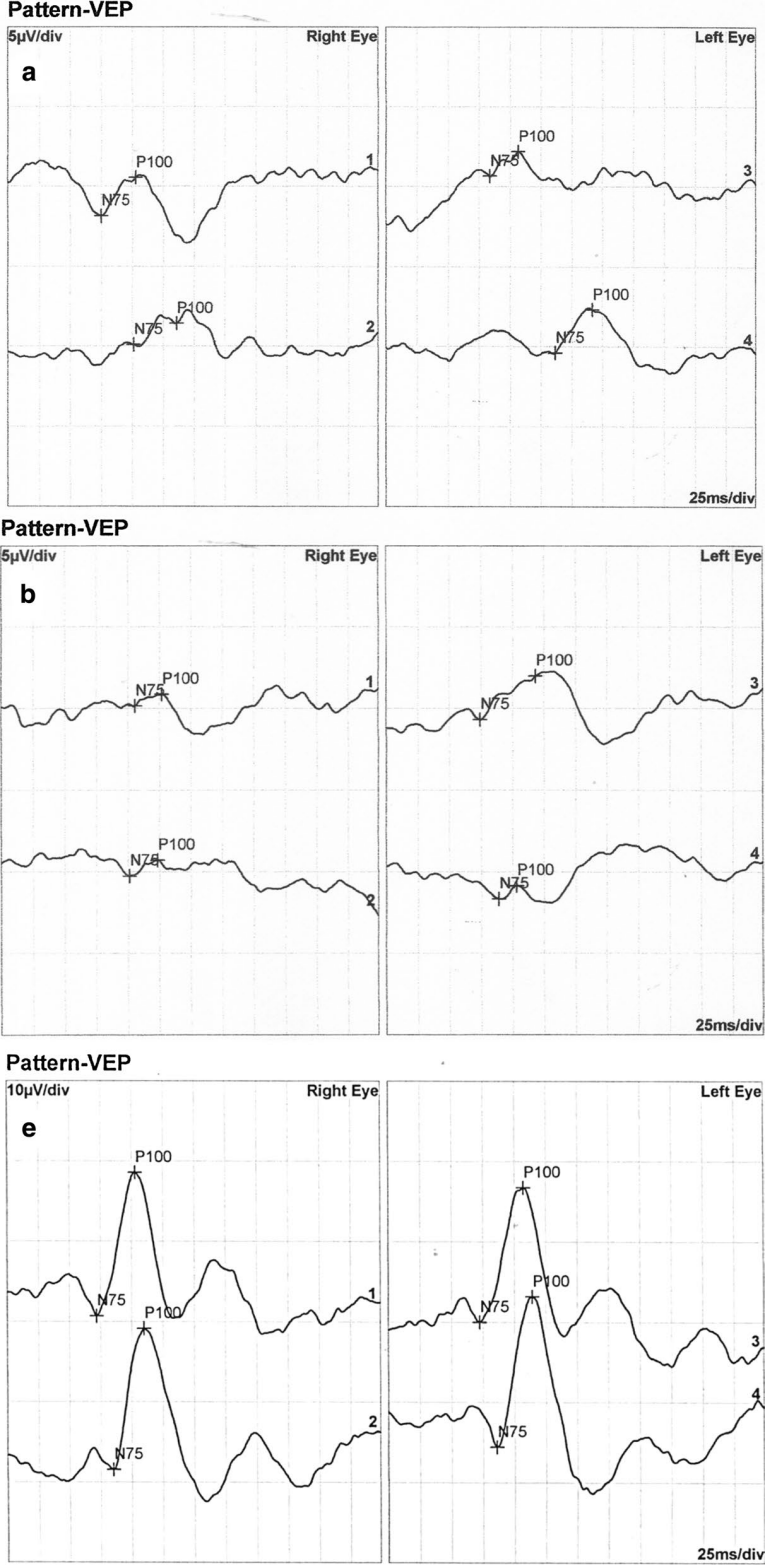
**a** Pattern VEP recordings of patient III/3 demonstrating increased implicit times and decreased amplitudes of P100 for 15′ (smaller checks) stimulation as compared to normal control. **b** Heavily affected pVEP recordings of affected female IV/7 demonstrating increased peak times and decreased amplitudes of P100. **c** Normal pattern VEP recordings of unaffected male V/6 (Note the change of the voltage scale). In all sections, lines 1 and 3 display responses to 60′ stimuli and lines 2 and 4 represent responses to 15′ stimuli

**Fig. 6:**
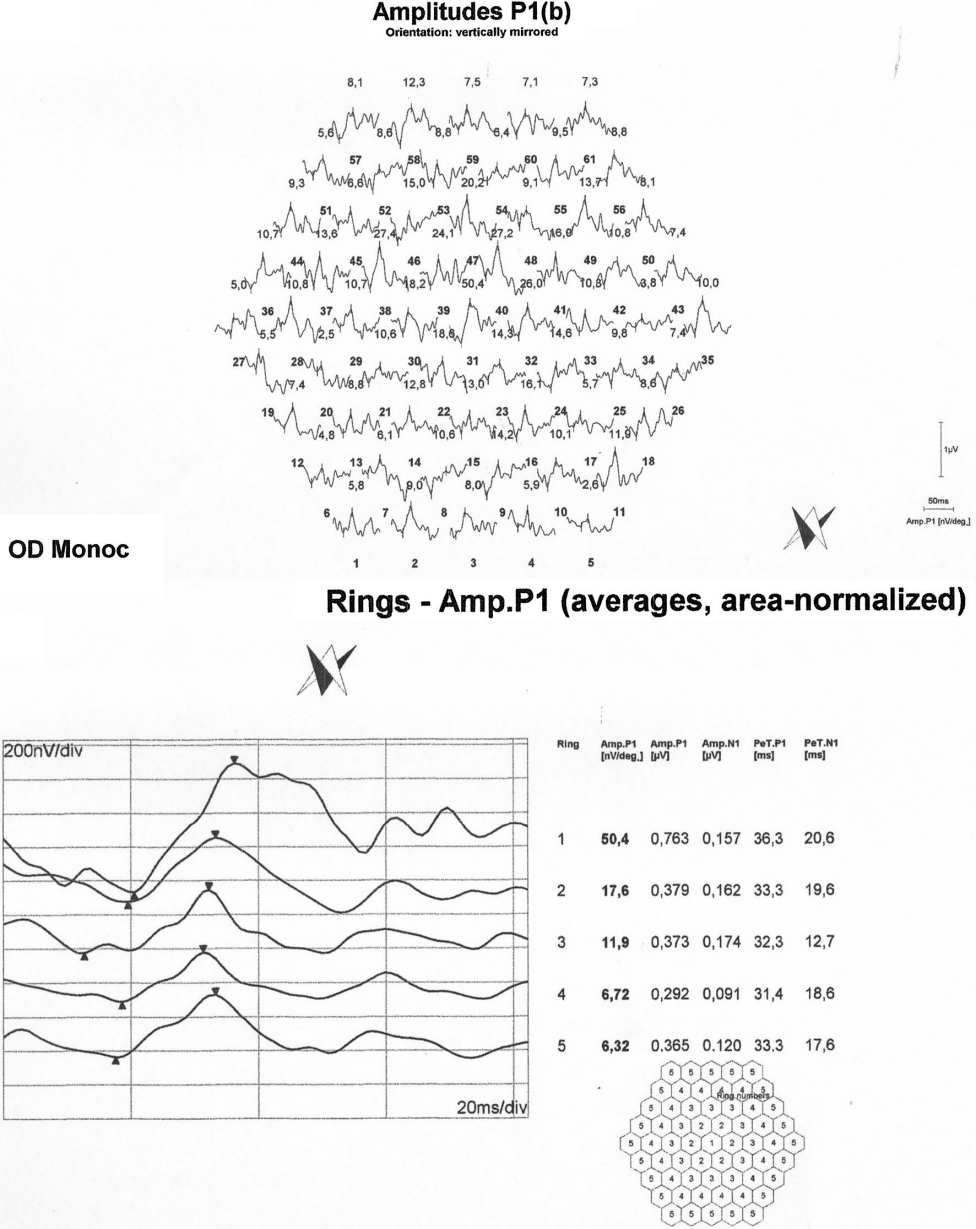
**a** MfERG recording of carrier male III/3, raw waveform. **b** MfERG recording and ring analysis of carrier male III/3

Some points of note:

1. Fundus, OCT and visual field alterations showed no characteristics of cone dystrophy, such as „bull’s eye” appearance on the central fundus, outer retinal changes with OCT or a central scotoma with visual field testing. Rather they were characteristic of high myopia: META-PM1-2 fundus appearance (See Additional file 3: Supplementary text and Additional file 1) and thinner or incipient atrophic sensory retina on macular OCT scans (Fig. 2).
2. Electrophysiology test results overall indicated a macular dysfunction in our patients with ARR3 mutation apparently affecting both the inner and outer retinal structures of the central retina (Figs. 3, 4, 5, 6), as opposed to a generalized cone dysfunction expected based on X-arrestin knockout animal models [9]. These electrophysiological alterations (detailed in the Additional file 3: Supplementary text) were detected in all patients with ARR3 mutation irrespective of their affected or carrier genetic status, and at the same time showed no correlation with either the VA, SE or the age of the patients. Accordingly, these alterations are most likely attributable to the genetic defect itself, and are not secondary consequences of the high myopic refractive error.
3. Colour vision test results revealed a diffuse colour vision discrimination error with no specific axis in our patients tested with the Lanthony Desaturated D-15-hue Panel test. This is again consistent with the central macular deficit suggested by the electrophysiology tests of our patients (see Additional file 3: Supplementary text).
4. Despite the fact that the possibility of an association of POAG with high myopia in our patients arose (detailed in the Additional file 3: Supplementary text), available data do not provide sufficient and inarguable evidence to support the diagnosis of POAG at present. Long- term follow-up will be necessary to reveal any evidence of potential progression of these parameters that could also be expected in glaucoma.

## Discussion

In this study, we report a family displaying a heritable form of eoHM, where the disease is manifested only in females. Compilation of the pedigree permitted the identification of carrier males, and revealed that their female offspring are exclusively affected, which suggested an X-linked dominant, female-limited inheritance. Whole exome sequencing of two individuals indeed revealed a nonsense-mutation within the coding region of a gene on the X-chromosome, namely ARR3. Sanger sequencing of the respective locus in a total of 16 female family members unveiled a perfect correlation between the presence of the mutant allele and the high myopia phenotype. This is the first report of a mutation in ARR3 causing hereditary eoHM, called Myopia-26 in a Caucasian family. Three Chinese families have been reported earlier to display a similar, X-linked dominant, female-limited transmission of eoHM [6]. In those cases the ARR3 was found to carry c.893C>A (p.Ala298Asp), c.298C>T (p.Arg100*) and c.239T>C (p.Leu80Pro) mutations, respectively. The mutant allele identified in our study (c.214C>T, p.Arg72*) is therefore novel. The earlier publication on Myopia-26 lacked a detailed phenotypic description of the patients, and did not attempt to explain the pathomechanism of the disease. Our main goals from this point onwards were therefore to carry out a thorough ophthalmologic investigation of the family and use the acquired information, along with literature data to build reasonable hypotheses on the molecular mechanism of pathogenesis.

ARR3 encodes a 388 amino acid-long visual arrestin with multiple names (Arrestin 3, Arrestin 4, Cone-arrestin, Retinal cone arrestin-3, X-arrestin), we refer to it as X-arrestin. Besides its key role in the phototransduction process in retinal cones, it is also expressed in pinealocytes of the pineal gland [10]. Arrestins make up an important family of proteins, with the primary function of desensitizing phosphorylated G-protein coupled receptors (GPCRs). Arrestin 1 and X-arrestin bind to opsins (hence called visual arrestins), while β-arrestin 1 and 2 bind to numerous other types of GPCRs. Arrestin 1 has very high preference for opsins found in retinal rods and cones, whereas X-arrestin has a fairly high binding capacity to non-opsin binding partners as well, and therefore has more diverse synaptic roles [11].

Our knowledge about the function and cell type-specific expression of X-arrestin is, at this time based mostly on experimental data derived from animal models. X-arrestin is expressed in all cone types of the human retina [12], however it displays a weaker expression in the S-cones of mice [13]. Arrestin 1, on the other hand is detectable in rods and S-cones of baboons, but not in LM cones [14]. In the cones of knockout mice, Arrestin-1 seems to provide a functional replacement for X-arrestin [15]. This experimental dataset allows us to formulate two reasonable, albeit incomplete hypotheses on the pathogenesis of myopia in ARR3-mutant patients. We refer to these as the cone- and the ganglion cell-hypothesis, respectively. The cone-hypothesis assumes that Arrestin-1 expression in humans is present in S-cones, but not in LM cones, as seen in baboons [14], so an X-arrestin defect would lead to limited arrestin function in LM, but not in S cones. Since arrestins are responsible for the desensitization of opsins, decreased arrestin function in LM-cones would mean their increased activity, and the “sensitization” to red/green visual stimuli. Such selective cone dysfunction could explain the onset of myopia the following way. The physical phenomenon of chromatic aberration leads to shorter wavelengths forming an image in a more anterior, and longer wavelengths forming an image in a more posterior plane (Figure S1A). Normally, the measure of luminance contrast is maximized during accommodation, and long-wavelengths form an image behind the photoreceptors. In patients with a relatively increased sensitivity of L-cones, the posterior image will produce a stronger stimulus (Figure S1B). As a result, a higher luminance contrast will be attained upon increased accommodation and by ocular elongation, two hallmarks of myopia pathogenesis [16]. Although accommodation excess in itself may not be sufficient to cause myopia [17], the phenomenon of image-forming behind the retina, called hyperopic defocus has been shown to provoke ocular elongation in numerous animal studies [18, 19]. Briefly, since blue light is claimed to have a protective effect against myopia, the *relative* weakening of the blue light stimulus upon the loss of X-arrestin can explain the eventual development of myopia in these patients [20].

The selectively altered function of various cone types, however, cannot be tested with standard photopic 3.0 ERGs. Due to the quite extensively overlapping spectral sensitivities of different photopigments [21], these tests reflect the summed activity of all three retinal cone types. Photopic 3.0 ERGs indeed, were normal and showed no alteration in our patients (Fig. 3). L, M and S-cones responses can be isolated electrophysiologically by recording the light adapted ON/OFF-ERG and the S-cone ERG. Similar to the PhNR, these recordings are an extension of the full-field ERG [22] which enable characterisation of the different cone types, including bipolar cell interactions.

Our ganglion cell-hypothesis attributes the development of refractive error to the dysfunction of retinal ganglion cells (RGC). To better understand this connection, one must acknowledge that apart from their primary role of transmitting visual information from photoreceptors to higher cerebral visual centres, a subset of RGCs called intrinsically photosensitive retinal ganglion cells (ipRGCs) have an additional role [23]. As their name suggests, they can detect light directly through their photosensitive protein called melanopsin. At the same time, they also transduce the signal originating from rod and cone photoreceptor cells, analogously to classical RGCs [24]. Classical and ipRGCs are interconnected horizontally by amacrine cells, which allow them to influence the activity of one another [25]. IpRGCs and their light sensitive protein, melanopsin are primarily responsible for non-image forming visual functions such as circadian rhythms or pupil reactions [26–28]. They have recently been discovered to play a role in conscious, image-forming visual perception as well [27]. Eye development is connected to both image-forming and non-image forming light detection pathways and accordingly refractive error may be a consequence of the derailment of either.

There is an increasing body of evidence supporting that in the image-forming pathway, light plays a key role in emmetropization and refractive error development, and besides the intensity, the spectral composition of the light stimulus is just as crucial [29, 30]. As opposed to opsins, melanopsin is most sensitive to shorter wavelengths of the spectrum, i.e. blue light [31]. Besides the anti-myopic effect of blue light attributed to the myopic defocus it causes on the retina (discussed above) [20], it has a further protective effect mediated in part by dopamine through pre- and postsynaptic connections of ipRGCs to dopaminerg amacrin cells [32]. Dopamine has been long acknowledged as a retinal neurotransmitter acting against myopia development, and it has also been evidenced that blue light stimulates a larger amount of dopamine release than other wavelengths do [32]. Accordingly, a disruption of ipRGC function may result in the alteration of the wavelength composition of the perceived light with a chromatic aberration shifted towards longer wavelengths of the spectrum, along with decreased dopaminergic activity. Both issues reduce the protective effect of blue light against myopia, potentially leading to the development of a progressive refractive error.

The non-image forming visual functions of ipRGCs, such as circadian rhythm photoentrainment also play an important role in eye development [33]. IpRGCs and melanopsin mediate circadian cycles both endogenously in the retina (again, through dopamine release) and via a systemic route comprising the hypothalamic suprachiasmatic nucleus (SCN) and the pineal gland through the inhibition of melatonin release in pinealocytes [33]. The circadian clock influences ocular development, and disruption of the circadian cycle has been found to elongate eye components and yield myopia in various myopia models [34]. Therefore, either the primary defect of ipRGCs or the primary dysfunction of pinealocytes (or both) could cause the refractive error seen in our patients. Although the prior is difficult to explain (discussed below), the latter (pineal malfunction) is highly probable due to the fact that pinealocytes normally express the X-arrestin. Melatonin, the product of pinealocytes has been shown to inhibit retinal dopamine synthesis [35], modulate D_2_ dopamine-receptor expression in the retina of chicks [36] and abolish diurnal cycling of dopamine levels in goldfish retina [37]. These observations could strongly support the possibility that pinealocyte malfunction caused by ARR3 mutations lead to altered (probably increased) melatonin levels, which in turn cause myopia by impairing the diurnal rhythms of the eye.

Currently, the most obviously missing piece of both the cone- and the ganglion cell-hypothesis is the cause of RGC dysfunction displayed on the PERG recordings. Direct linkage to the ARR3 mutation would require ARR3 expression in RGCs, which was not detectable in mice [15]. However, the promoter of the human ARR3 and its murine orthologue are markedly different, which may result in disparate cell type specific expression as well [11]. Another possibility would be the secondary malfunction of RGCs, resulting from the altered activity of pinealocytes. This could be mediated by the humoral control of retinal dopaminerg transmission by the pineal gland (described above), or the direct effect of melatonin on RGCs via their MT_1_ and MT_2_ melatonin receptors [38]. The details of this control are currently missing, it is nevertheless noteworthy that myopes have higher melatonin levels than non-myopes [39]. Finally, altered cone function, resulting from reduced X-arrestin levels may also negatively influence RGC activity. We nevertheless have no reason to believe that the cone- and the ganglion cell hypotheses are mutually exclusive, or exclude other pathomechanisms.

Another major shortcoming of both the cone- and the ganglion cell hypothesis is the lack of explanation for the female-limited heredity pattern of myopia. It is especially curious that the central macular dysfunction seems to be present also in males, without leading to eoHM. We assume the presence of a “rescue mechanism” in males, or in other words, the lack of a pathological process that would lead to an axial length elongation in response to the central retinal dysfunction. Sex-dependent differences in retina function have been described in mice, and the risk of certain retinal diseases have been shown to be sex hormone-dependent in humans [40]. Further physiology and molecular biology studies are required however to unveil the exact mechanisms responsible for the observed female-limited phenotype. Such research may also shed light on why the mutant allele is dominant in females. In the course of molecular studies however, the limitations of animal models must always be kept in mind, despite their great value. For example, an age related cone dystrophy was suggested in Arr4^−/−^ mice (Arr4 being the murine orthologue of ARR3) based on immune-histochemical findings and the pronounced diminishment in photopic flash and flicker ERGs [9]. In contrast, no generalized cone dysfunction could be evidenced in our patients carrying ARR3 mutation, either male or female, according to the electrophysiological and ophthalmological phenotypic characterization.

From the clinical point of view, our next investigative steps seem well defined: i) cone-specific ERGs (S-cone ERGs and ON/OFF ERGs) to isolate individual (L, M, or S) cone responses [41] and thus support or exclude our selective cone dysfunction hypothesis; ii) post-illumination pupil response (PIPR) to test melanopsin expressing ipRGC function [21] and thus shed light on the extent of ipRGC damage. iii) long-term follow-up of the progression of a potential POAG monitoring IOPs, visual field defects, optic nerve head appearances and RNFL OCTs.

## Conclusions

Using whole exome sequencing, we identified the pathogenic mutation of the female-limited early onset high myopia observed in our patients to be a premature stop codon in the ARR3 gene. This illustrates that contrary to its current classification [42], female-limited eoHM, also referred to as Myopia-26 is not limited to the East Asian ethnicity.

## Methods

### Patients and ethical approval

In our genetic study of eoHM we investigated a five-generation family displaying numerous affected individuals in each generation. Blood samples were taken from 18 family members representing four generations, eight of whom went through comprehensive ophthalmological and electrophysiological testing. Written informed consent was obtained from all individual participants included in the study. This study was approved by the National Scientific and Research Ethics Committee of the Medical Research Council of Hungary (ETT TUKEB, registration number 58542-1/2017/EKU). All procedures performed in studies involving human participants were in accordance with the ethical standards of the National Scientific and Research Ethics Committee and with the 1964 Helsinki declaration and its later amendments or comparable ethical standards.

### Genetic analyses

Whole exome sequencing (WES) of two family members (asymptomatic male III/3, and symptomatic female V/8) was performed. Human genomic DNA was prepared from blood samples using the MagCore Genomic Whole Blood Kit (RBC Bioscience, New Taipei City, Taiwan), according to manufacturer’s instructions. Genomic capture was carried out with SureSelect XT Human All Exon + UTRs v.5 Exome Kit (Agilent, Santa Clara, CA). Massively parallel sequencing was done using NextSeq500 Sequencer (Illumina, San Diego, CA) in combination with the NextSeq™ 500 High Output Kit (1 × 150 bp). Raw sequence data analyses, including base calling, de-multiplexing, alignment to the hg19 human reference genome (Genome Reference Consortium GRCh37), and variant calling, were performed using an in-house bioinformatics pipeline. For variant filtration, all disease-causing variants reported in HGMD®, ClinVar, or in CentoMD® as well as all variants with minor allele frequency (MAF) of less than 1% in ExAc database were considered. Variants that possibly impair the protein sequence, i.e., disruption of conserved splice sites, missense, nonsense, read-throughs, or small insertions/deletions, were prioritized. All relevant inheritance patterns were considered. The candidate pathogenic mutation (NM_004312.2:c.214C>T NP_004303.2:p.Arg72Ter) was verified by PCR amplification and Sanger sequencing for both individuals. Next, the same was done to test for the presence of this allele in all remaining DNA samples obtained from the family. The predicted pathogenicity of the variant identified in this study was tested with Polyphen2, SIFT, and MutationTaster.

### Clinical investigation

Clinical assessment included comprehensive ophthalmological examination and electrophysiological testing. Patients’ own and family medical history was registered regarding other ophthalmological disorders than eoHM as well as any systemic diseases. Best corrected visual acuity (BCVA) was recorded (Snellen chart) and refractive error expressed as spherical equivalent (SE). High myopia was specified as SE > − 6.0 dioptres (D) on at least one of the eyes. Slit lamp biomicroscopy with applantion tonometry and fundus ophthalmoscopy in mydriasis was carried out (Topcon SL-D701, Topcon, Tokyo, Japan). Digital fundus photography (TRC-501X; Topcon, Tokyo, Japan) and in some cases also ultra-wide field (200°) fundus images (Optos® California, Optos, Marlborough, MA) were taken. Spectral domain optical coherence tomography (macular scan) (Heidelberg Engineering, Heidelberg, Germany) was performed where possible. Axial length measurements were executed with an optical biometry system (IOLMaster 700, Carl Zeiss, Jena, Germany). Automated kinetic full-field perimetry was carried out with Humphrey Field Analyzer (Carl Zeiss Meditec, Jena, Germany).

### Electrophysiology

Pattern visual evoked potentials (VEPs), pattern-, standard full-field- and multifocal electroretinography (ERG) were carried out. All electrophysiology tests were performed according to the ISCEV standards [43–46] and using the Roland Electrophysiological Test Unit with the RETIport 32 software (Roland Consult, Brandenburg a.d. Havel, Germany). Please see the Additional file 3: Supplementary text for more details.

### Colour vision testing

Colour vision deficiencies were assessed using the Lanthony Desaturated D-15-hue Panel tests where possible and the Isihara pseudoisochromatic plates (Isihara 24 plates edition, 2006) in the rest of the cases.

## Supplementary Information

**Additional file 1.** **Figure S1.** The cone hypothesis. **Figures S2–S21.** Fundus images, macular OCTs, RNFLs and visual fields of patients III/8, IV/1, IV/2, IV/6, IV/7 and IV/10.

**Additional file 2.** **Figures S22–S55.** Standard full field ERG, PERG, pVEP and mfERG of patients III/3, III/8, IV/1, IV/2, IV/6 and IV/10. (Not every patient went through the full list of electrophysiology analyses.)

**Additional file 3.** Ophthalmology findings. Electrophysiology methods. Electrophysiology findings (Pattern VEP, Pattern ERG, Standard full field ERGs, Multifocal ERGs). Colour vision testing. Numerical electrophysiology data: Table S1, Table S2, Table S3.

## Data Availability

The sequencing data used and analysed during the current study are available from the corresponding author on reasonable request. All other data generated or analysed during this study are included in this published article and its supplementary information files.

## Abbreviations

BCVA: Best corrected visual acuity
eoHM: Early onset high myopia
ERG: Electroretinography
IOP: Intraocular pressure
GPCR: G-protein coupled receptor
ipRGC: Intrinsically photosensitive retinal ganglion cell
mfERG: Multifocal electroretinography
OCT: Optical coherence tomography
ONH: Optic nerve head
PERG: Pattern electroretinography
POAG: Primary open angle glaucoma
RGC: Retinal ganglion cell
SE: Spherical equivalent
VA: Visual acuity
VEP: Visual evoked potentials
WES: Whole exome sequencing
