# Supplementary material for: Myopia-26, the female-limited form of early-onset high myopia, occurring in a European family
Source: Orphanet J Rare Dis. 2021 Jan 22;16:45. doi: 10.1186/s13023-021-01673-z (PMC7825233; doi:10.1186/s13023-021-01673-z)
Supplement: Supplementary file 1 — Additional file 1. Figure S1. The cone hypothesis. Figures S2–S21. Fundus images, macular OCTs, RNFLs and visual fields of patients III/8, IV/1, IV/2, IV/6, IV/7 and IV/10. [file 13023_2021_1673_MOESM1_ESM.pdf]

SUPPLEMENTARY FIGURES I.

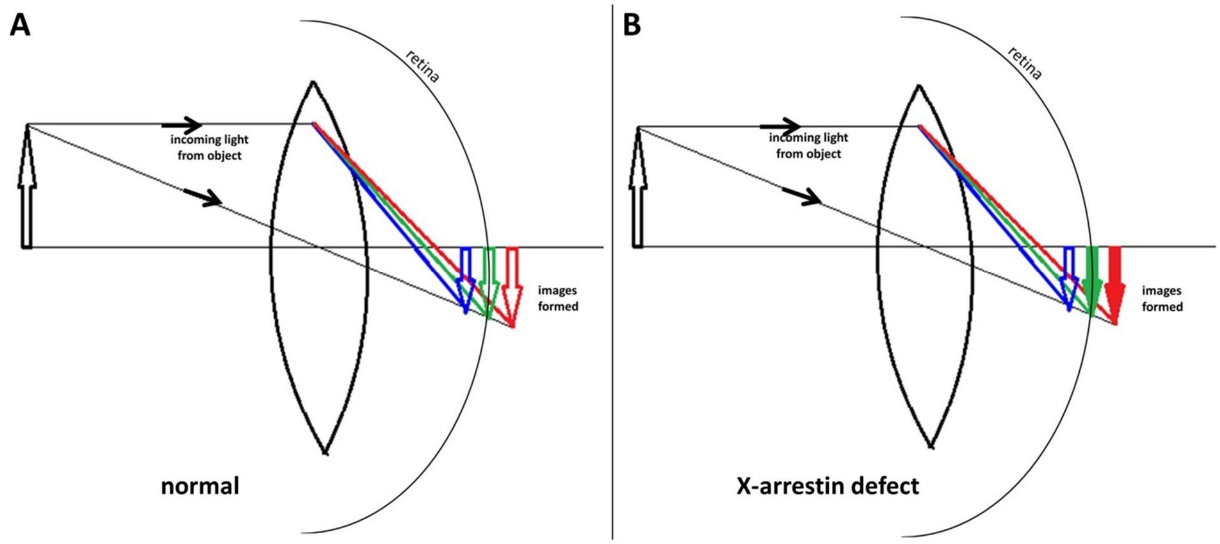

Figure S1. The cone hypothesis. A: Chromatic aberration results in short wavelengths forming an image anterior, long wavelengths forming an image posterior to the plane of the retina. B: In case of X-arrestin defect, LM cones are more active due to the lack of LM cone desensitisation. As a result, the images formed posterior to the retina give a more intensive signal, which is equivalent to a hyperopic defocus. A constant hyperopic defocus leads to eye elongation in animal models.

Patient III/8

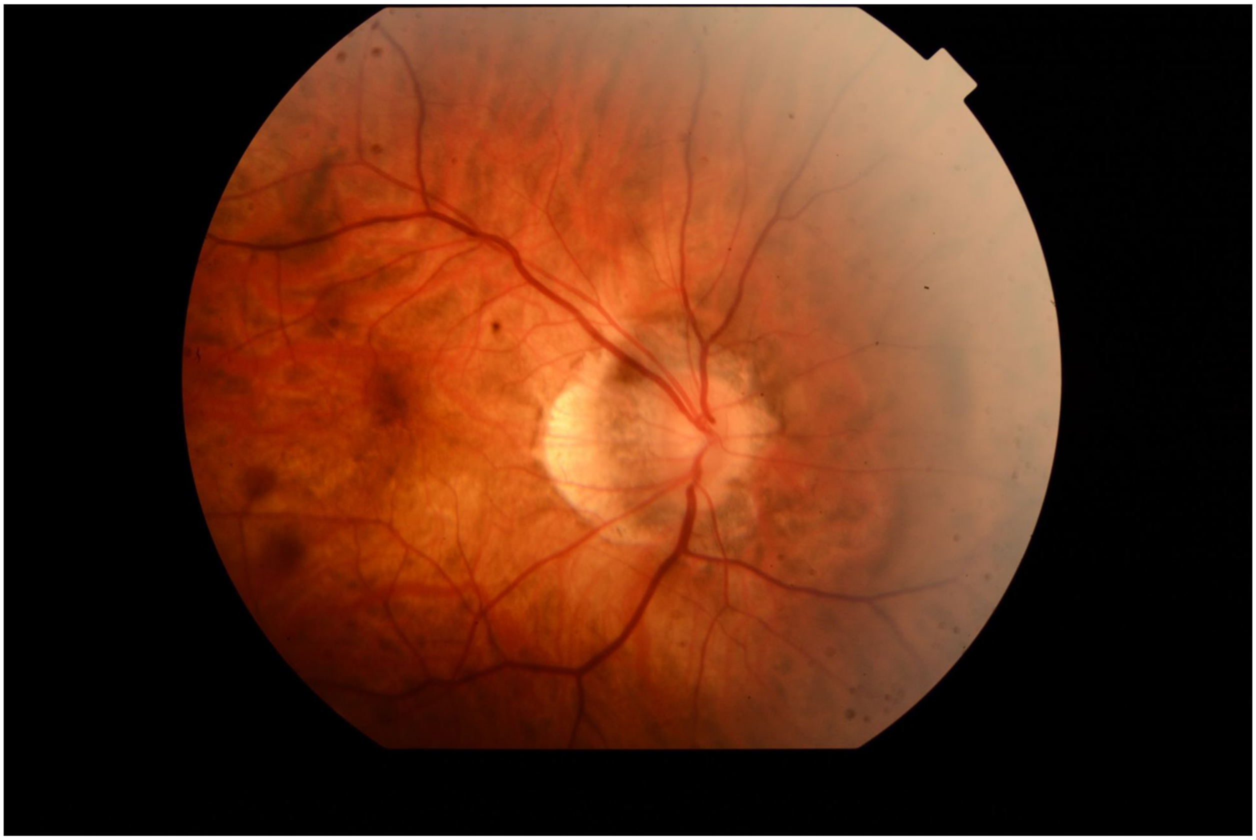

Figure S2. FLAG fundus image of right eye of patient III/8.

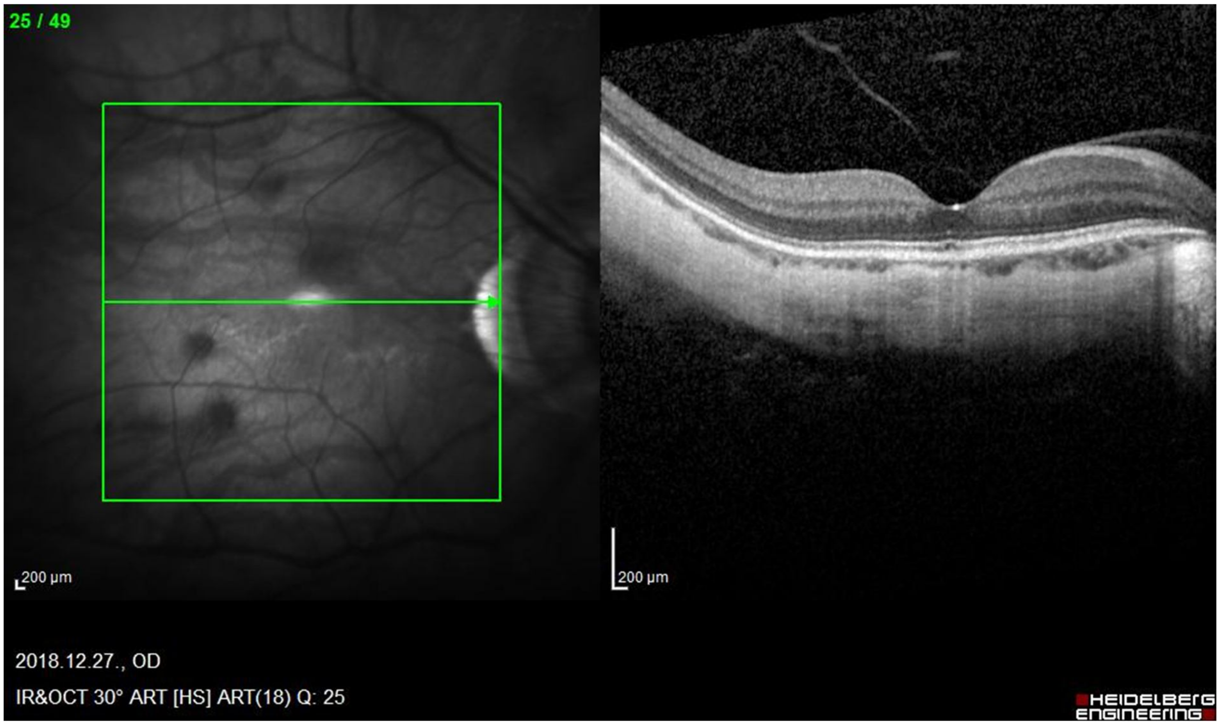

Figure S3. OCT image of macula, right eye of patient III/8.

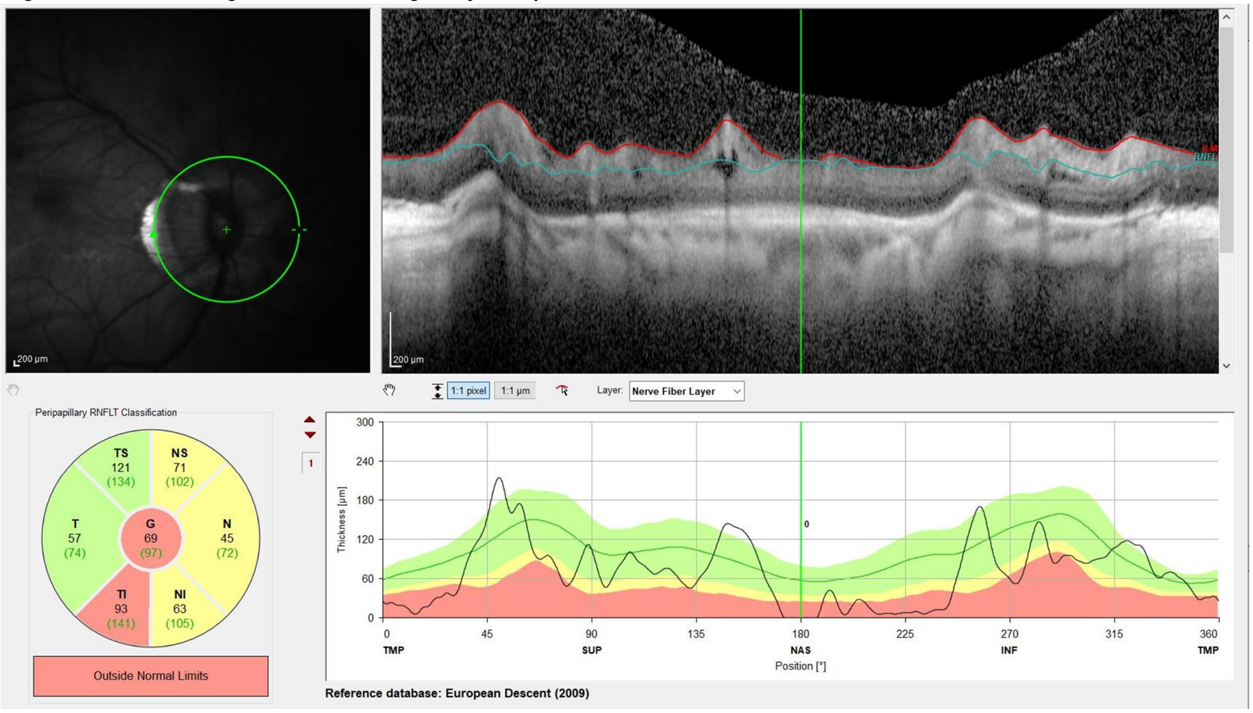

Figure S4. OCT (top) and RNFL image (bottom) of right eye of patient III/8.

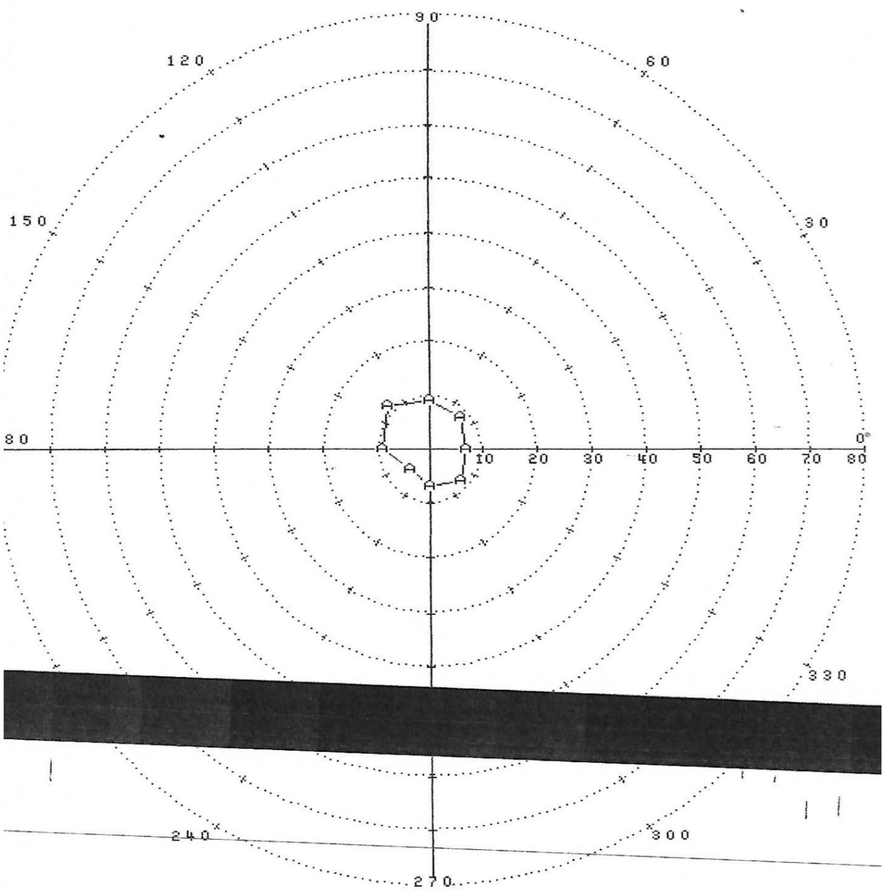

Figure S5. Visual field of the right eye of patient III/8.

Patient IV/1

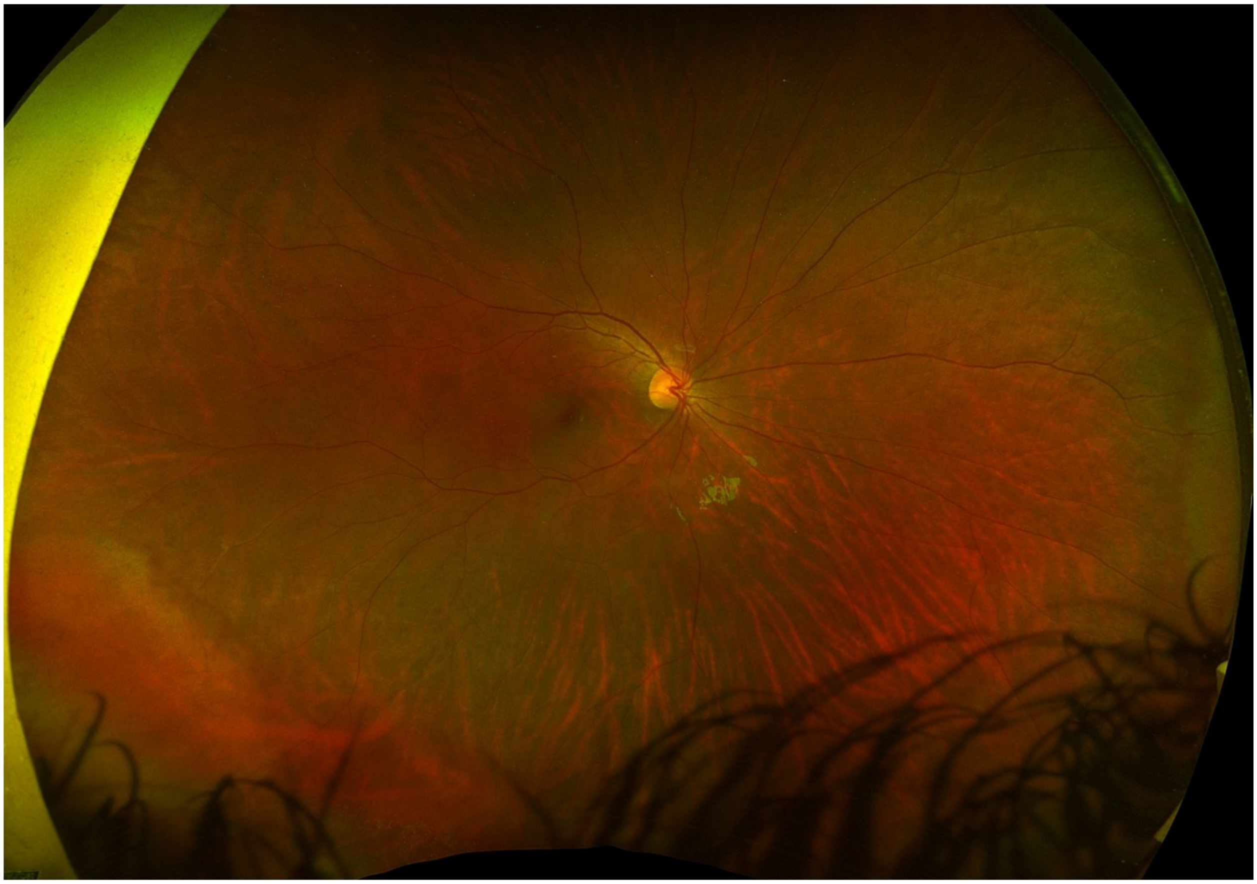

Figure S6. OPTOS fundus image of the right eye of patient IV/1.

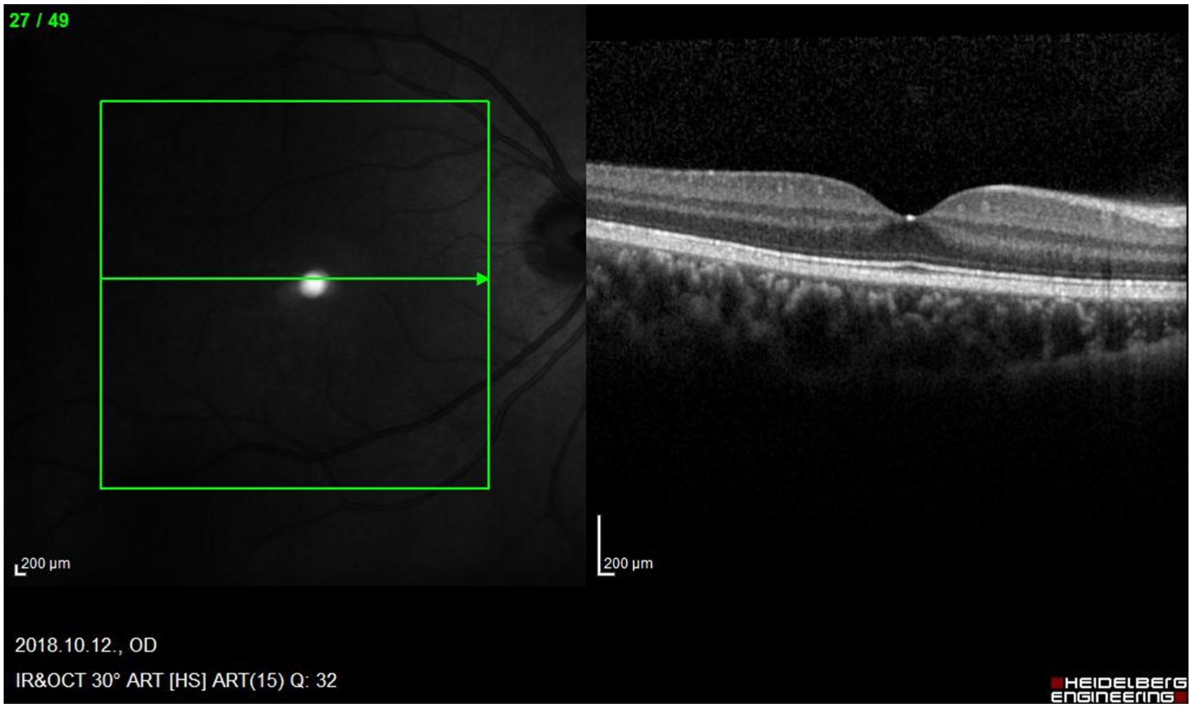

Figure S7. OCT image of macula, right eye of patient IV/1.

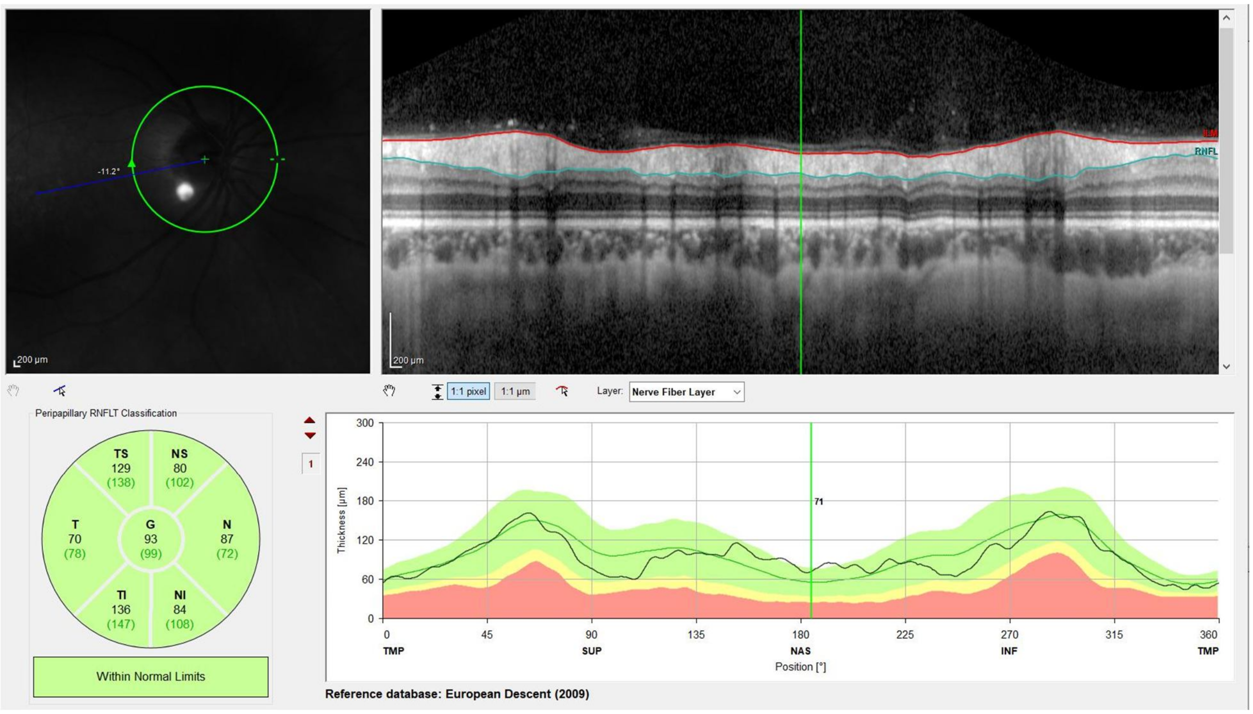

Figure S8. OCT (top) and RNFL image (bottom) of the right eye of patient IV/1.

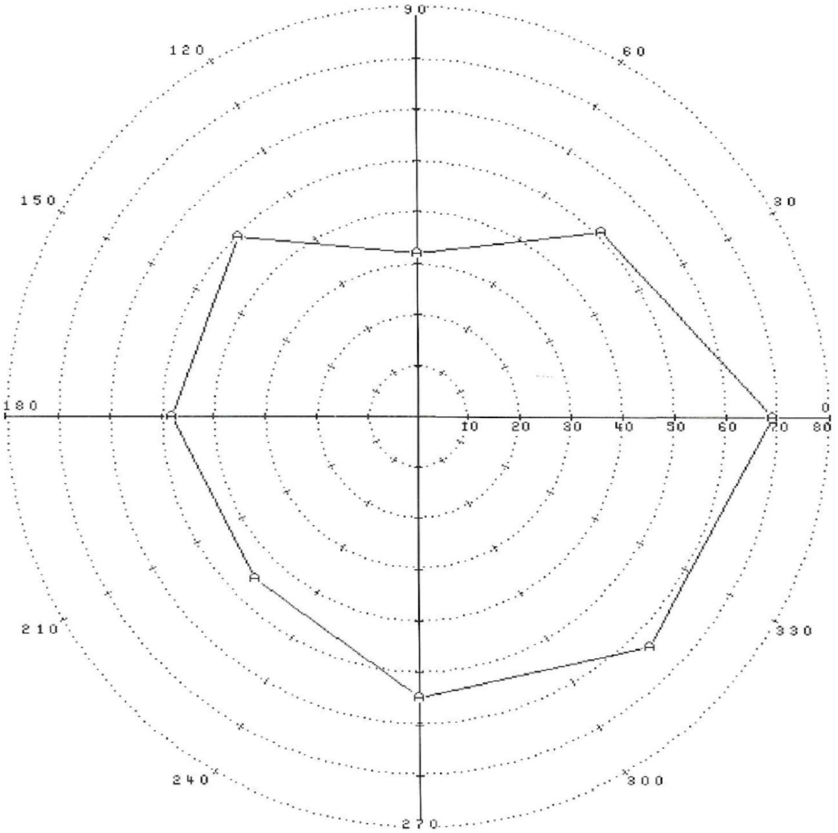

Figure S9. Visual field of the right eye of patient IV/1.

Patient IV/2

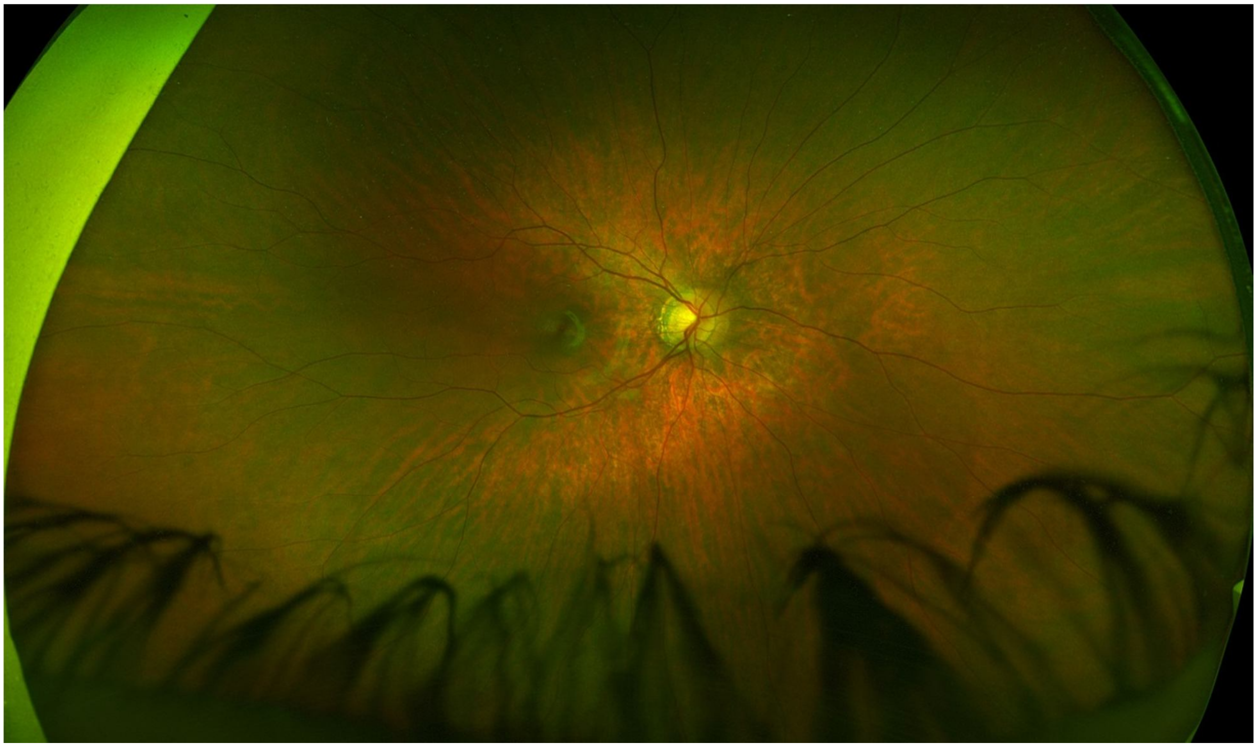

Figure S10. OPTOS fundus image of the right eye of patient IV/2.

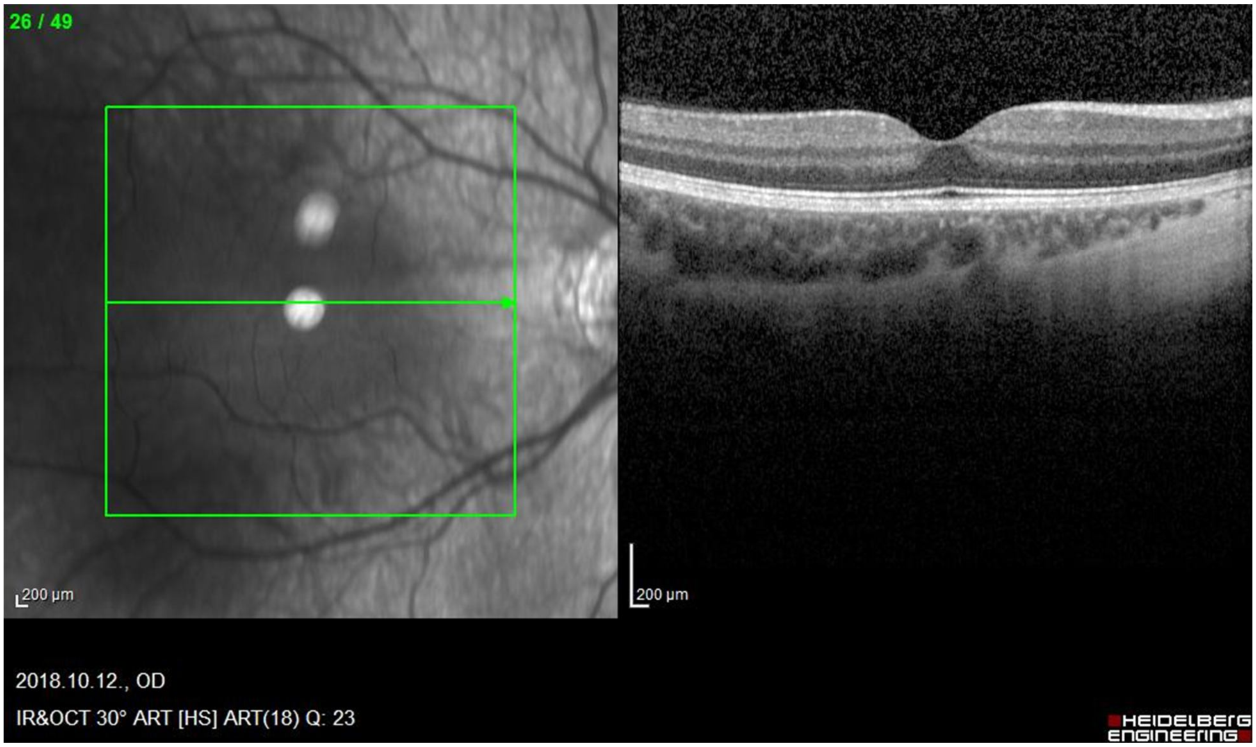

Figure S11. OCT image of macula, right eye of patient IV/2.

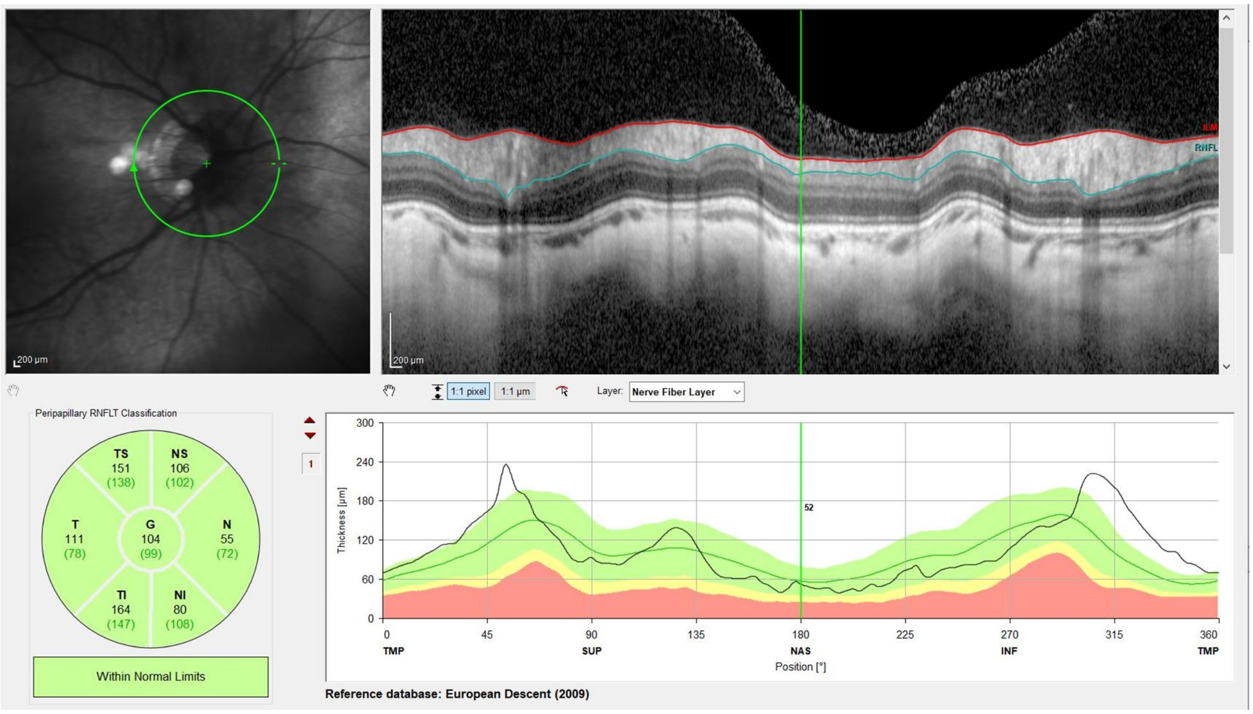

Figure S12. OCT (top) and RNFL image (bottom) of the right eye of patient IV/2.

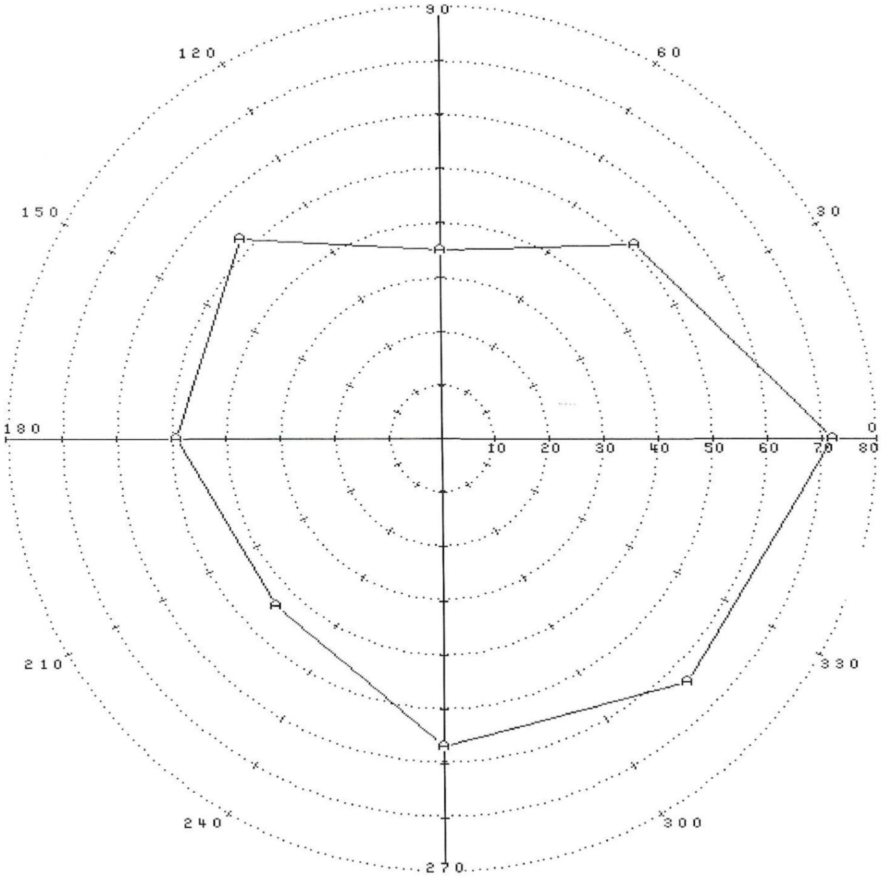

Figure S13. Visual field of the right eye of patient IV/2.

Patient IV/6

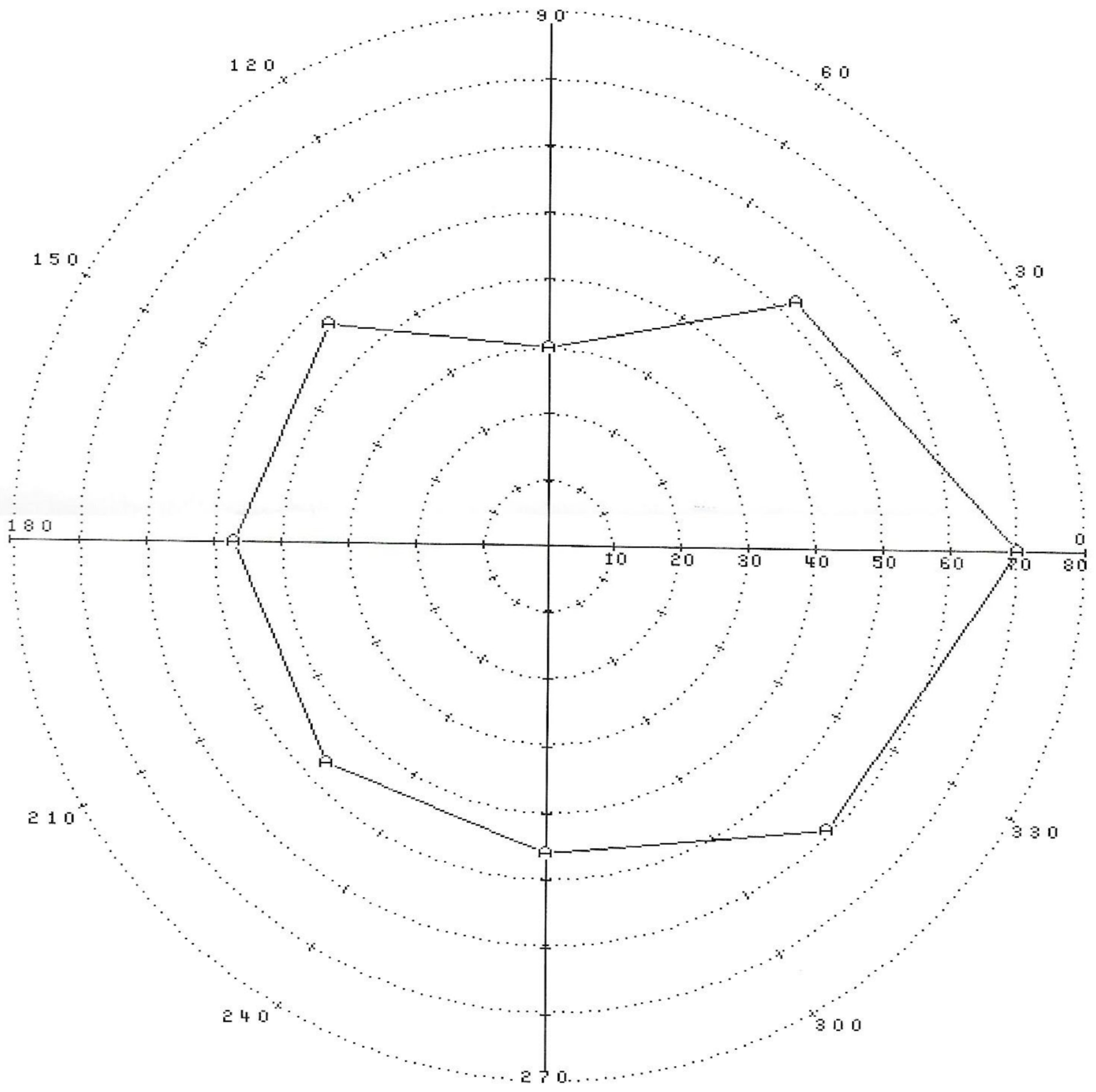

Figure S14. Visual field of the right eye of patient IV/6.

Patient IV/7

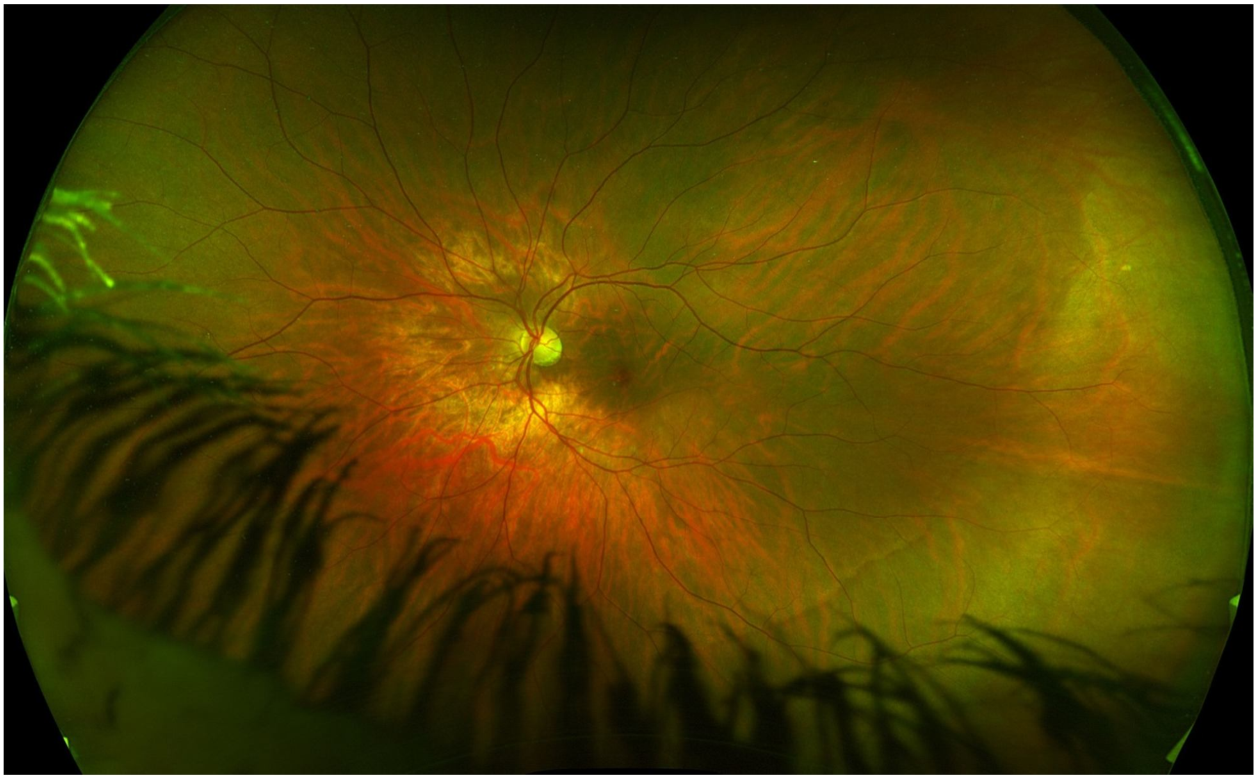

Figure S15. OPTOS fundus image of the left eye of patient IV/7.

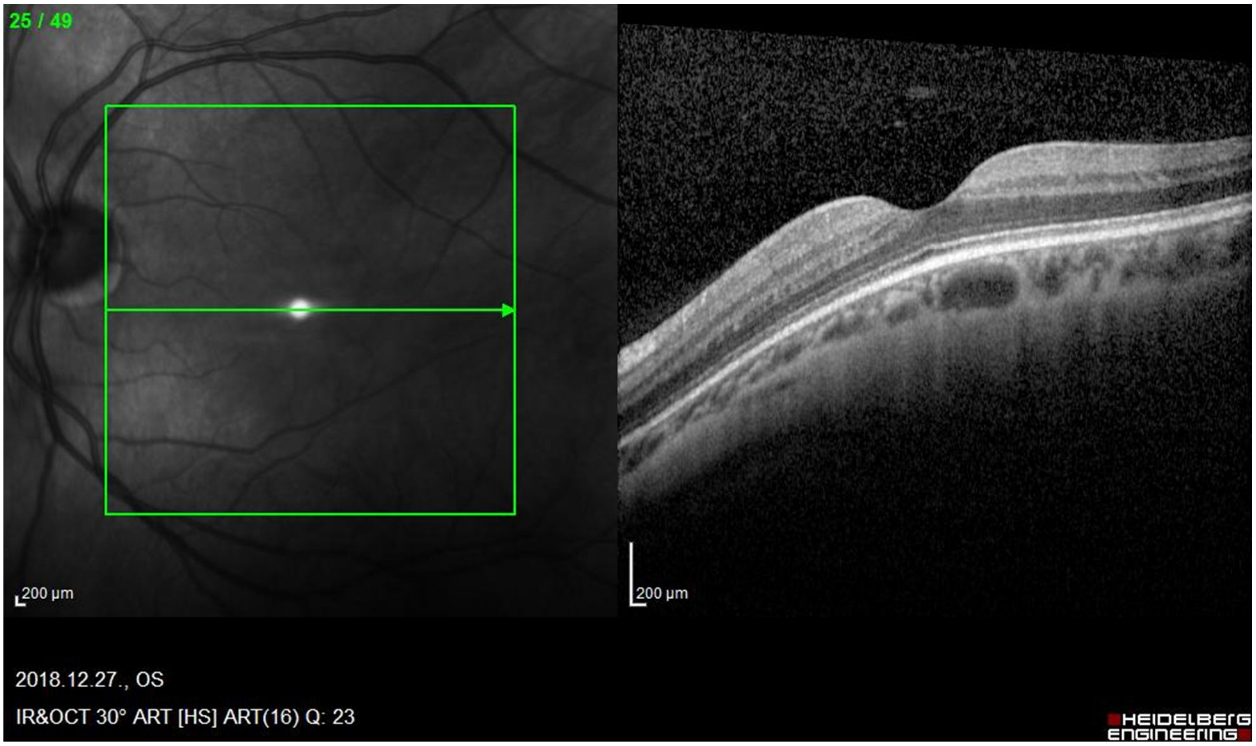

Figure S16. OCT image of macula, left eye of patient IV/7.

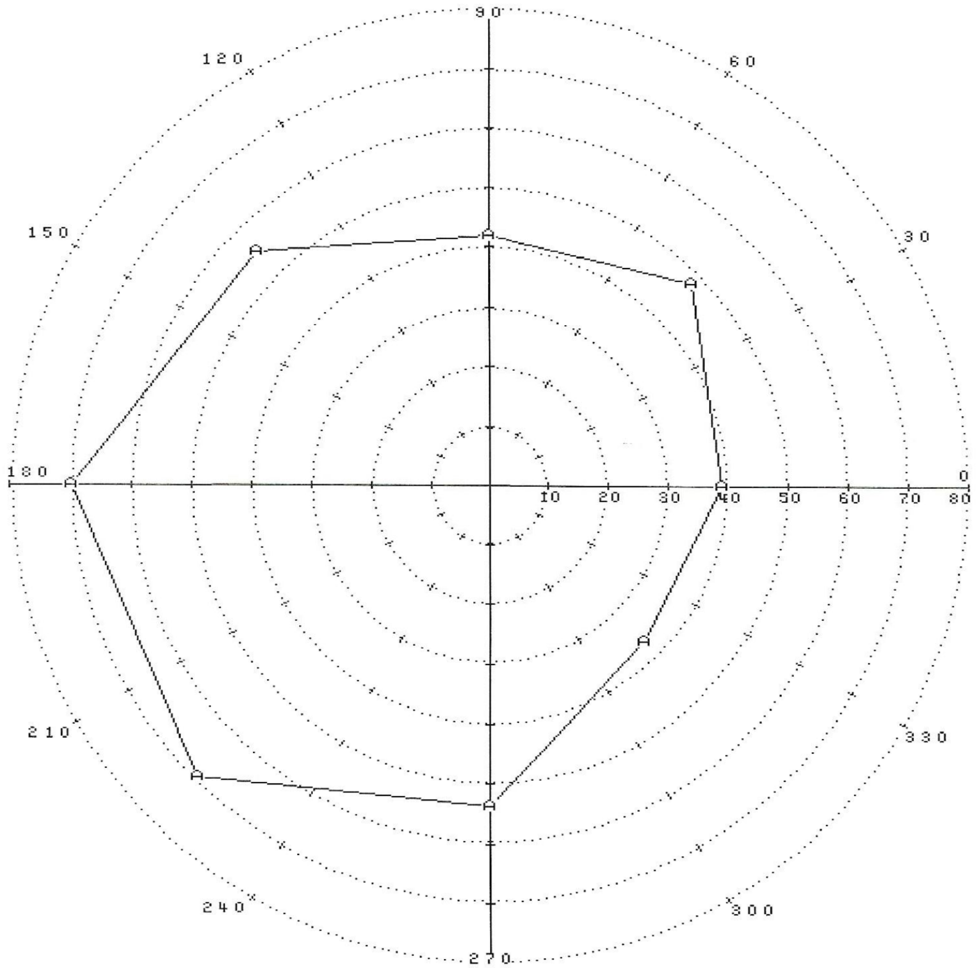

Figure S17. Visual field of the left eye of patient IV/7.

Patient IV/10

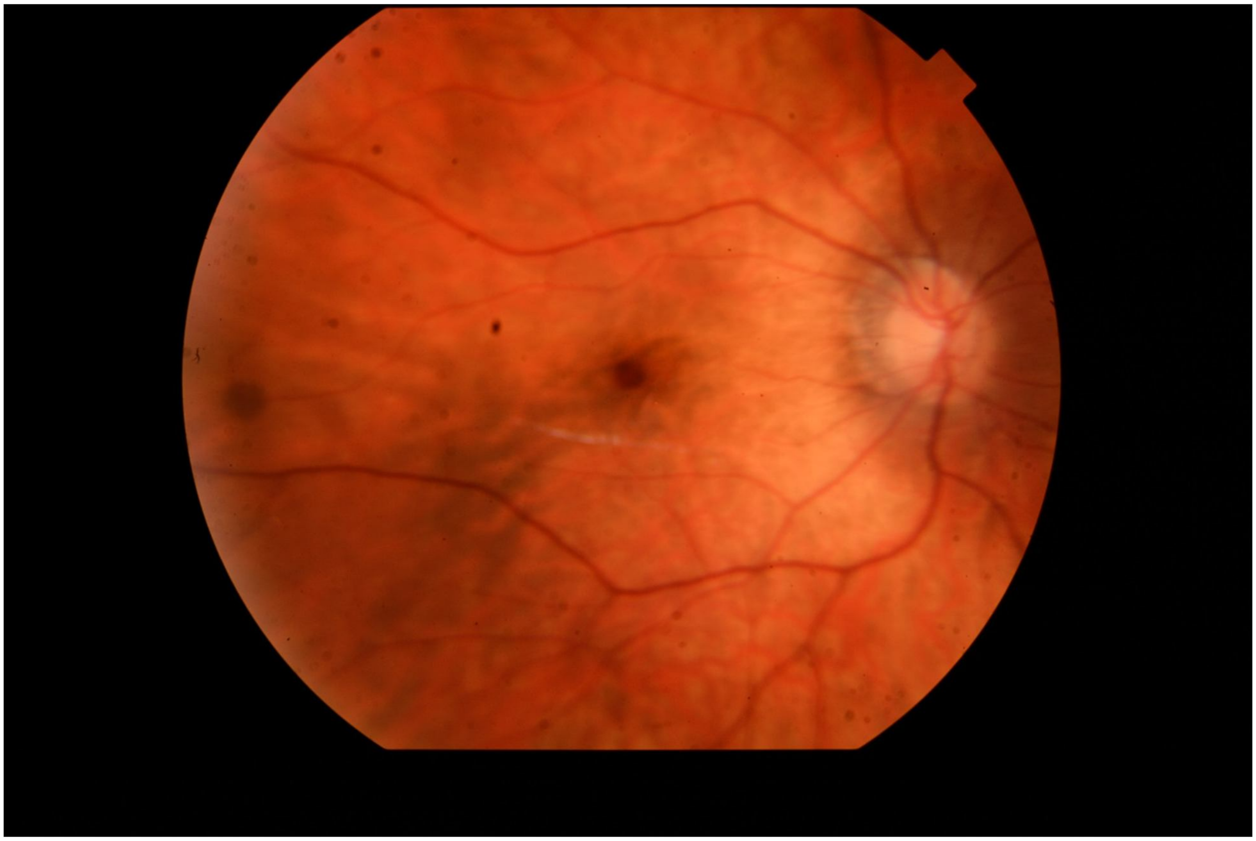

Figure S18. FLAG fundus image of the right eye of patient IV/10.

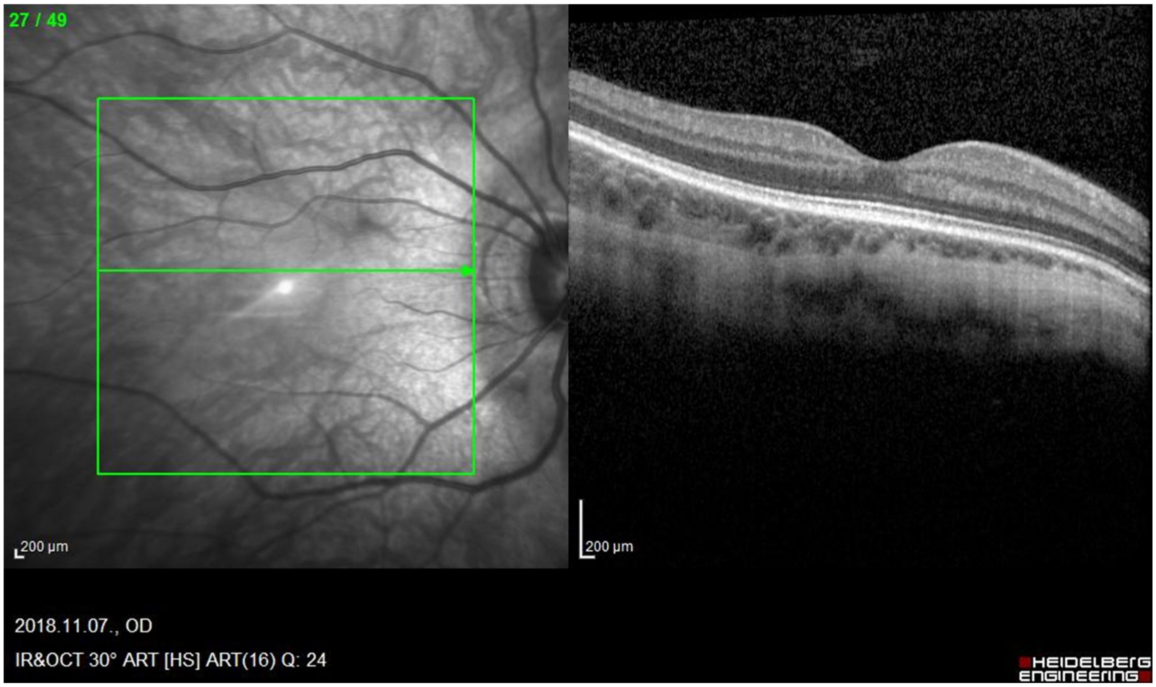

Figure S19. OCT image of macula, right eye of patient IV/10.

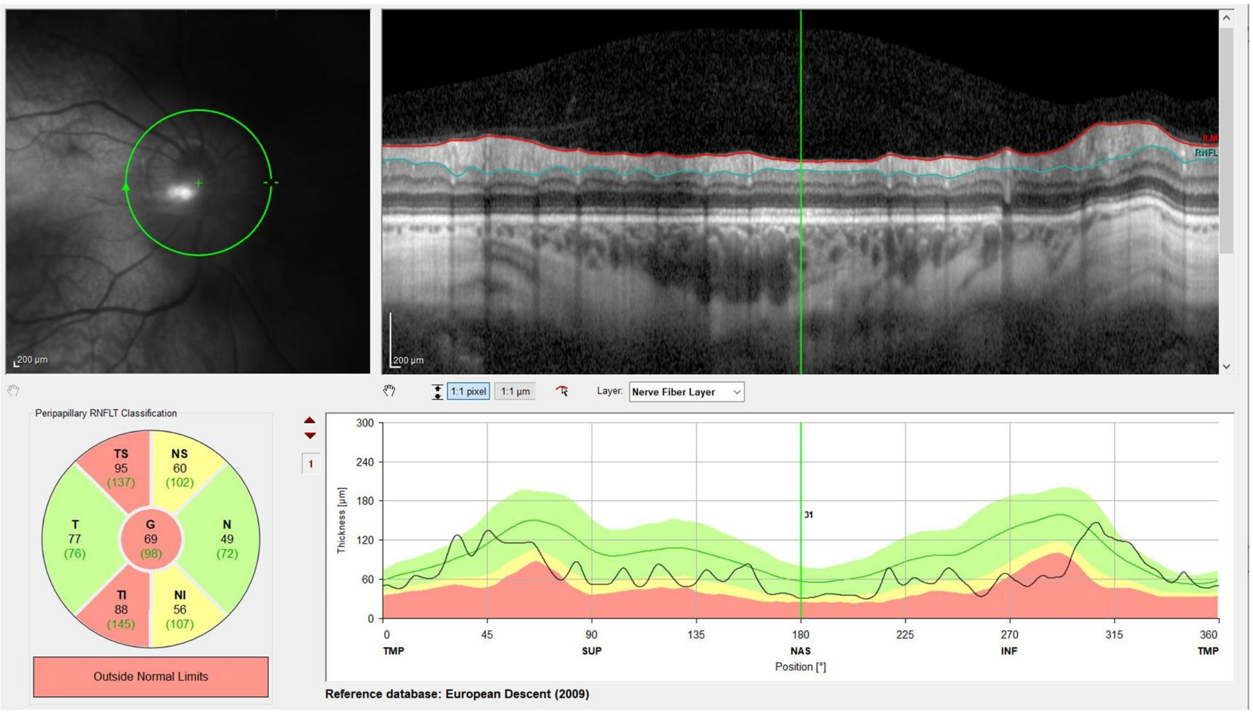

Figure S20. OCT (top) and RNFL image (bottom) of the right eye of patient IV/10.

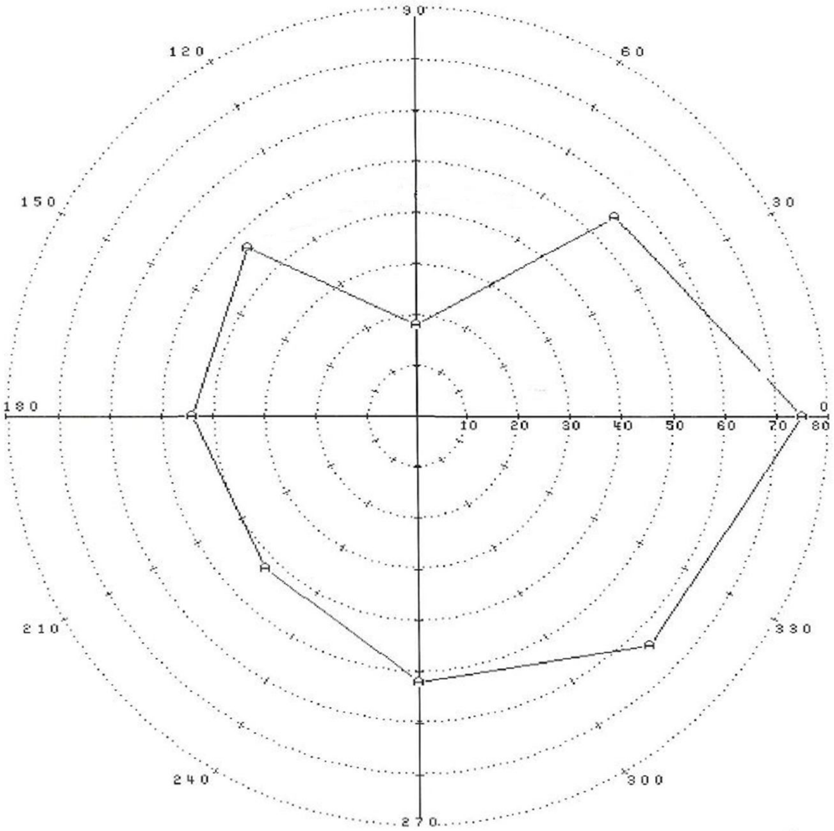

Figure S21. Visual field of the right eye of patient IV/10.
