# Supplementary material for: Myopia-26, the female-limited form of early-onset high myopia, occurring in a European family
Source: Orphanet J Rare Dis. 2021 Jan 22;16:45. doi: 10.1186/s13023-021-01673-z (PMC7825233; doi:10.1186/s13023-021-01673-z)
Supplement: Supplementary file 2 — Additional file 2. Figures S22–S55. Standard full field ERG, PERG, pVEP and mfERG of patients III/3, III/8, IV/1, IV/2, IV/6 and IV/10. (Not every patient went through the full list of electrophysiology analyses.) [file 13023_2021_1673_MOESM2_ESM.pdf]

SUPPLEMENTARY FIGURES II.
Electrophysiology

photopic 3.0

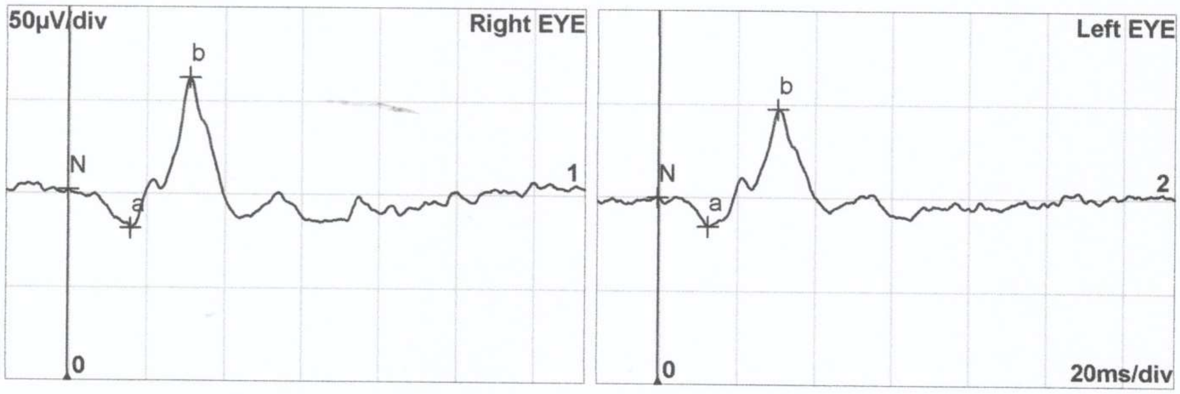

Channel	a [ms]	b [ms]	a-wave	b-wave
1 R-1	16	31	20,6µV	81µV
2 L-2	13	31	15,5µV	63,2µV
Normals	-	-		

30Hz Flicker 3.0

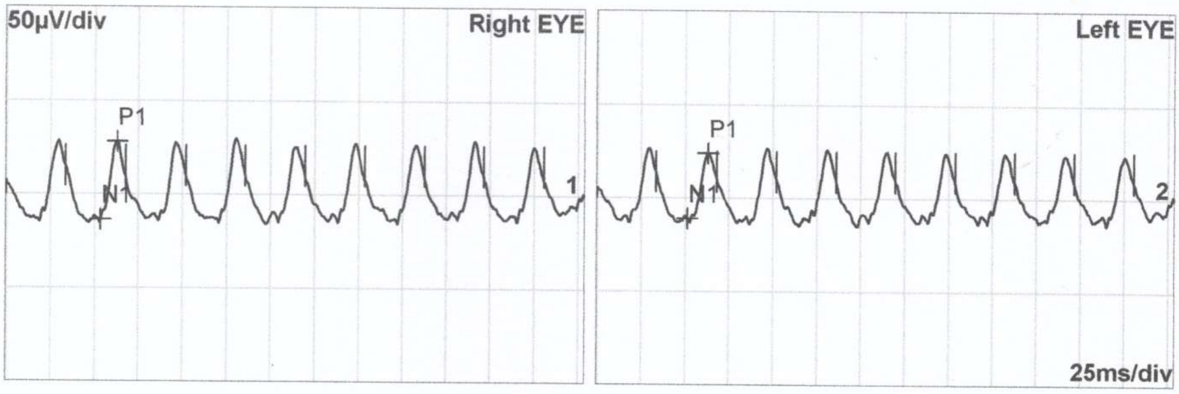

Channel	N1 [ms]	P1 [ms]	N1-P1
1 R-1	54	64	41,5µV
2 L-2	52	64	35µV

Figure S22. Standard full-field ERG recording of patient III/3.

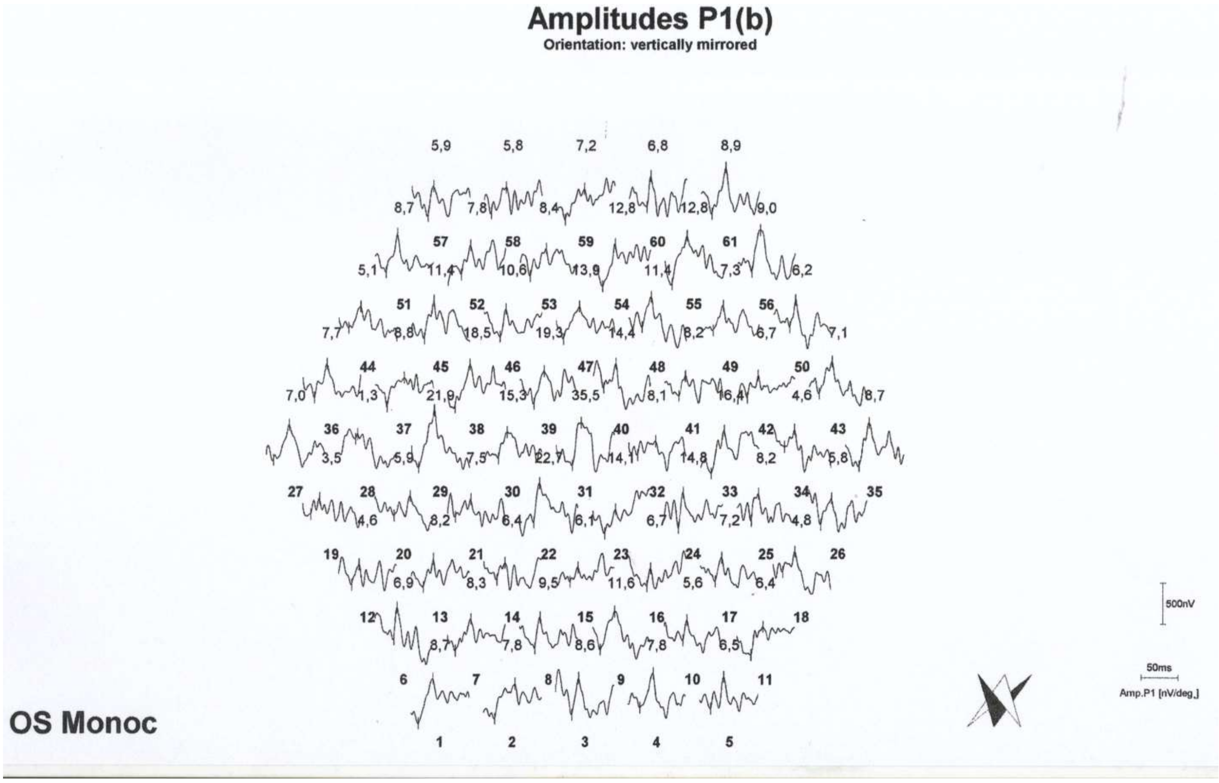

Figure S23. mfERG recording of patient III/3, OS.

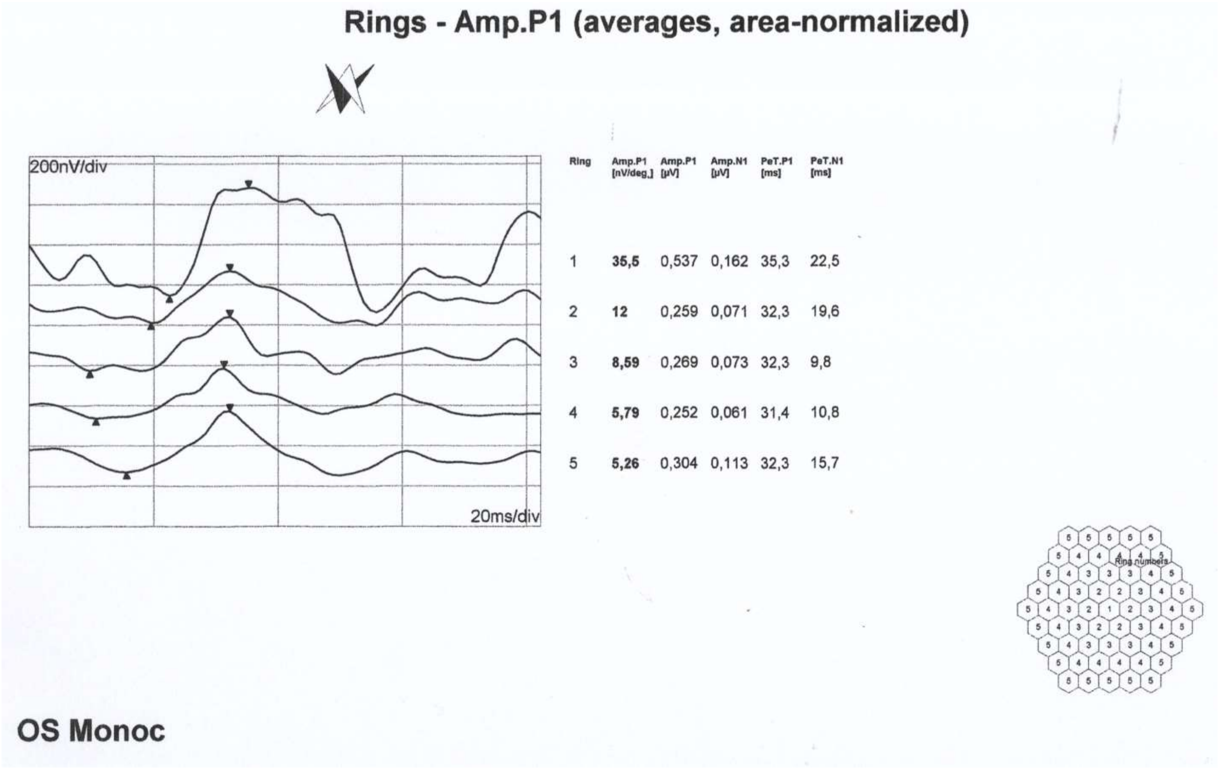

Ring	Amp.P1 [nV/deg.]	Amp.P1 [µV]	Amp.N1 [µV]	PeT.P1 [ms]	PeT.N1 [ms]
1	**35,5**	0,537	0,162	35,3	22,5
2	**12**	0,259	0,071	32,3	19,6
3	**8,59**	0,269	0,073	32,3	9,8
4	**5,79**	0,252	0,061	31,4	10,8
5	**5,26**	0,304	0,113	32,3	15,7

Figure S24. mfERG ring analysis of patient III/3, OS.

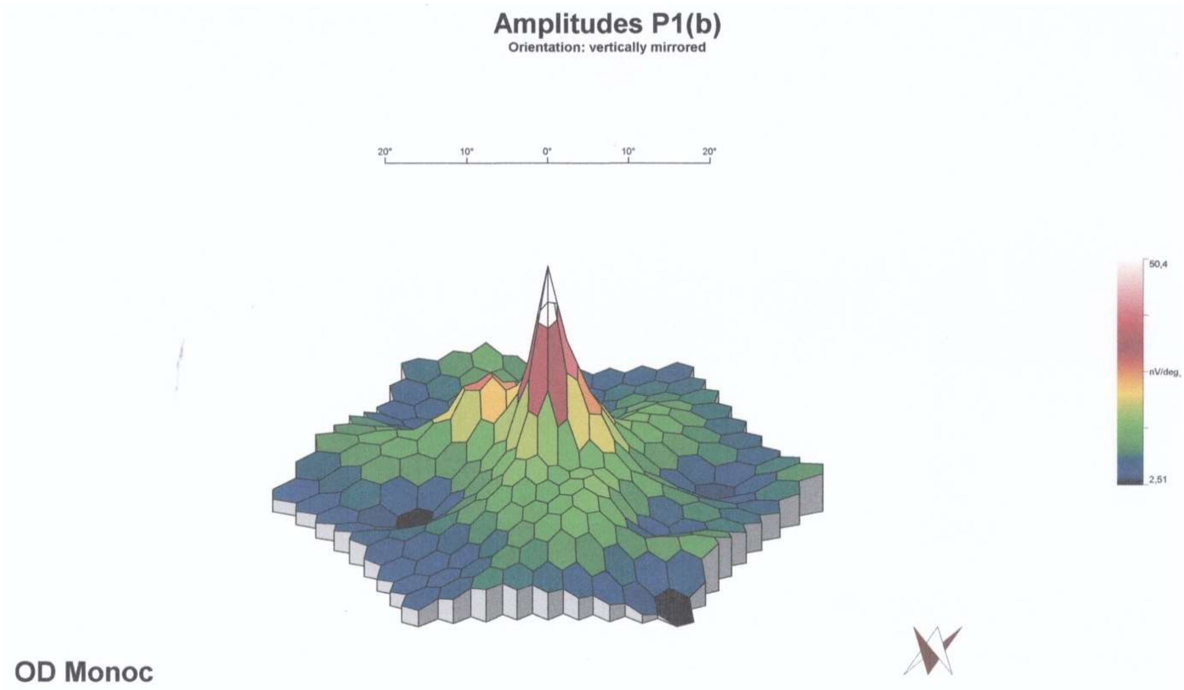

Figure S25. Multifocal ERG 3 D colour map of III/3 carrier male right eye displays overall normal central macular function with scarce subnormal responses only.

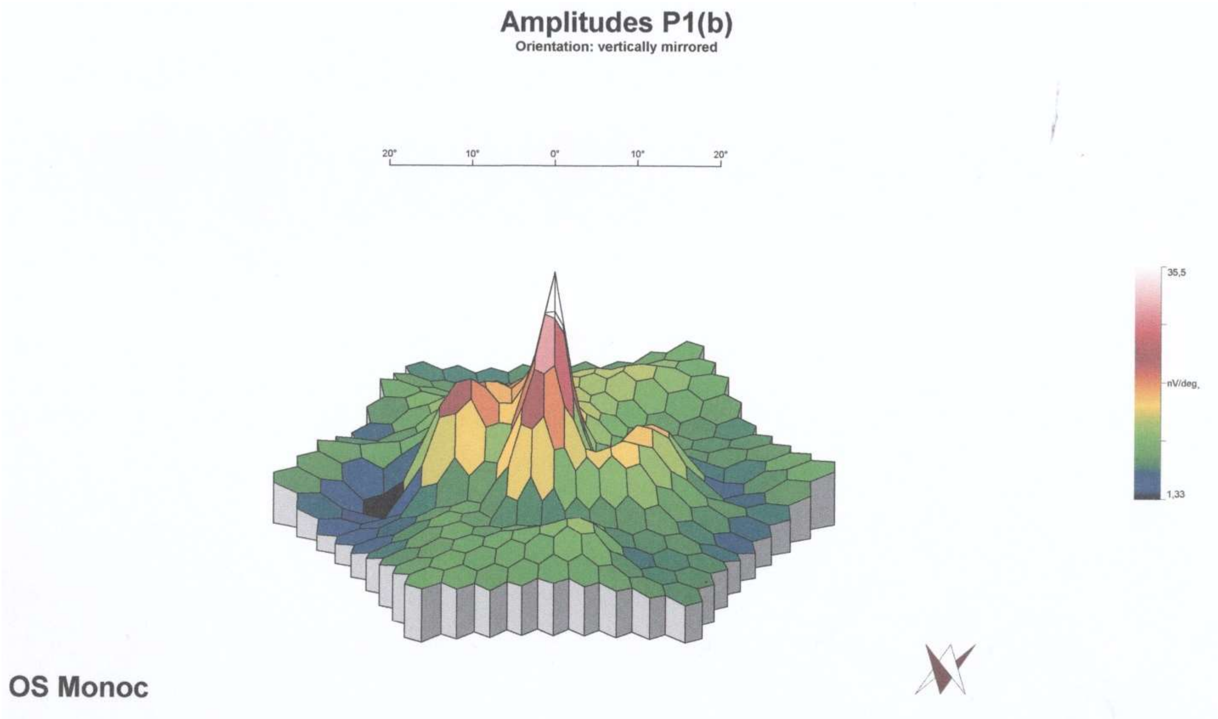

Figure S26. Multifocal ERG 3 D colour map of III/3 carrier male, left eye displays overall normal central macular function with scarce subnormal responses only.

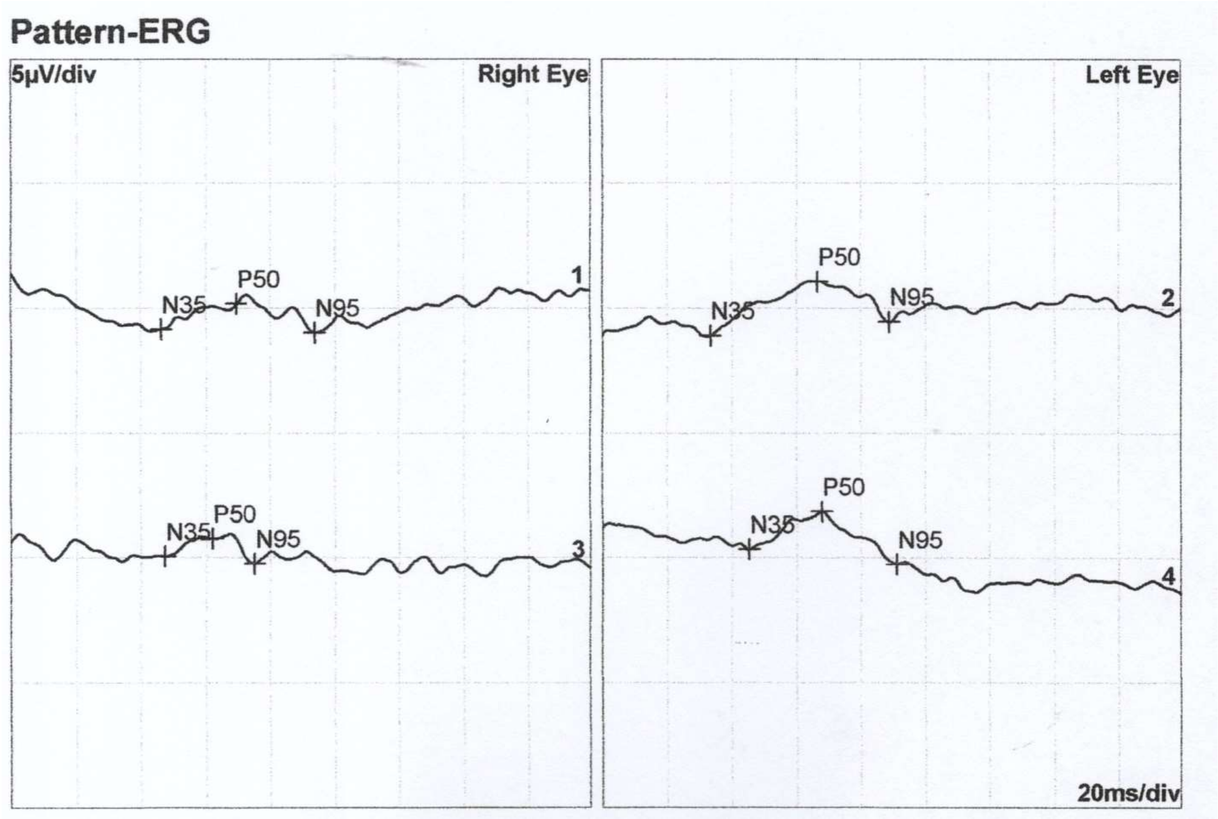

Figure S27. PERG recording of patient III/8. Lines 1 and 3 and lines 2 and 4 represent pairs of replicate measurements.

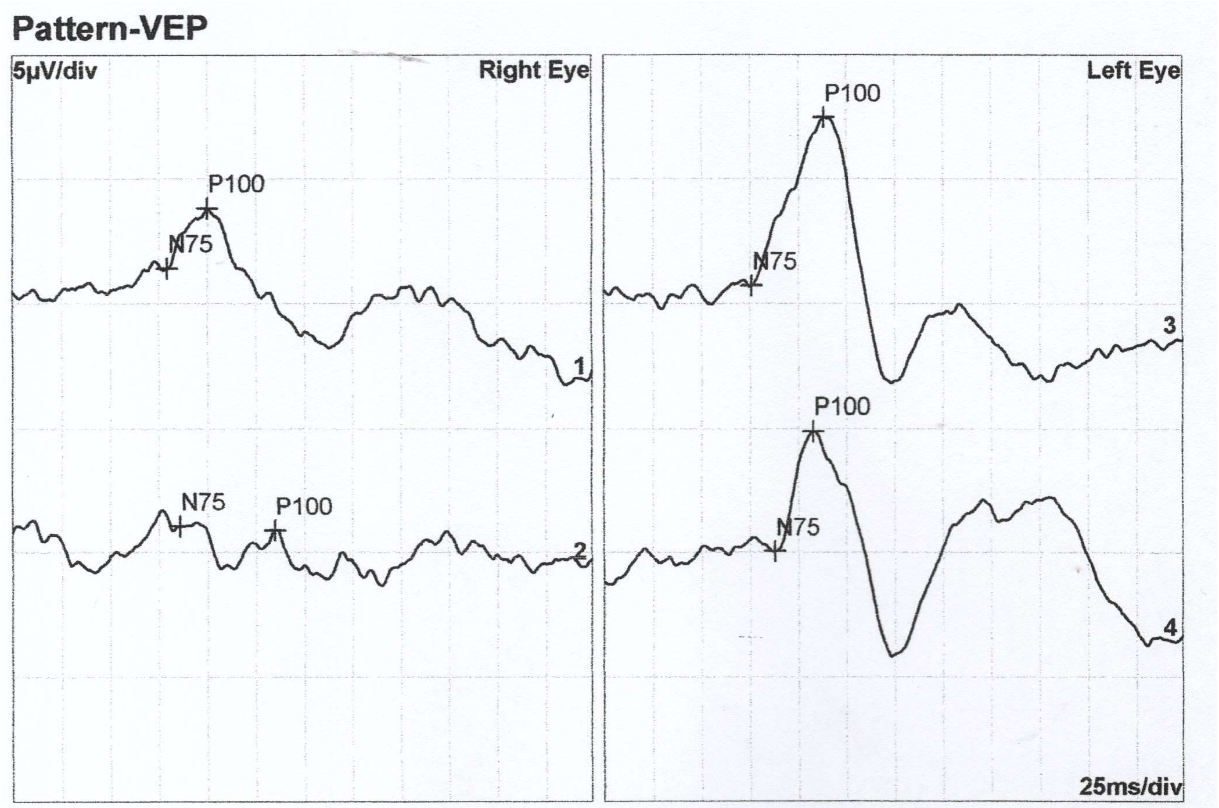

Figure S28. pVEP recording of patient III/8. Lines 1 and 3 display responses to 60' stimuli and lines 2 and 4 represent responses to 15' stimuli.

photopic 3.0

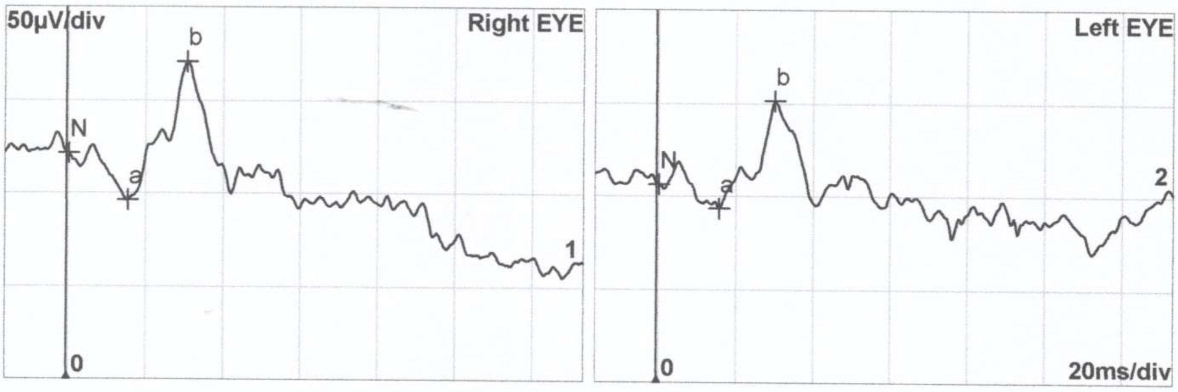

Channel	a [ms]	b [ms]	a-wave	b-wave
1 R-1	16	31	25,6µV	74,5µV
2 L-2	16	31	13µV	58,1µV
Normals	-	-		

30Hz Flicker 3.0

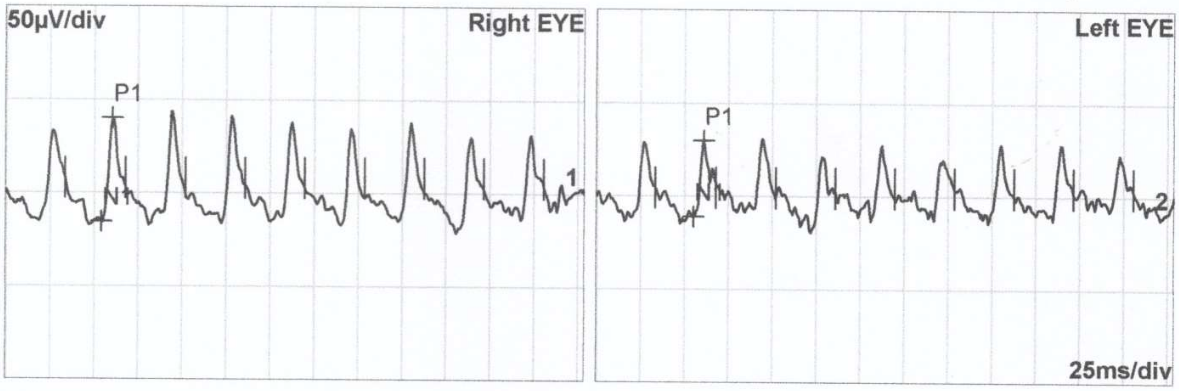

Channel	N1 [ms]	P1 [ms]	N1-P1
1 R-1	55	61	55,6µV
2 L-2	56	62	40,9µV

Figure S29. Standard full-field ERG recording of patient IV/1.

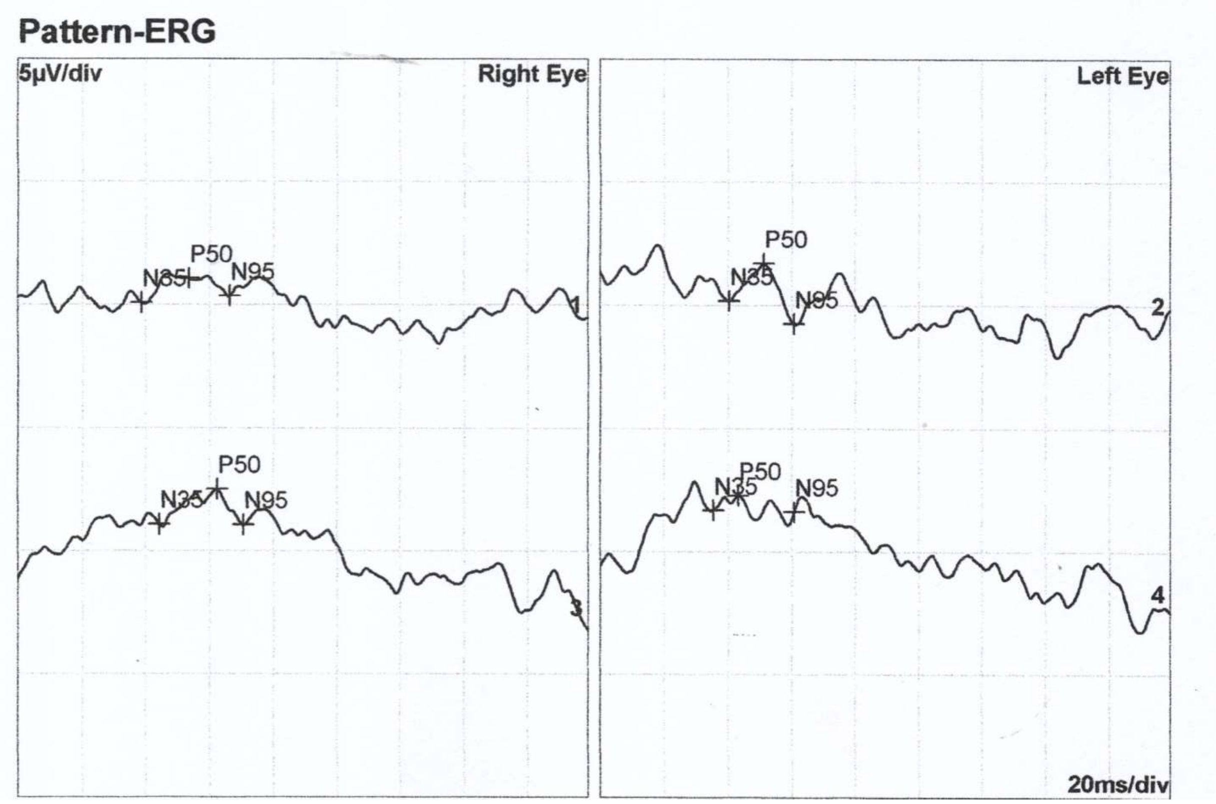

Figure S30. PERG recording of patient IV/1. Lines 1 and 3 and lines 2 and 4 represent pairs of replicate measurements.

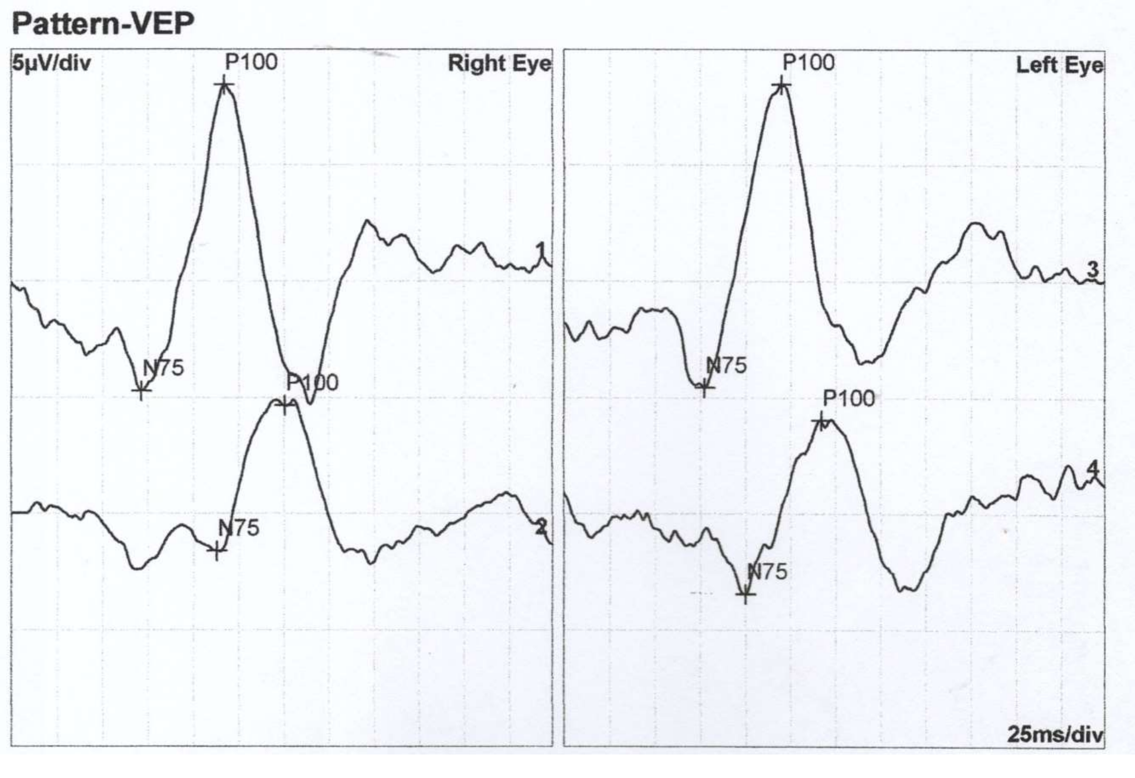

Figure S31. pVEP recording of patient IV/1. Lines 1 and 3 display responses to 60' stimuli and lines 2 and 4 represent responses to 15' stimuli.

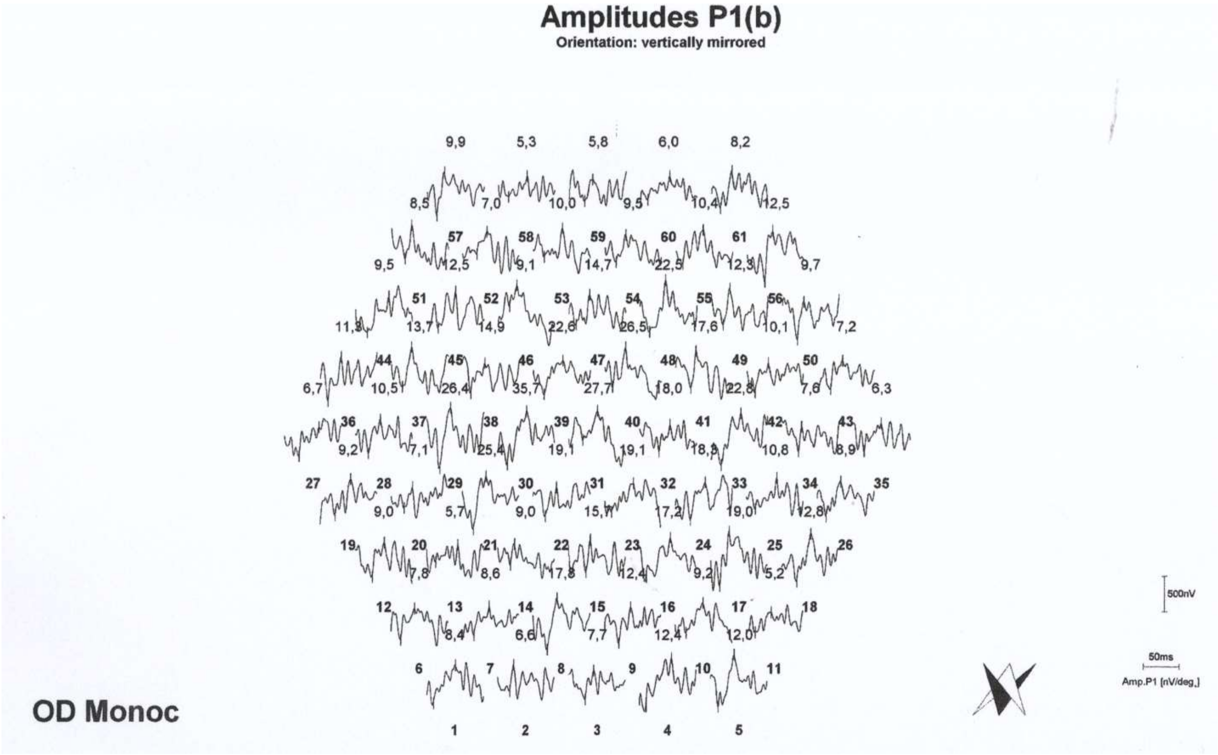

Figure S32. mfERG recording of patient IV/1, OD.

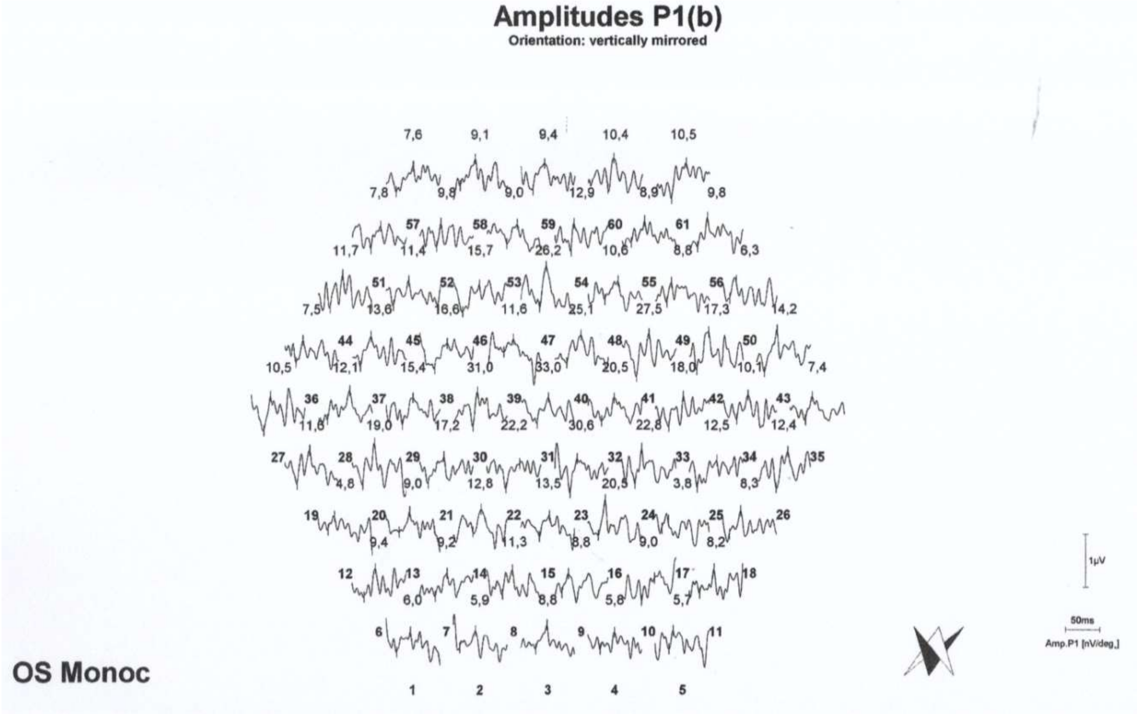

Figure S33. mfERG recording of patient IV/1,, OS.

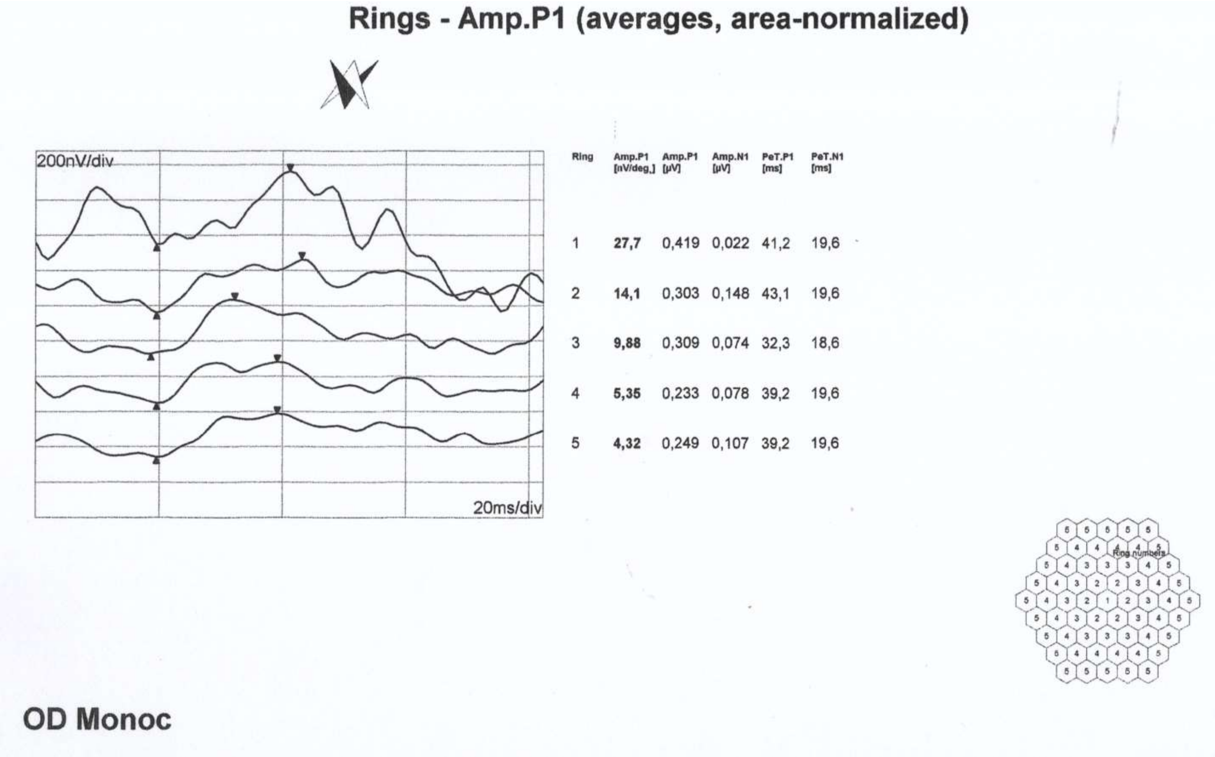

Figure S34. mfERG ring analysis of patient IV/1, OD.

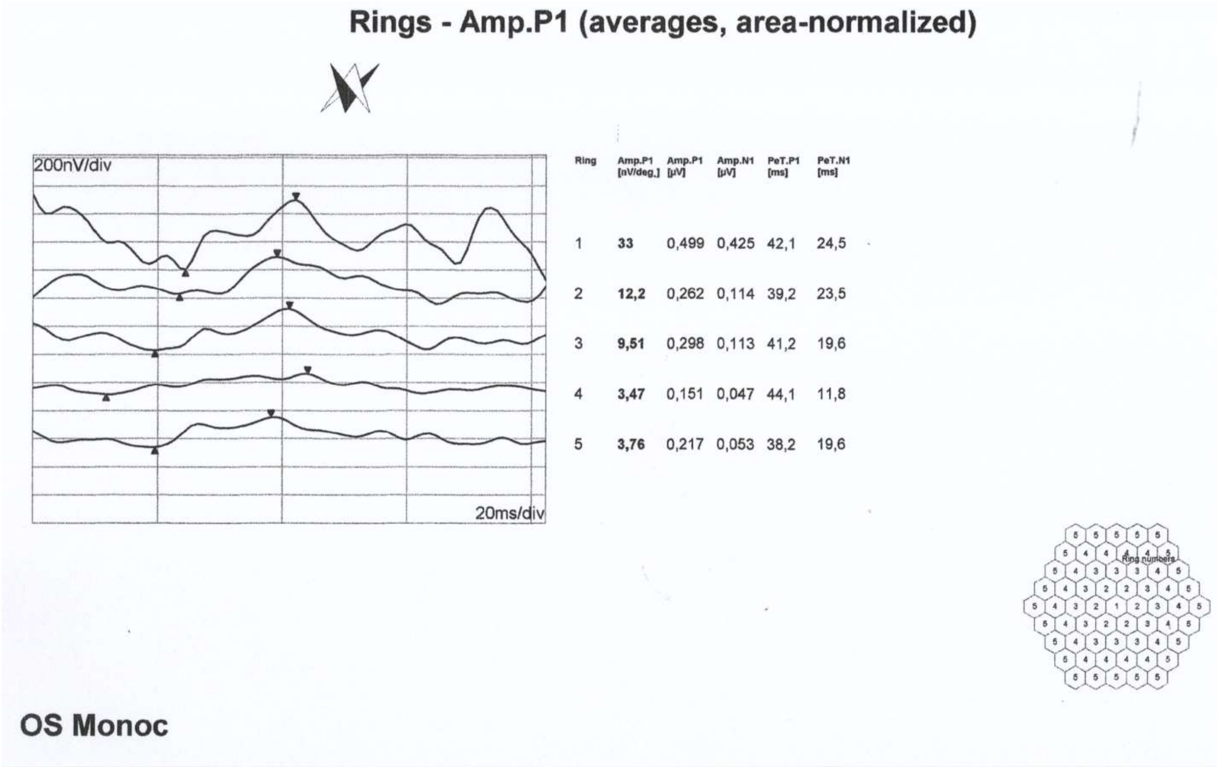

Ring	Amp.P1 [nV/deg.]	Amp.P1 [µV]	Amp.N1 [µV]	PeT.P1 [ms]	PeT.N1 [ms]
1	**33**	0,499	0,425	42,1	24,5
2	**12,2**	0,262	0,114	39,2	23,5
3	**9,51**	0,298	0,113	41,2	19,6
4	**3,47**	0,151	0,047	44,1	11,8
5	**3,76**	0,217	0,053	38,2	19,6

Figure S35. mfERG ring analysis of patient IV/1, OS.

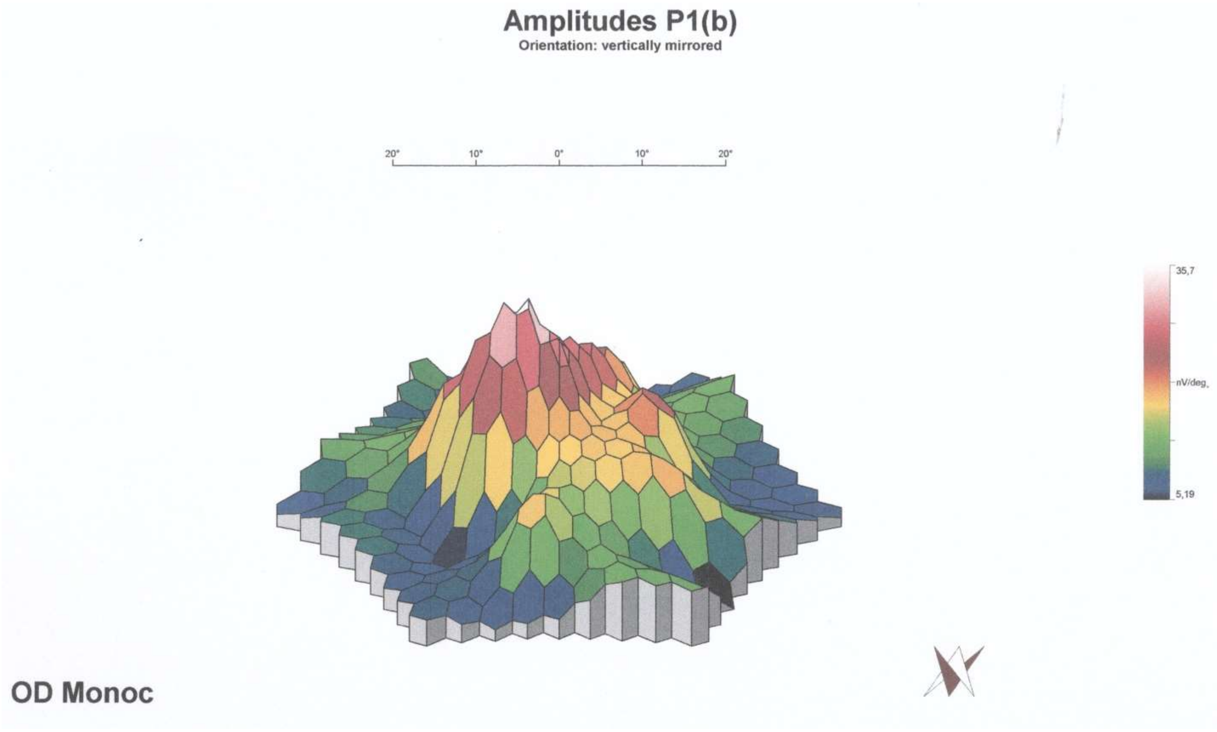

Figure S36. Multifocal ERG 3 D colour map of patient IV/1, OD

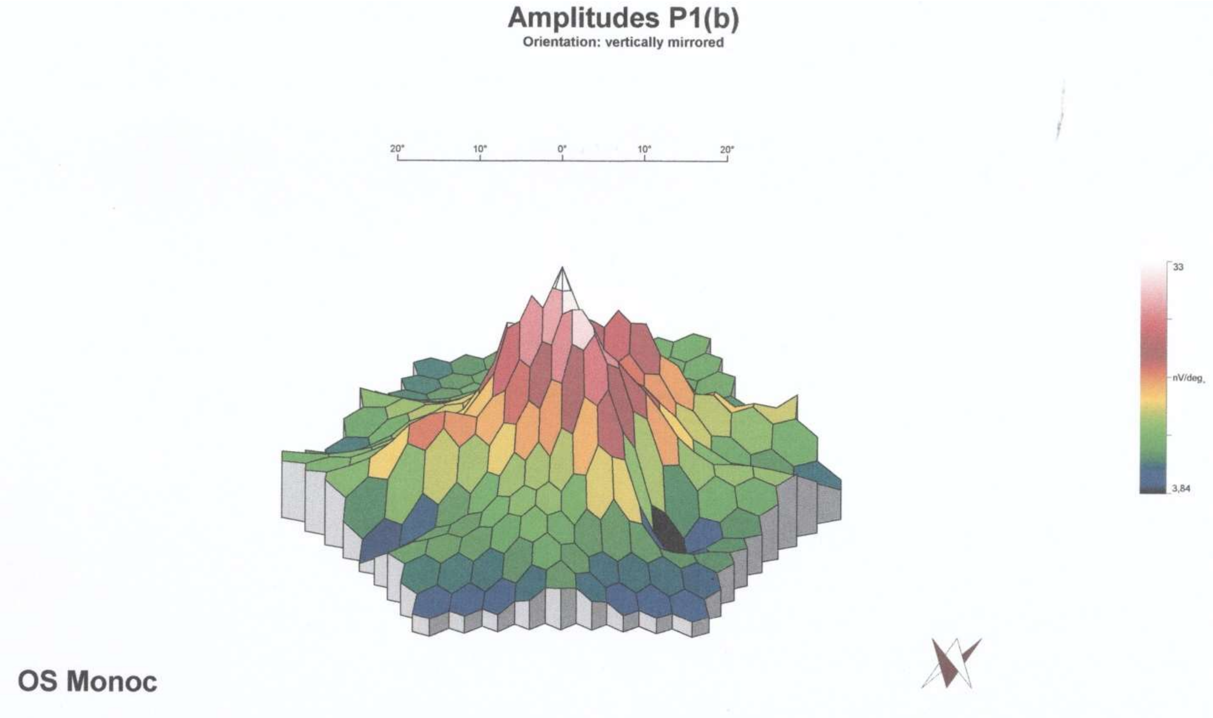

Figure S37. Multifocal ERG 3 D colour map of patient IV/1, OS

photopic 3.0

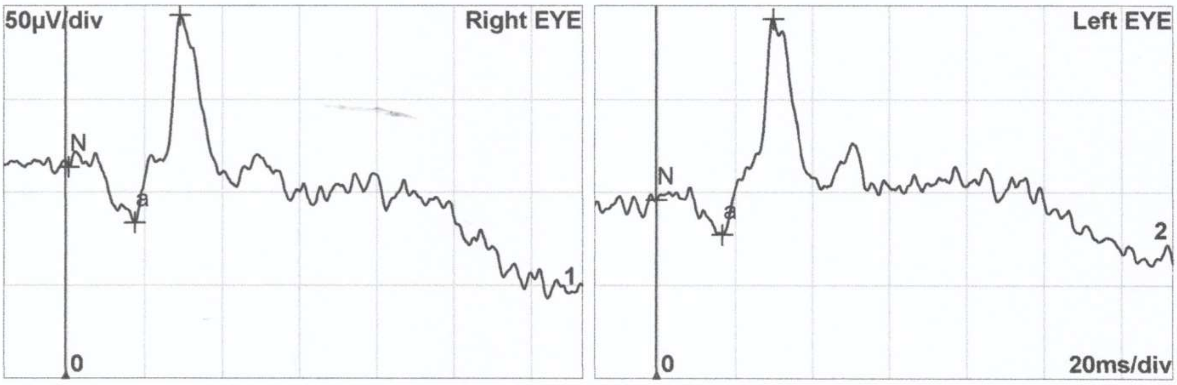

Channel	a [ms]	b [ms]	a-wave	b-wave
1 R-1	18	30	30µV	111µV
2 L-2	17	30	18,7µV	116µV
Normals	-	-		

30Hz Flicker 3.0

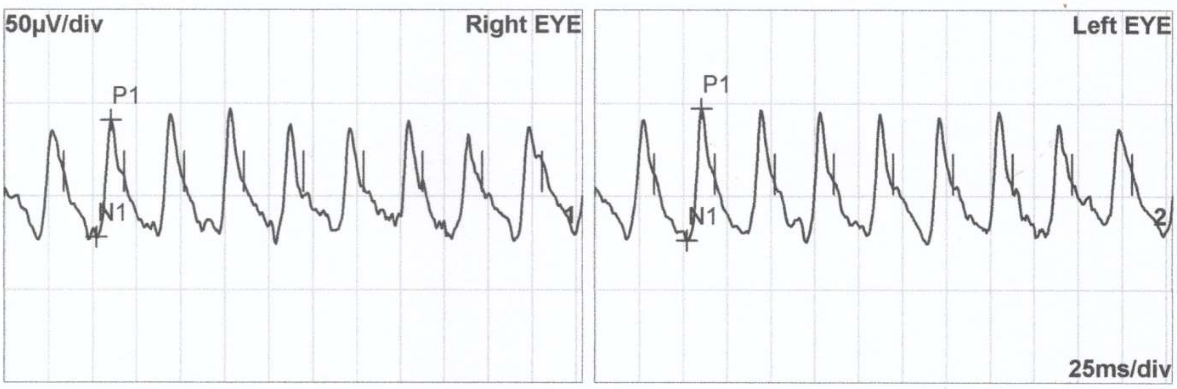

Channel	N1 [ms]	P1 [ms]	N1-P1
1 R-1	53	61	63,1µV
2 L-2	53	61	70,7µV

Figure S38. Standard full-field ERG recording of patient IV/2.

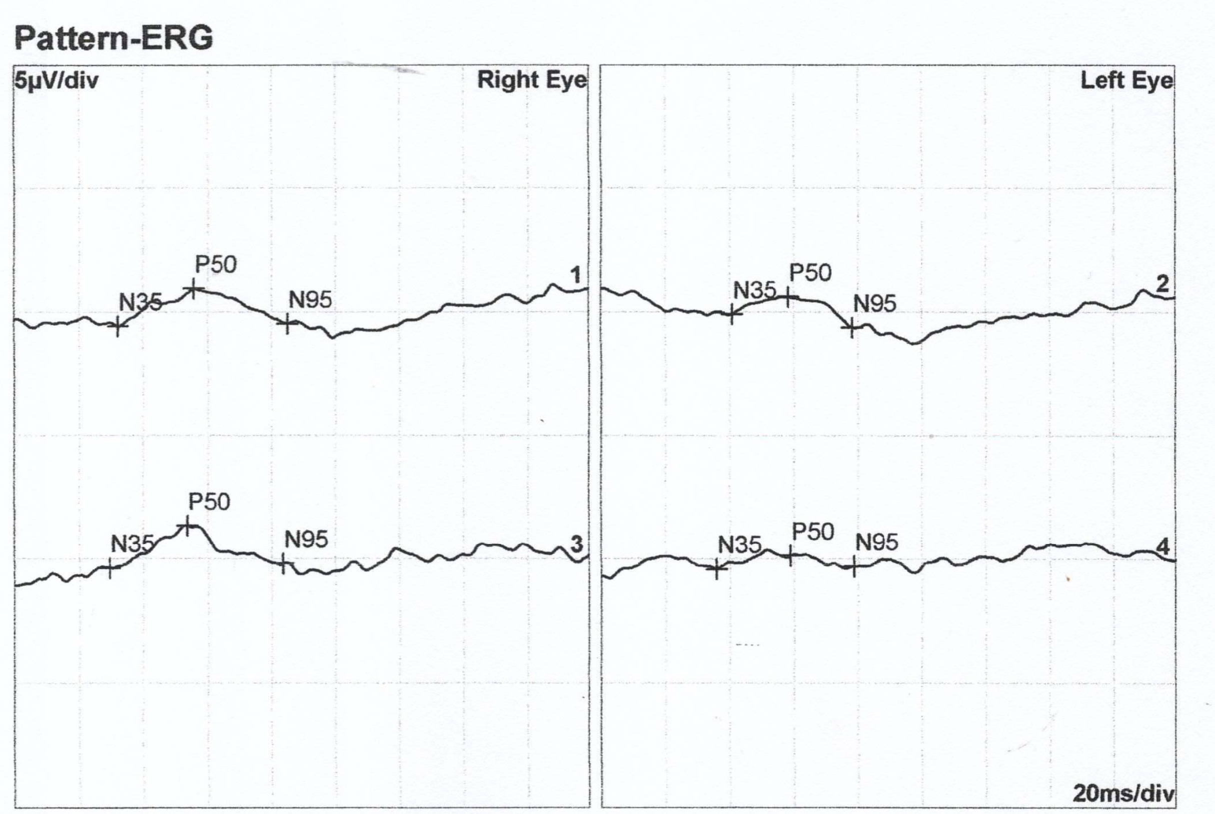

Figure S39. PERG recording of patient IV/2. Lines 1 and 3 and lines 2 and 4 represent pairs of replicate measurements.

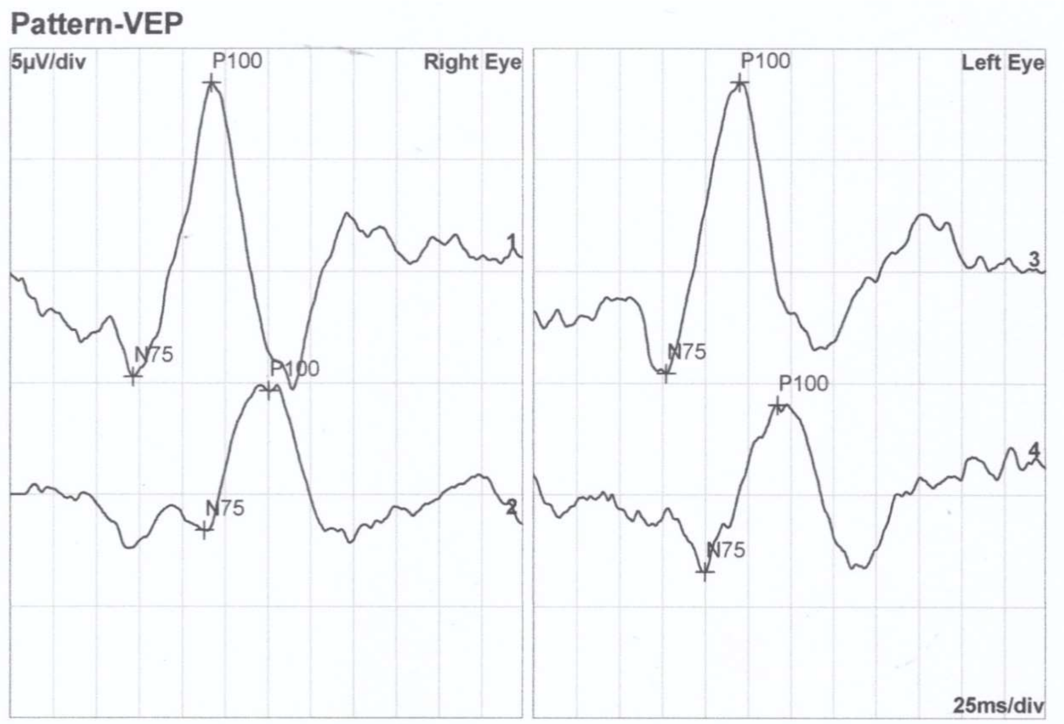

Figure S40. pVEP recording of patient IV/2. Lines 1 and 3 display responses to 60' stimuli and lines 2 and 4 represent responses to 15' stimuli.

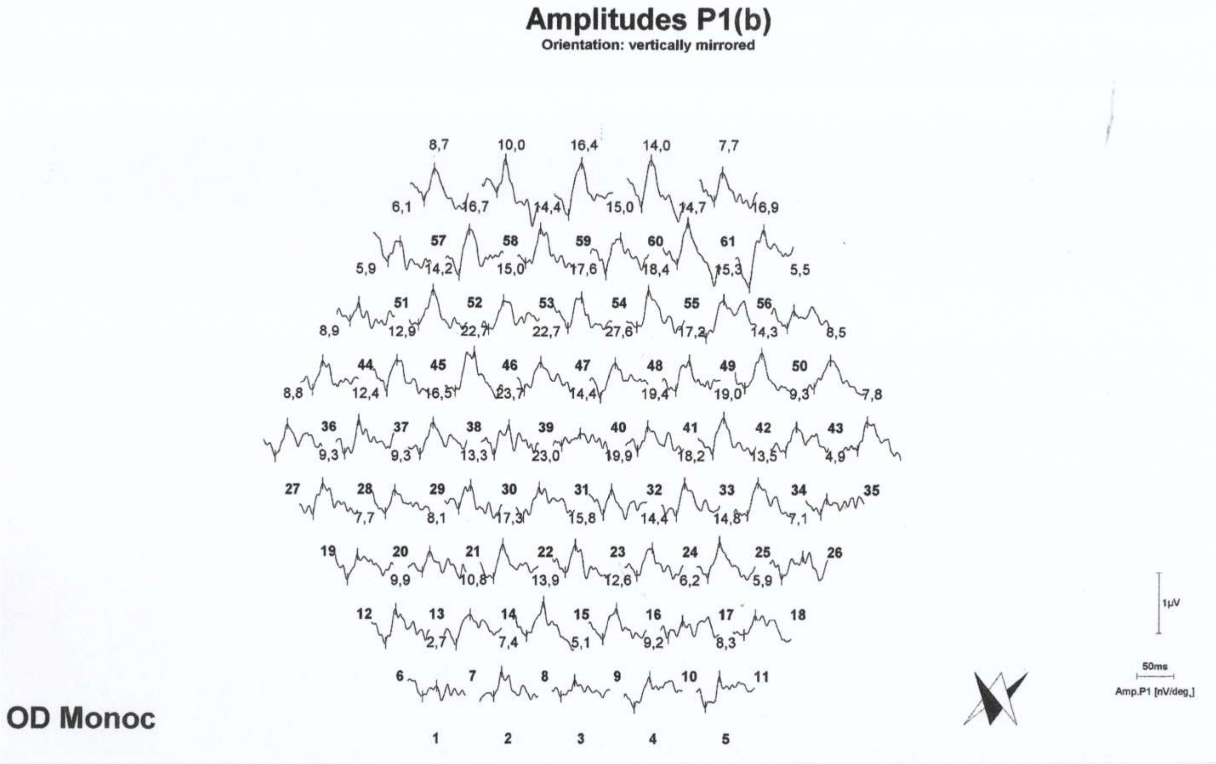

Figure S41. mfERG recording of patient IV/2, OD.

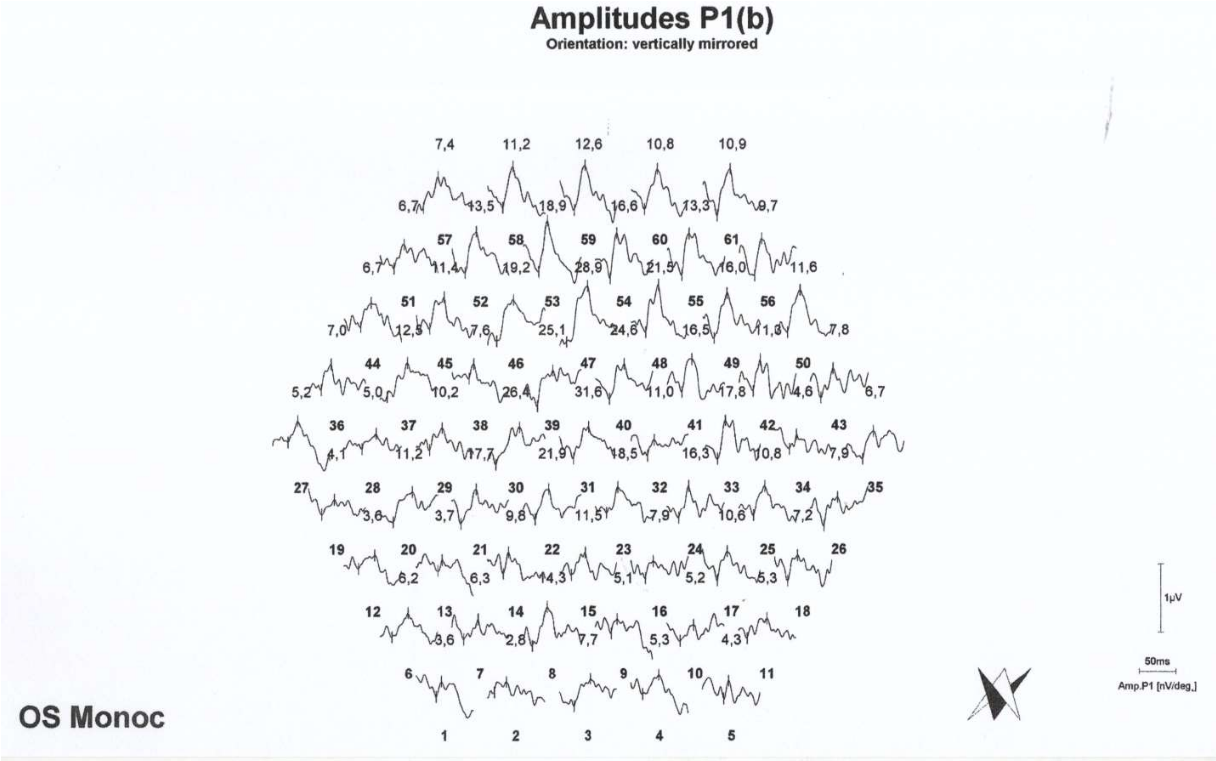

Figure S42. mfERG recording of patient IV/2, OS.

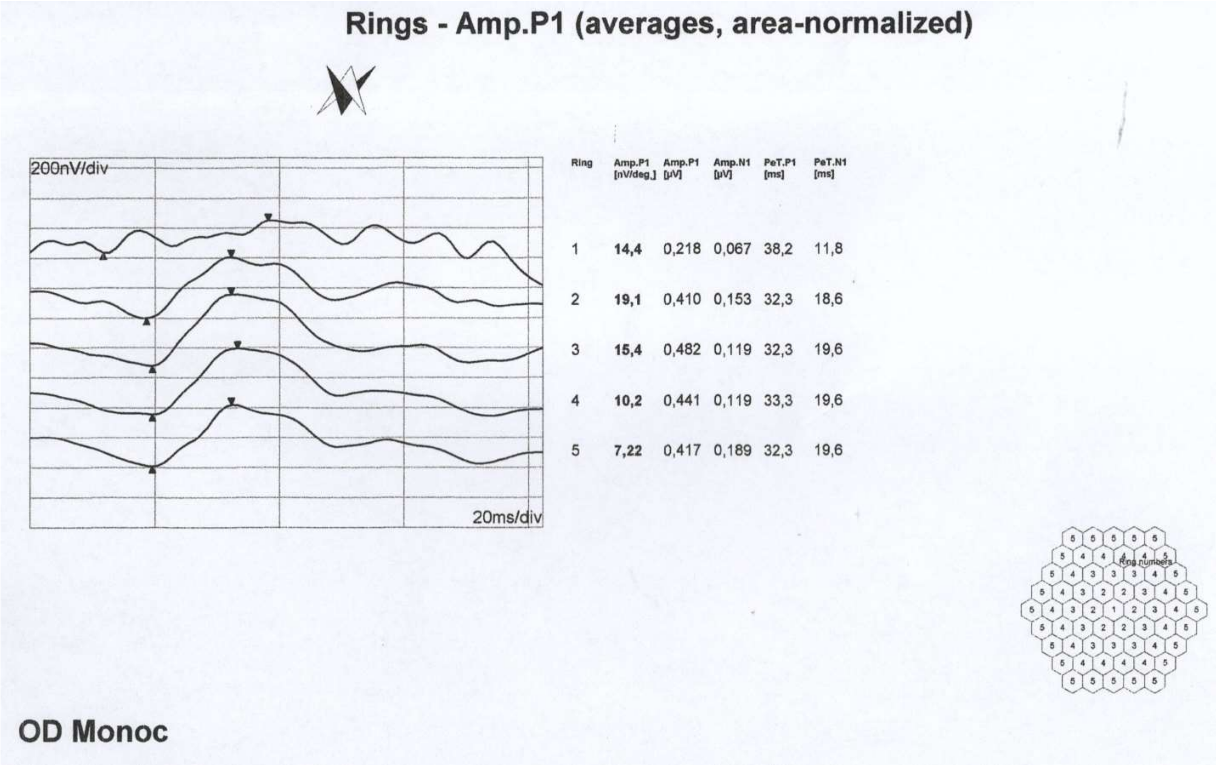

Figure S43. mfERG ring analysis of patient IV/2, OD.

Rings - Amp.P1 (averages, area-normalized)

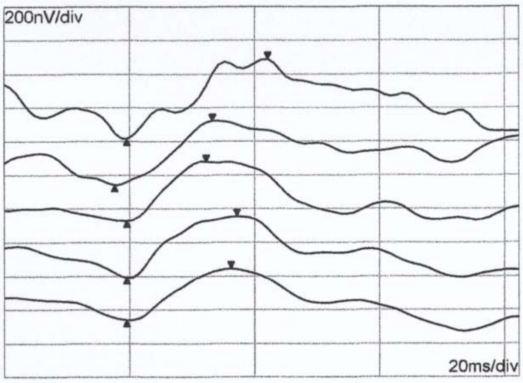

Ring	Amp.P1 [nV/deg.]	Amp.P1 [µV]	Amp.N1 [µV]	PeT.P1 [ms]	PeT.N1 [ms]
1	**31,6**	0,478	0,174	42,1	19,6
2	**17,6**	0,379	0,167	33,3	17,6
3	**11,3**	0,354	0,069	32,3	19,6
4	**8,41**	0,366	0,167	37,2	19,6
5	**5,34**	0,308	0,123	36,3	19,6

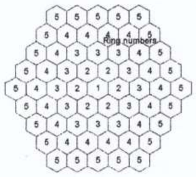

OS Monoc

Figure S44. mfERG ring analysis of patient IV/2, OS.

photopic 3.0

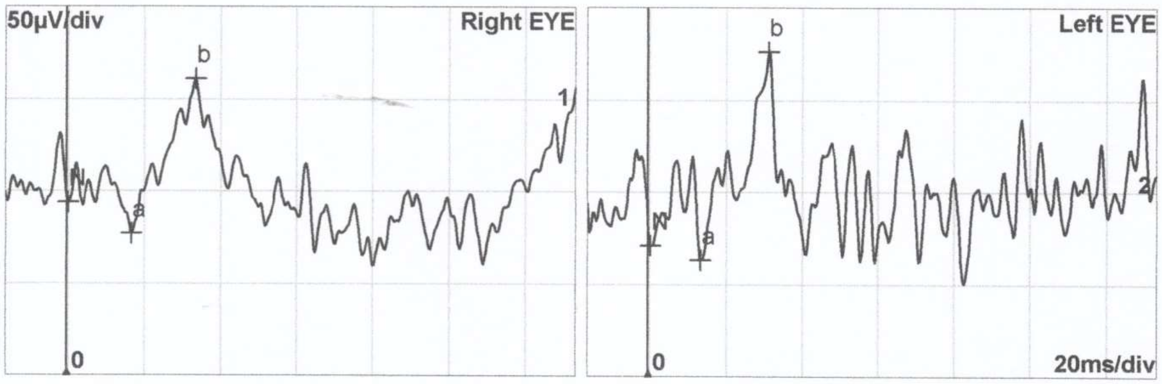

Channel	a [ms]	b [ms]	a-wave	b-wave
1 R-1	17	34	16,6µV	83,9µV
2 L-2	14	32	7,71µV	113µV
Normals	-	-		

30Hz Flicker 3.0

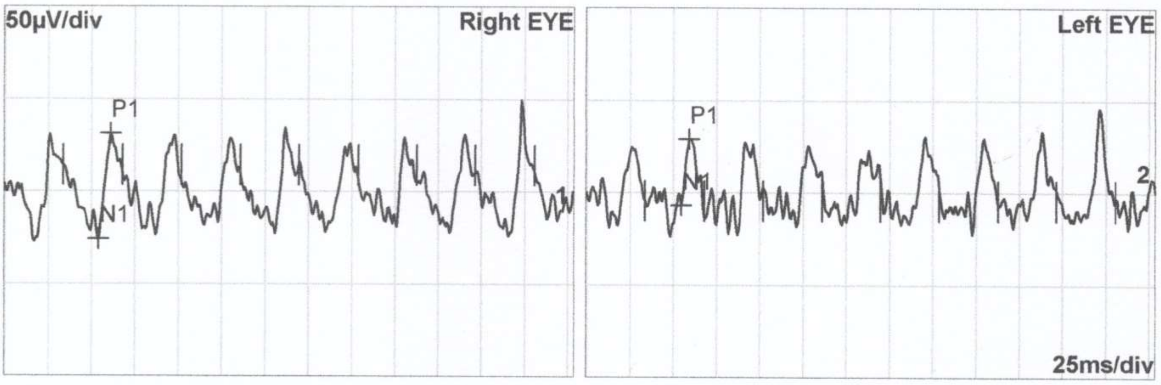

Channel	N1 [ms]	P1 [ms]	N1-P1
1 R-1	55	62	57µV
2 L-2	56	60	35,8µV

Figure S45. Standard full-field ERG recording of patient IV/6.

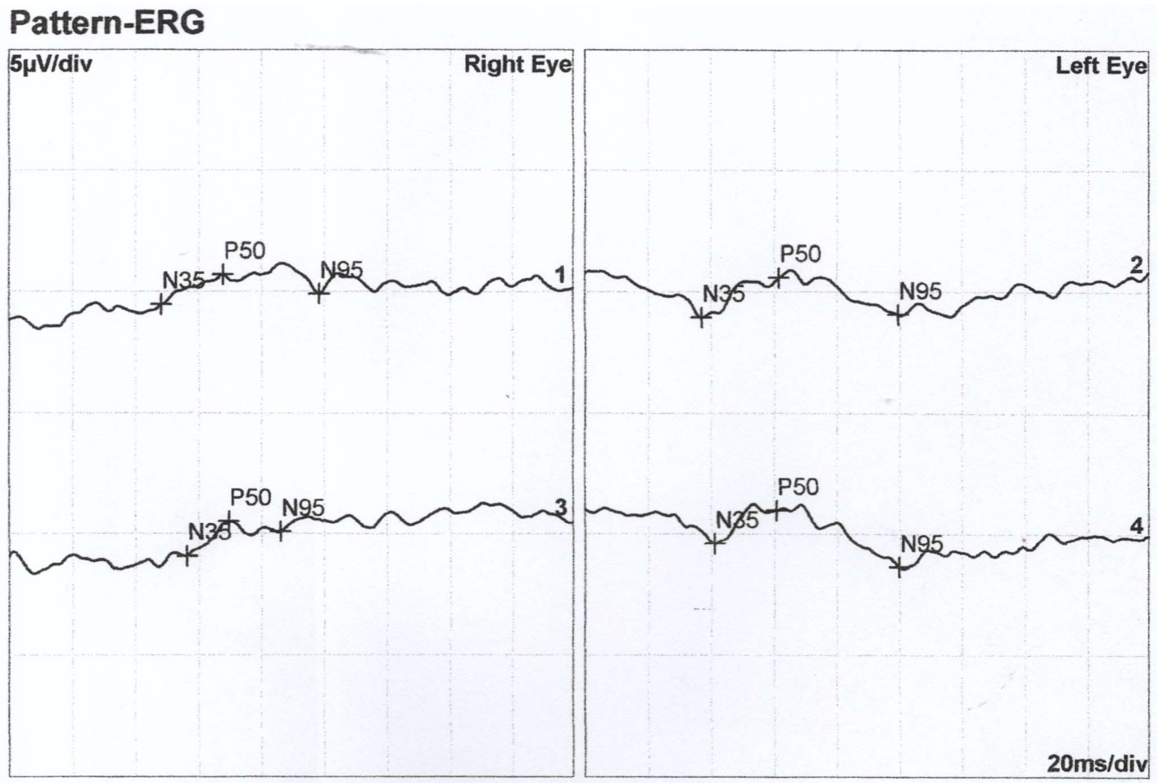

Figure S46. PERG recording of patient IV/6. Lines 1 and 3 and lines 2 and 4 represent pairs of replicate measurements.

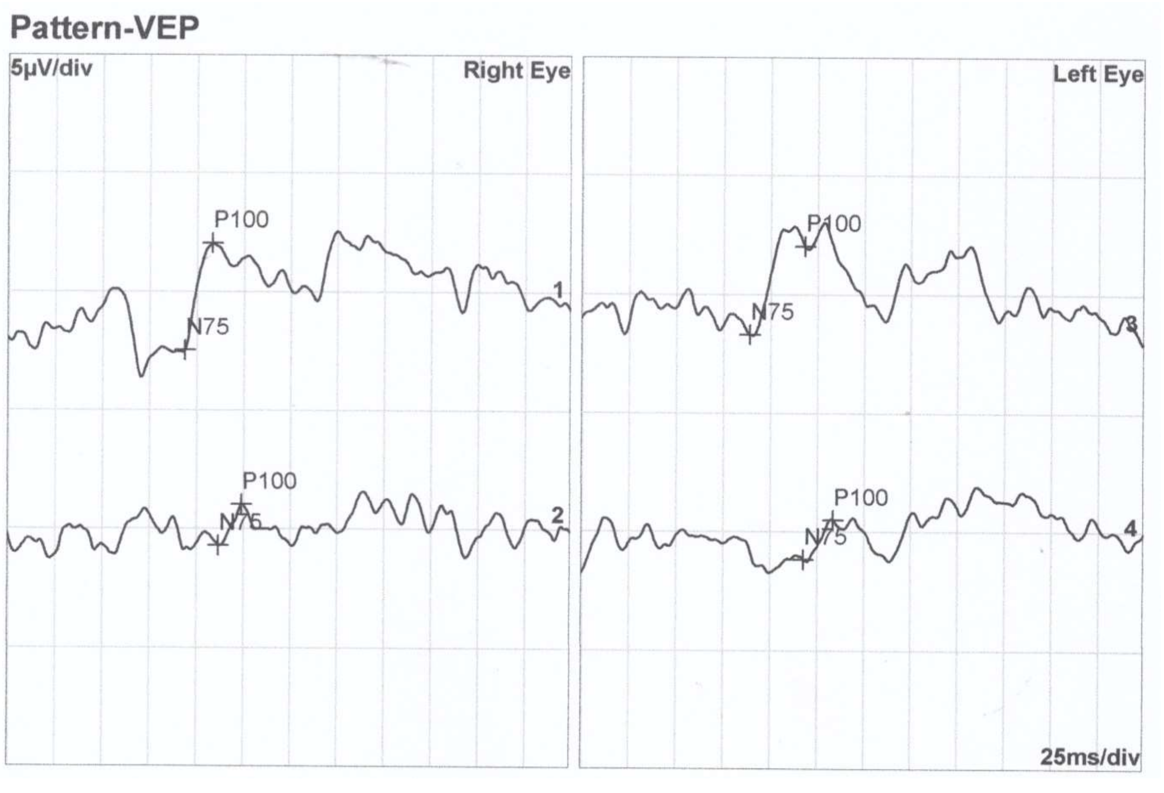

Figure S47. pVEP recording of patient IV/6. Lines 1 and 3 display responses to 60' stimuli and lines 2 and 4 represent responses to 15' stimuli.

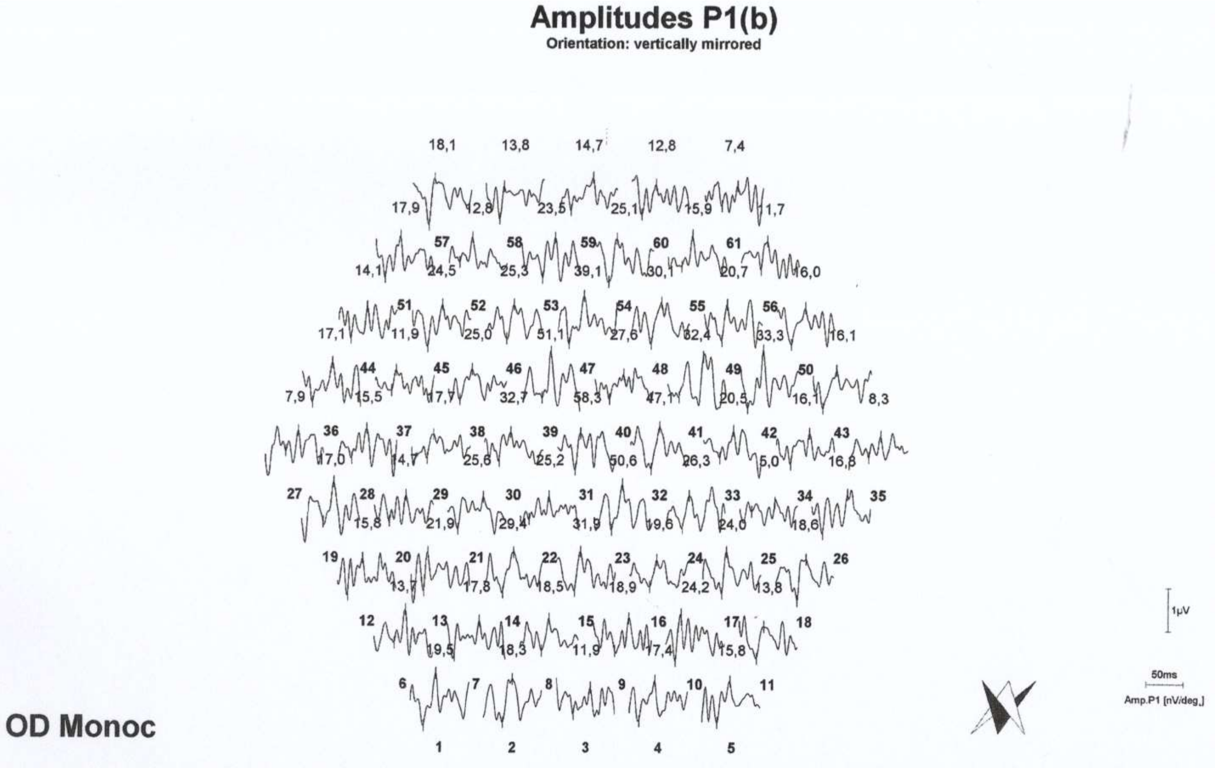

Figure S48. mfERG recording of patient IV/6, OD.

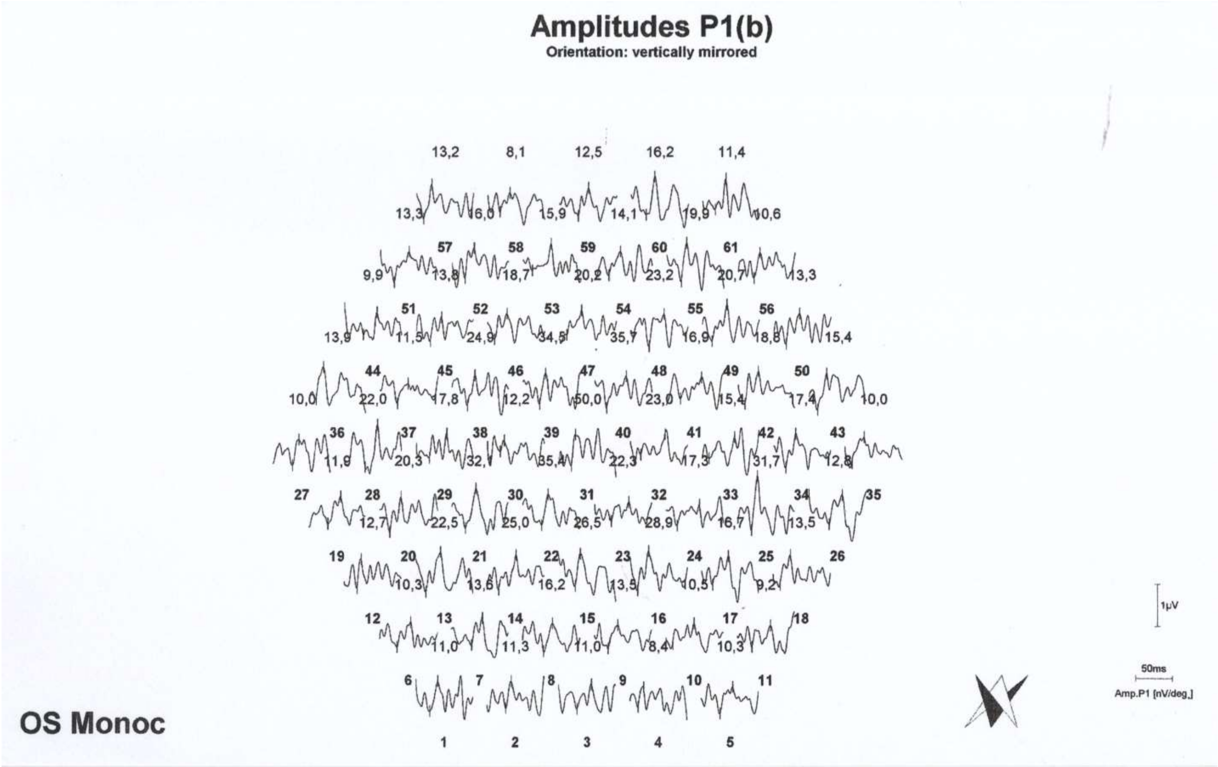

Figure S49. mfERG recording of patient IV/6, OS.

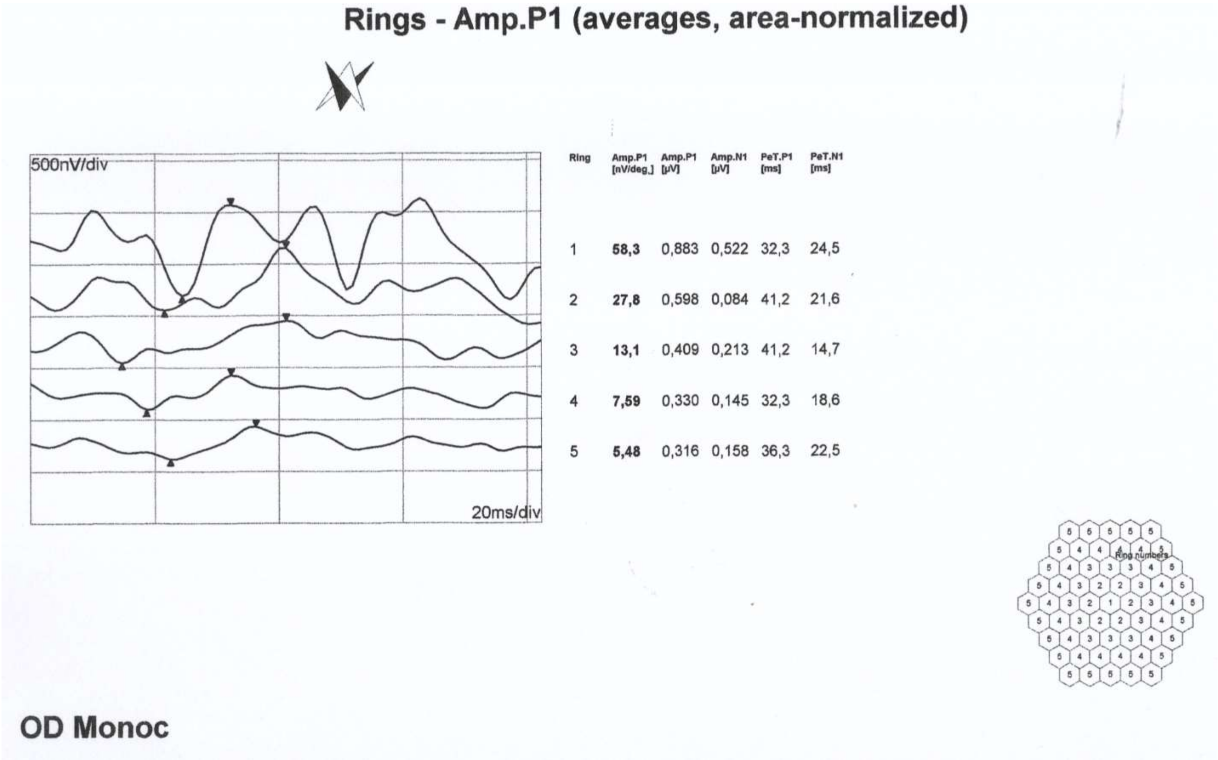

Ring	Amp.P1 [nV/deg.]	Amp.P1 [µV]	Amp.N1 [µV]	PeT.P1 [ms]	PeT.N1 [ms]
1	**58,3**	0,883	0,522	32,3	24,5
2	**27,8**	0,598	0,084	41,2	21,6
3	**13,1**	0,409	0,213	41,2	14,7
4	**7,59**	0,330	0,145	32,3	18,6
5	**5,48**	0,316	0,158	36,3	22,5

Figure S50. mfERG ring analysis of patient IV/6, OD.

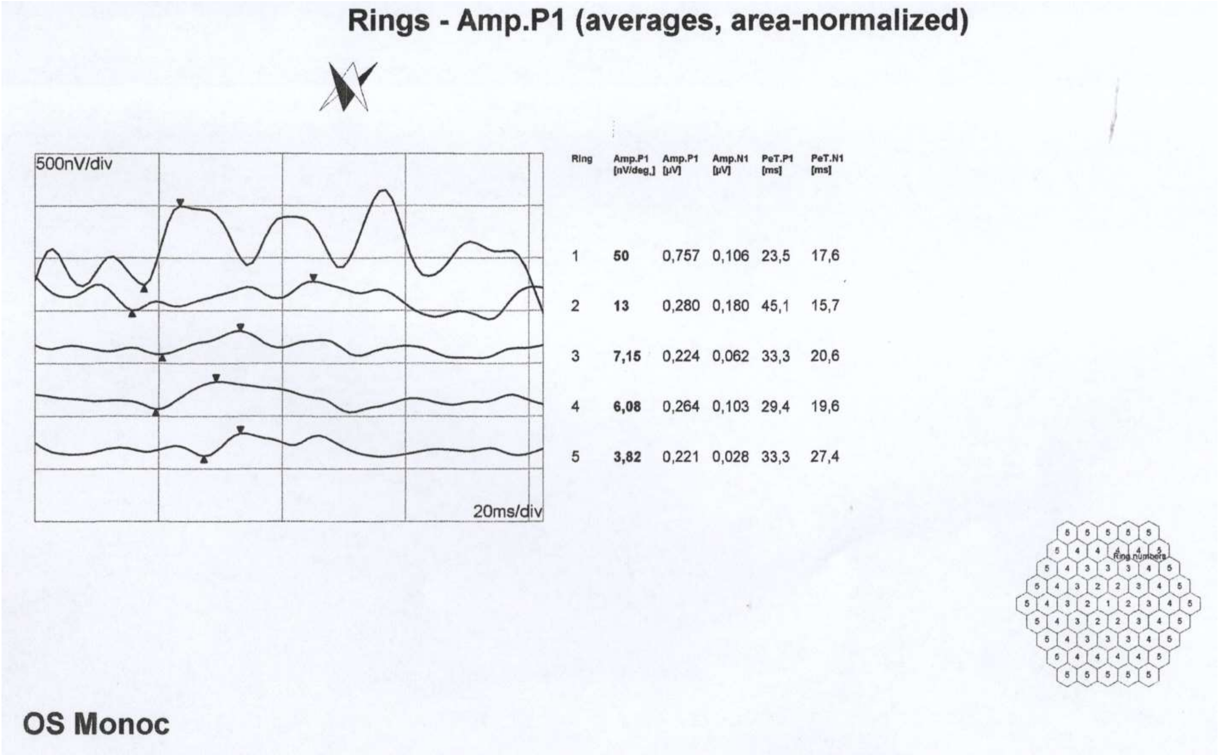

Figure S51. mfERG ring analysis of patient IV/6, OS.

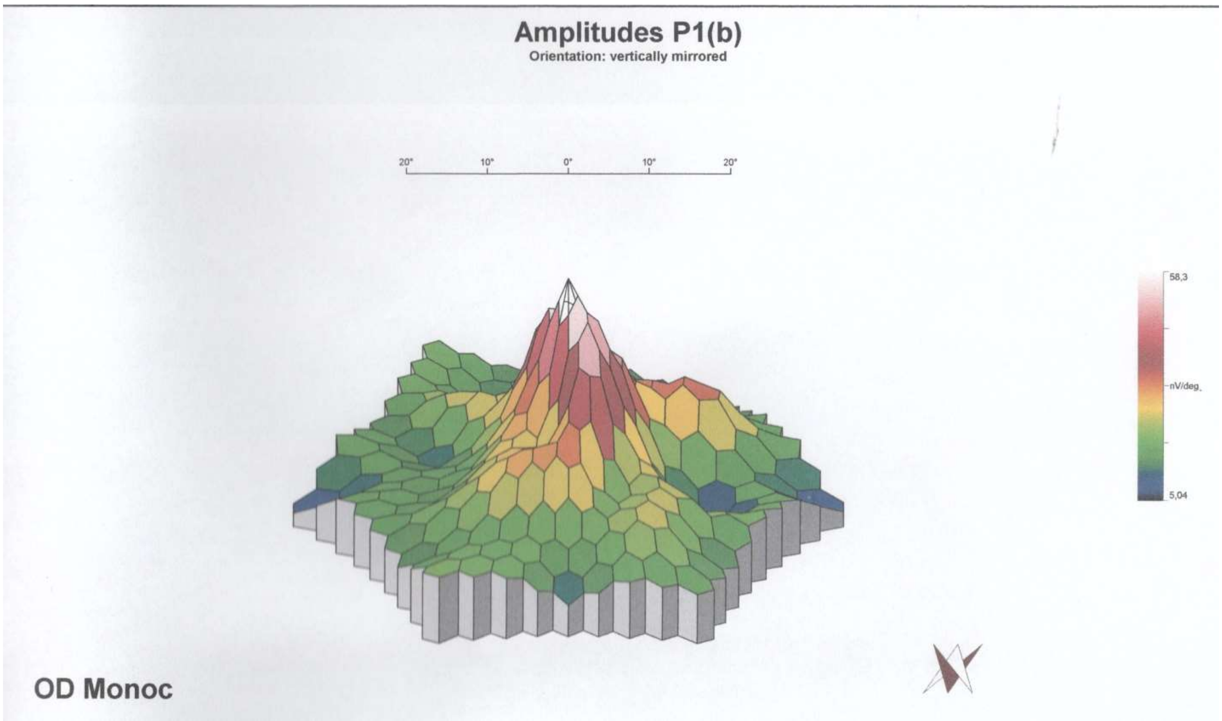

Figure S52. Multifocal ERG 3 D colour map of patient IV/6 severely myopic affected female right eye displays extensive subnormal responses within the whole central 30 degrees.

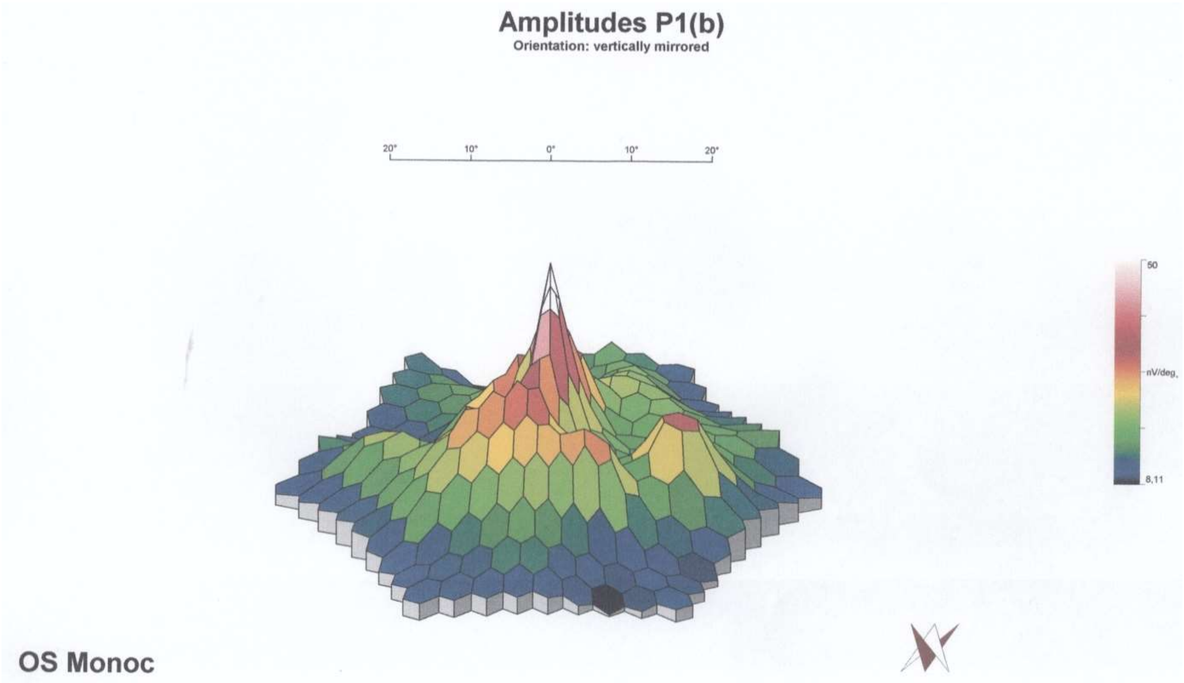

Figure S53. Multifocal ERG 3 D colour map of IV/6 severely myopic affected female left eye displays extensive subnormal responses within the whole central 30 degrees.

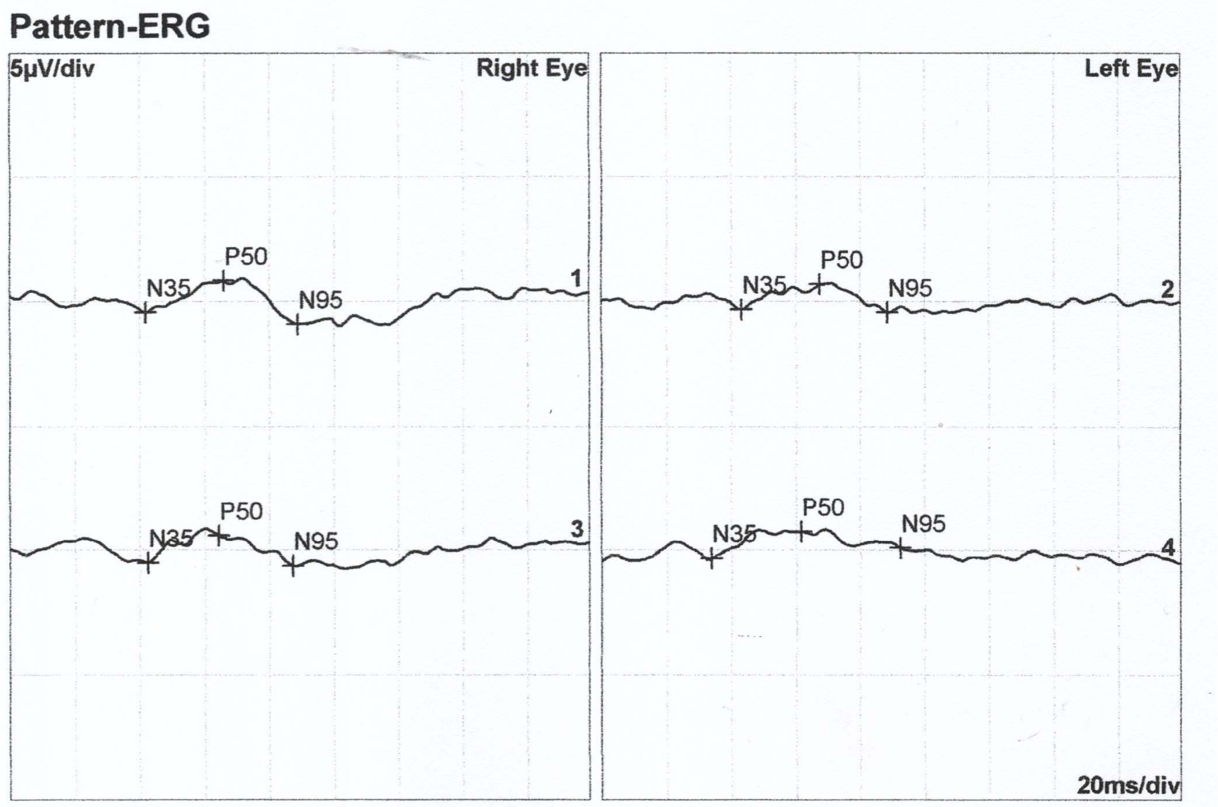

Figure S54. PERG recording of patient IV/10. Lines 1 and 3 and lines 2 and 4 represent pairs of replicate measurements.

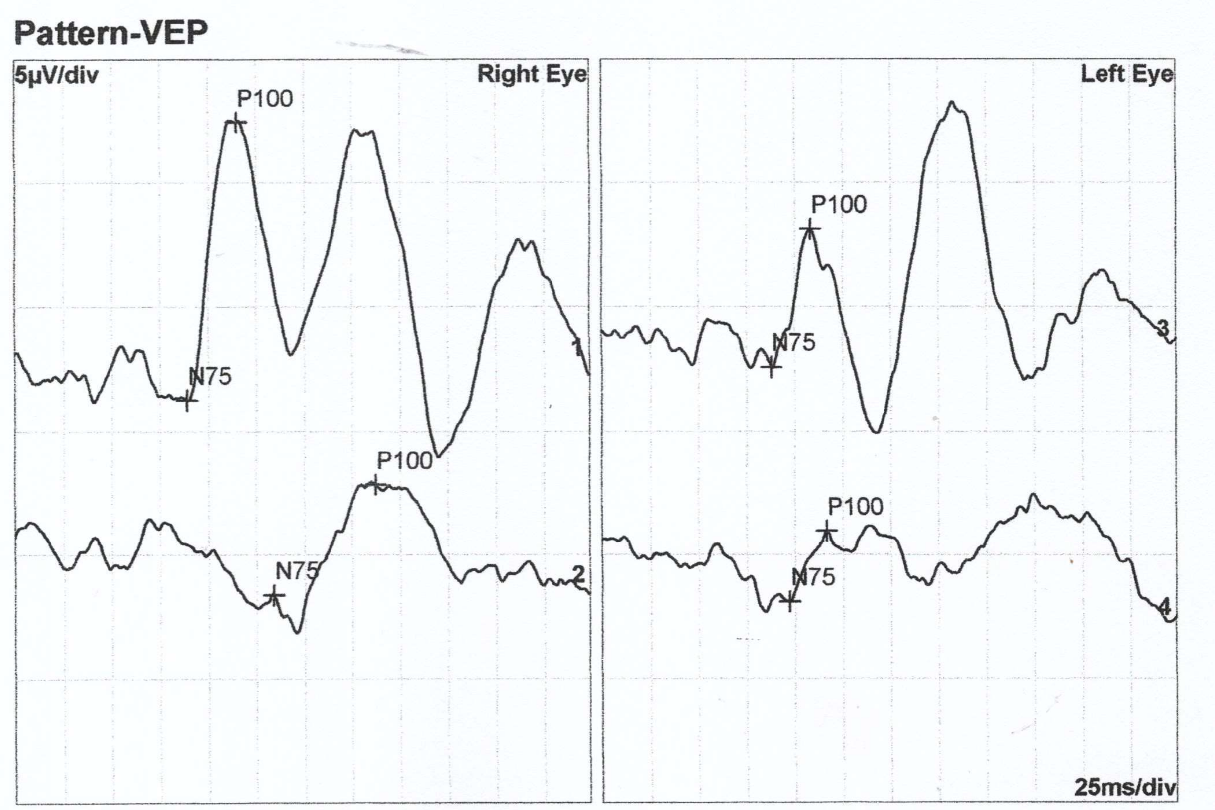

Figure S55. pVEP recording of patient IV/10. Lines 1 and 3 display responses to 60' stimuli and lines 2 and 4 represent responses to 15' stimuli.
