# Supplementary material for: Myopia-26, the female-limited form of early-onset high myopia, occurring in a European family
Source: Orphanet J Rare Dis. 2021 Jan 22;16:45. doi: 10.1186/s13023-021-01673-z (PMC7825233; doi:10.1186/s13023-021-01673-z)
Supplement: Supplementary file 3 — Additional file 3. Ophthalmology findings. Electrophysiology methods. Electrophysiology findings (Pattern VEP, Pattern ERG, Standard full field ERGs, Multifocal ERGs). Colour vision testing. Numerical electrophysiology data: Table S1, Table S2, Table S3. [file 13023_2021_1673_MOESM3_ESM.pdf]

SUPPLEMENTARY TEXT

1. OPHTHALMOLOGY

IOP (intraocular pressure)

We only had the possibility to measure IOPs in one session. Taking into consideration the known fluctuation of IOPs, the measured values were not taken as representative. Also, evaluation of IOPs in high myopic patients is rather questionable because of the high rate of false negative values due to the characteristically thinner corneas of these patients. Therefore these values should be evaluated critically.

VFD (visual field defects)

Visual field measurements are more reproducible than IOPs, and VFDs are robust markers of longer exposures to glaucomatosus damage. We carried out automated kinetic full-field perimetry as a gross screening test of high myopic visual field defects in our patients. The observed VFDs, however were less characteristic of high myopia (HM), but more reminiscent of age-related POAG, for two reasons: i) VFDs showed deterioration with older age (such progression is not characteristic for HM) and ii) VFDs were observed nasally (characteristic of glaucomatous VFD) and not temporally as one would expect for high myopia (1, 2). However, these VFDs did not respect the horizontal meridian, as opposed to a typical glaucomatous damage. Moreover, visual field defects did not clearly correspond with the distribution of the RNFL-losses. (RNFL OCT scans are available for two patients with observed VFDs: affected females III/8 and IV/10, shown on **Figures S4** and **S20 in Supplementary figures I**.)

Fundus appearance
(META-PM classification and optic disc appearance)

Fundus images (taken with either TRC-501X; Topcon digital fundus photography or Optos ultra-wide field fundus photography) were assessed in terms of myopic alterations (according to the META-PM classification) and also in terms of optic disc appearance.

The simplified META-PM classification divides pathologic myopic (PM) lesions into 5 categories including “no myopic retinal lesions” (Category 0), “tessellated fundus only” (Category 1), “diffuse chorioretinal atrophy” (Category 2), “patchy chorioretinal atrophy” (Category 3), and “macular atrophy” (Category 4). Three additional features were added to these categories as extra notes: (I) lacquer cracks, (II) myopic CNV (choroidal neovascularization), and (III) Fuchs spot (3). Myopic fundus alterations in our patients ranged from Category 0 (META-PM 0) to Category 2 (META-PM 2), and these alterations corresponded clearly with the patients’ degree of myopia. No posterior staphyloma was observed in any of the cases.

Optic disc appearances are difficult to interpret in terms of glaucomatous changes in highly myopic eyes due to the marked changes in the optic nerve head appearance by myopia itself (tilted disc etc.) (4). Therefore we cautiously interpret our patients’ optic disc appearances and would abstain from the clear declaration of potential glaucomatous changes.

RNFL OCTs

RNFL OCT scans are available for patients III/3, IV/1, IV/2, III/8 and IV/10. Visual field defects (VFDs) were observed in patients III/8 and IV/10 (**Figures S5** and **S21**, respectively), therefore analysis of the correlation of RNFL losses with VFDs was possible in these two cases. However, in the case of patient III/8 the RNFL scans cannot be interpreted appropriately due to the inappropriate default interface identification (**Figure S4**), whereas in case of patient IV/10 (**Figure S20**) the distribution of RNFL loss does not clearly correlate with the observed VFD. In summary, RNFL OCTs do not correlate with VF alterations and do not support the potential existence of POAG in these patients.

Macular OCTs

Macular OCT scans revealed thinner or incipient atrophic sensory retina in patients with higher degrees of myopia and a META-PM 1-2 category fundus appearance. No posterior staphyloma was observed in any of the cases.

OPHTHALMOLOGY SUMMARY

Fundus, OCT and visual field alterations showed no characteristics of cone dystrophy, such as „bull's eye" appearance on the central fundus, outer retinal changes with OCT or a central scotoma with visual field testing. Rather they were characteristic of high myopia: META-PM1-2 fundus appearance and thinner or incipient atrophic sensory retina on macular OCT scans.

Despite that the possibility of an association of POAG with high myopia in our patients arose, available data do not provide sufficient and inarguable evidence to support the diagnosis of POAG at present. Long- term follow-up would be necessary to reveal any evidence of potential progression of these parameters that could also be expected in glaucoma.

2. ELECTROPHYSIOLOGY

METHODS

Standard full-field and multifocal ERGs were performed with fully dilated pupils, after half an hour dark adaptation for standard ERGs. For multifocal ERGs (mfERGs) the stimulus consisted of 61 scaled hexagons covering the central 30◦ of the visual field. DTL fiber corneal electrodes were used to detect electric signals for the ERGs (standard, multifocal and pattern). Black and white reversal checkerboard stimulus was used for pattern visual evoked potential (VEP) and pattern ERG (PERG) tests, the check size was 60' (1°) and 15' (0.25°) for VEP and 48' (0.8°) for PERG recordings, respectively; whereas the stimulus field size was 15°. Refractive errors were corrected for the viewing distance before mfERG, PERG and pattern VEP tests.

ELECTROPHYSIOLOGY FINDINGS

pattern VEP

BACKGROUND:

Visual evoked potentials are the measure of the integrity of the visual pathway from the retina to the occipital cortex. The optic nerve is the primary structure examined (5)**,** and a delayed P100 often occurs in association with an optic nerve disease. Latencies, however may be also commonplace in macular dysfunction (6),as the visual cortex is activated primarily by the central visual field (7). Therefore a delayed VEP cannot be considered pathognomic of optic nerve disease, and in order to fully evaluate an abnormal VEP an associated test of macular function, such as PERG or mfERG is needed (6). Stimulation with smaller checks (15’) better represent the central vision and is more sensitive in detecting visual system defects, (i.e. responses are disturbed in earlier stages of visual system defects already); whereas stimulation with larger checks (60’) represent more the peripheral vision, and produces more variable responses, compensating for decreased visual acuity (VA), and accordingly detecting larger scale visual system defects in a later stage already (5)**.**

RESULTS for our patients:

1. P100 latency (or implicit time) was significantly increased in nearly all cases as compared to normal controls (t test: $p<.00005$ for 60’ and $p<.00001$ for 15’).
2. P100 implicit times to 15’ stimulation were significantly more delayed than responses to 60’ stimulations (t test: $p<.001$).
3. No significant correlation of P100 delay with either VA or the refractive error (SE) could be detected for our patients.

DISCUSSION:

1. pVEP results, as evaluated together with with reduced PERG and mfERG responses, reflect a **central macular deficit** in our patients with ARR3 mutation.
2. Hypothetically, one could attribute the discrepancy between responses to 15’ and 60’ stimulations to the differences in patients’ VA (spatial resolution). However, as no correlation could be evidenced between patients’ VA, SE, age or affected/ carrier genetic status and the pVEP results, these alterations are most probably **attributable** not to the patients’ high myopia, but rather **to the genetic mutation** in ARR3 evidenced in all these patients- irrespective of their VA, SE or affected/ carrier genetic status.

pattern ERG

BACKGROUND:

Transient PERG is an objective measure of macular dysfunction (P50) and also allows the direct assessment of RGC activity (N95) (8). However, it naturally depends on the integrity of the both the input and output structures (photoreceptors, bipolar cells, interneurons) as well. The late component, N95 originates solely from the spiking activity of RGCs, and is abolished if RGC function is blocked by drugs (pharmacological blocking) or by some diseases such as glaucoma (9). The P50 component is generated before spiking activities of the RGCs arise, it originates from the non-spiking activity of the retina, and can be accordingly altered in several retinal/macular conditions reflecting some kind of macular dysfunction (macular degeneration, myopic maculopathy, diabetic retinopathy). At the same time, however, all the disturbances of the input structures of RGCs will naturally also affect N95. Therefore an isolated RGC dysfunction could be evidenced only in case of a normal P50 together with an abnormal N95. In contrast, a general PERG disturbance more probably reflects a macular dysfunction.

RESULTS for our patients:

1. Amplitudes of both the P50 and N95 waves were significantly reduced as compared to normal controls. (t test: $p<.000001$ for both) In numerous cases the amplitudes of P50 and N95 waves were reduced to the nanovolt domain, which implies extremely low or even undetectable responses.

2. The amplitudes of P50 and N95 waves were reduced in our patients with ARR3 mutation to mean values of 29.8 % and 20.8 % of the controls, respectively, and the difference of the extent of their reduction was significant (t test: $p<.005$).

3. There was also a statistically significant difference between the measure of reduction in mfERG and PERG responses, i.e. the amplitudes of N95 were reduced in our patients with ARR3 mutation to mean values of 20.8 % of the controls, the amplitudes of R1, R2, R3, R4 and R5 were reduced to an overall mean of 40.2%. The difference in the extent of their reduction was highly significant (t test: $p<1E-9$).

DISCUSSION:

1. The significant, robust general PERG disturbance along with mfERG alterations seen for our patients with ARR3 mutation reflect a macular dysfunction.

2. The significant discrepancy between the extent of reduction in amplitudes of the P50 and N95 waves of PERG along with the significant difference between mfERG and PERG disturbances, however (PERGs are more prominently reduced than mfERGs are) may point to a disturbance inherent **also** to the RGCs themselves (inner retinal, postreceptoral problem) besides a receptoral problem originating from the photoreceptor cells.

Standard full-field ERG

BACKGROUND:

The first three ERG recordings under scotopic conditions are dominated by and mainly represent the rod system, however only the first one (DA 0.01) is exclusively generated by the rod system, and the remaining two (DA 3.0, DA 10/30) are a mixed response of the rod and cone function. The last two light adapted ERG responses to single flash and flicker stimuli (LA 3.0 and LA 30 Hz) in contrast are driven by the cone system (10). Cone photoreceptor function is therefore best assessed by these two photopic ERG recordings. Full-field ERG is, however a mass response of the retina, and is largely generated by the retinal periphery with only minimal contribution from the macula (11). Accordingly, a purely central alteration (macular dysfunction)is very often masked by the spared paracentral/peripheral responses, and in such cases full-field ERGs are normal (6). Therefore the electrophysiological assessment of macular function requires the use of different techniques such as the pattern ERG or multifocal ERG (11).

RESULTS for our patients:

Both scotopic and photopic responses were normal, indicating an overall normally functioning cone system.

DISCUSSION:

1. A general cone system dysfunction could not be evidenced in our patients with ARR3 mutation, in contrast to that seen in animal models (12).

2. Taken together with the PERG and mfERG results, which were both reduced in amplitude, full-field ERGs in our patients point to a central rather than general alteration of the cone system.

Multifocal ERGs

BACKGROUND:

Similarly to PERG, multifocal electroretinography (mfERG) is also an index of the central, cone-driven retinal function. However, in contrast to PERG, mfERG is flash-stimulated and provides additional spatial information of localized retinal areas (6).

RESULTS for our patients:

Trace arrays with 61 hexagons were analysed in the form of a ring analysis for our patients.

1. In each ring (1-5) there was a significant reduction in amplitudes as compared to normal controls (t tests: $p<.000005$ for R1, $p<.000001$ for R2 to R5).

2. There was no significant difference between any pairs of the individual rings in amplitude as evidenced by analysis of variance (ANOVA).

3. There was no significant correlation between the amplitude and the patients' VA or SE within each individual ring.

DISCUSSION:

1. MfERGs indicated a central macular deficit in our patients with ARR3 mutation along with significantly reduced PERG recordings.

2. There were no spatial differences in alteration within the central 30° of the macular area as evidenced by the similarly reduced responses in rings 1 to 5.

3. These alterations –similarly to pVEP alterations-are most probably also attributable to our patients' genetic defect (ARR3 mutation) rather than to their high myopia, as these alterations showed no correlation with either the VA or the SE.

Additional point

There was no evidence of posterior staphyloma in any of our patients, as demonstrated by the representative range of macular OCT scans (**Figures S3, S7, S11, S16, S19**) and fundus images (**Figures S2, S6, S10, S15, S18, all in Supplementary figures I**), that would have interfered with the interpretation of the electrophysiology tests by distorting the projected stimuli.

ELECTROPHYSIOLOGY SUMMARY

Standard full-field ERG, pVEP, PERG and mfERG results altogether indicated a central macular dysfunction in our patients with ARR3 mutation, rather than a general cone system disturbance as evidenced earlier in animal model (12). Both the inner and outer retinal structures of the central retina seem to be affected according to the electrophysiology test results, and these alterations are most probably attributable to the genetic defect evidenced in our patients, rather than to their high myopic refractive error.

3.COLOUR VISION TESTING

METHODS:

Colour vision testing was accomplished using the Lanthony Desaturated Panel Test (Lanthony D-15).

RESULTS:

Lanthony D-15 colour vision testing consistently revealed a diffuse colour discrimination defect in all investigated patients.

DISCUSSION:

Diffuse colour discrimination defect (with no specific colour vision vector) points to and stands in accordance with the macular dysfunction evidenced by PERG, mfERG and pVEP tests in our patients with ARR3 mutation.

SUPPLEMENTARY TABLES

Table S1. Numerical data of pVEP analyses

ID	pVEP N75 lat 60’(ms)	pVEP N75 lat 15’(ms)	pVEP P100 lat 60’(ms)	pVEP P100 lat 15’(ms)	pVEP P100 amp 60’(µV)	pVEP P100 amp 15’(µV)
III/3-R	76	102	104	137	2.41	1.33
III/3-L	85	137	107	168	1.54	2.7
IV/1-R	72	114	118	151	13.2	6.24
IV/1-L	78	101	121	143	13.1	7.47
IV/2-R	80	112	113	151	10.4	3.99
IV/2-L	81	119	113	146	10.8	2.15
IV/6-R	95	113	109	125	4.55	1.75
IV/6-L	90	119	119	134	3.74	1.67
IV/7-R	107	102	128	124	0.72	0.975
IV/7-L	75	90	119	104	2.7	0.809
III/8-R	80	87	101	136	2.41	0.164
III/8-L	77	89	114	109	6.76	4.84
IV/10-R	90	135	116	188	11.2	4.45
IV/10-L	89	98	109	117	5.59	2.86
**V/6-R**	**73**	**86**	**104**	**111**	**17.9**	**17.6**
**V/6-L**	**73**	**87**	**108**	**115**	**16.8**	**18.7**
Mean of lab controls	70.14	76.9	101.55	105.9	11.09	13.85
Control minimum					4.57	3.51
Control maximum	83	85	110	115.7		

lat: latency

amp: amplitude

patient V/6 (marked in green) is a healthy control

Table S2. Numerical data of PERG analyses. Each eye of each patient was measured twice.

ID	PERG N35 lat 1. (ms)	PERG N35 lat 2. (ms)	PERG P50 lat 1. (ms)	PERG P50 lat 2. (ms)	PERG N95 lat 1. (ms)	PERG N95 lat 2. (ms)	PERG P50 amp 1. (µV)	PERG P50 amp 2. (µV)	PERG N95 amp 1. (µV)	PERG N95 amp 2. (µV)
III/3-R	38	32	51	52	73	74	0.734	0.706	0.241	0.0408
III/3-L	27	37	58	54	78	64	1.51	0.958	0.42	0.0703
IV/1-R	39	45	54	63	67	71	0.988	1.43	0.713	1.45
IV/1-L	41	36	52	44	61	61	1.53	0.594	2.47	0.654
IV/2-R	33	30	56	54	86	84	1.54	1.72	1.43	1.55

IV/2-L	41	36	59	60	79	79	0.706	0.525	1.26	0.422
IV/6-R	49	57	68	70	99	87	1.25	1.43	0.798	0.43
IV/6-L	37	42	62	61	100	100	1.66	1.39	1.51	2.32
IV/7-R	49	56	72	66	92	73	0.741	0.646	1.59	0.825
IV/7-L	54	50	69	69	95	98	1.14	0.828	0.85	1.82
III/8-R	47	48	70	63	94	76	1.02	0.715	1.15	1.01
III/8-L	34	36	67	68	89	92	2.17	1.52	1.62	2.11
IV/10-R	42	43	67	65	89	88	1.28	1.11	1.75	1.23
IV/10-L	44	43	68	62	89	93	1.02	1.09	1.14	0.647
**V/6-R**	**32**	**30**	**55**	**54**	**91**	**92**	**3.04**	**3.48**	**7.4**	**6.92**
**V/6-L**	**39**	**36**	**59**	**53**	**86**	**83**	**2.87**	**2.73**	**3.22**	**4.58**
Mean of lab controls	29.29		50.57		90.22		3.83		5.42	
Control minimum							2.25		2.58	
Control maximum	115		55		99					

lat: latency

amp: amplitude

patient V/6 (marked in green) is a healthy control

Table S3. Numerical data of mfERG analyses

ID	**mf ERG R1 µV**	**mf ERG R2 µV**	**mf ERG R3 µV**	**mf ERG R4 µV**	**mf ERG R5 µV**
III/3-R	50.4	17.6	11.9	6.72	6.32
III/3-L	35.5	12	8.59	5.79	5.26
IV/1-R	27.7	14.1	9.88	5.35	4.32
IV/1-L	33	12.2	9.51	3.47	3.76
IV/2-R	14.4	19.1	15.4	10.2	7.22
IV/2-L	31.6	17.6	11.3	8.41	5.34
IV/6-R	58.3	27.6	13.1	7.59	5.48
IV/6-L	50.1	13	7.15	6.08	3.69
IV/7-R	27.5	18.3	10.4	7.55	5.56
IV/7-L	29.1	11.2	7.98	6.2	4.61
Mean of lab controls	80.88	42.59	25.39	16.98	13.7
Control minimum	42.5	29.1	18.1	12.3	9
Control maximum	115	58	39.4	28.2	25.5

R1 to R5 represent ring numbers in the ring analysis

References

1. Nitta K, Sugiyama K, Wajima R, Tachibana G. Is high myopia a risk factor for visual field progression or disk hemorrhage in primary open-angle glaucoma? Clin Ophthalmol. 2017;11:599-604.
2. Ballon BJ, Echelman DA, Shields MB, Ollie AR. Peripheral visual field testing in glaucoma by automated kinetic perimetry with the Humphrey Field Analyzer. Arch Ophthalmol. 1992;110(12):1730-2.
3. Ohno-Matsui K. Pathologic myopia. Annals of Eye Science. 2018;3(2):8.
4. Jonas JB, Panda-Jonas S, Ohno-Matsui K. Glaucoma in High Myopia. In: Ang M, Wong TY, editors. Updates on Myopia: A Clinical Perspective. Singapore: Springer Singapore; 2020. p. 241-55.
5. Drislane FW. Visual Evoked Potentials. In: Blum AS, Rutkove SB, editors. The Clinical Neurophysiology Primer. Totowa, NJ: Humana Press; 2007.
6. Holder GE. Electrophysiological assessment of optic nerve disease. Eye (Lond). 2004;18(11):1133-43.
7. Odom JV, Bach M, Brigell M, Holder GE, McCulloch DL, Tormene AP, et al. ISCEV standard for clinical visual evoked potentials (2009 update). Doc Ophthalmol. 2010;120(1):111-9.
8. Bhatt D. Electrophysiology for ophthalmologist (A practical approach). Journal of Clinical Ophthalmology and Research. 2013;1(1):45-54.
9. Luo X, Frishman LJ. Retinal Pathway Origins of the Pattern Electroretinogram (PERG). Investigative Ophthalmology & Visual Science. 2011;52(12):8571-84.
10. McCulloch DL, Marmor MF, Brigell MG, Hamilton R, Holder GE, Tzekov R, et al. ISCEV Standard for full-field clinical electroretinography (2015 update). Doc Ophthalmol. 2015;130(1):1-12.
11. Robson AG, Nilsson J, Li S, Jalali S, Fulton AB, Tormene AP, et al. ISCEV guide to visual electrodiagnostic procedures. Doc Ophthalmol. 2018;136(1):1-26.
12. Deming JD, Pak JS, Brown BM, Kim MK, Aung MH, Eom YS, et al. Visual Cone Arrestin 4 Contributes to Visual Function and Cone Health. Investigative Ophthalmology & Visual Science. 2015;56(9):5407-16.
